# Supplementary material for: Synthesis and Biological Evaluation of Novel Fusidic Acid Derivatives as Two-in-One Agent with Potent Antibacterial and Anti-Inflammatory Activity
Source: Antibiotics (Basel). 2022 Jul 30;11(8):1026. doi: 10.3390/antibiotics11081026 (PMC9405029; doi:10.3390/antibiotics11081026)
Supplement: Supplementary file 1 [file antibiotics-11-01026-s001.zip › antibiotics-1833458-supplementary.pdf]

Supplementary Material

Original ^{1}H, ^{13}C NMR and ESI-HRMS spectra of target compounds (FA-1 ~ FA-26)

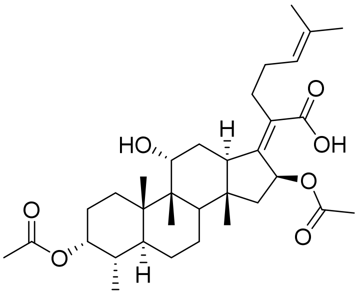

Structure of FA-1

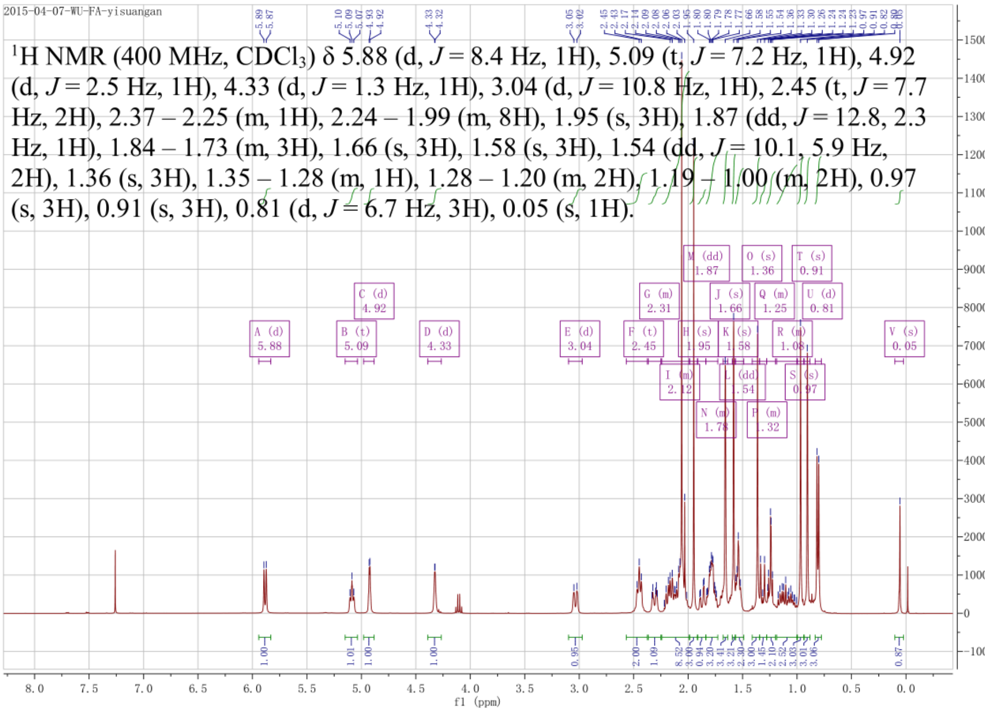

^{1}H NMR of compound FA-1

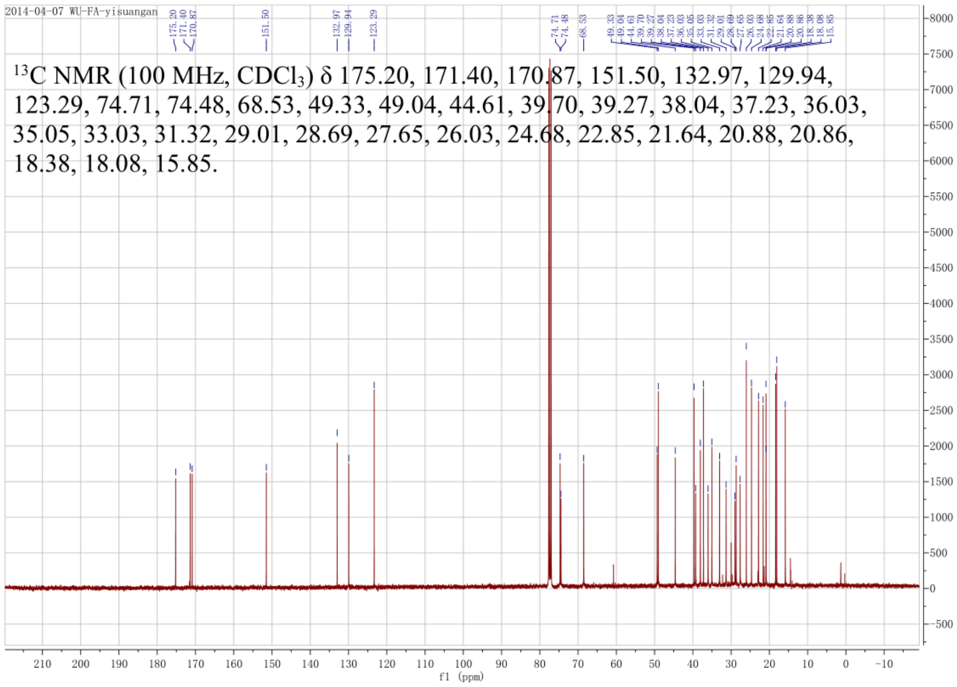

^{13}C NMR of compound FA-1

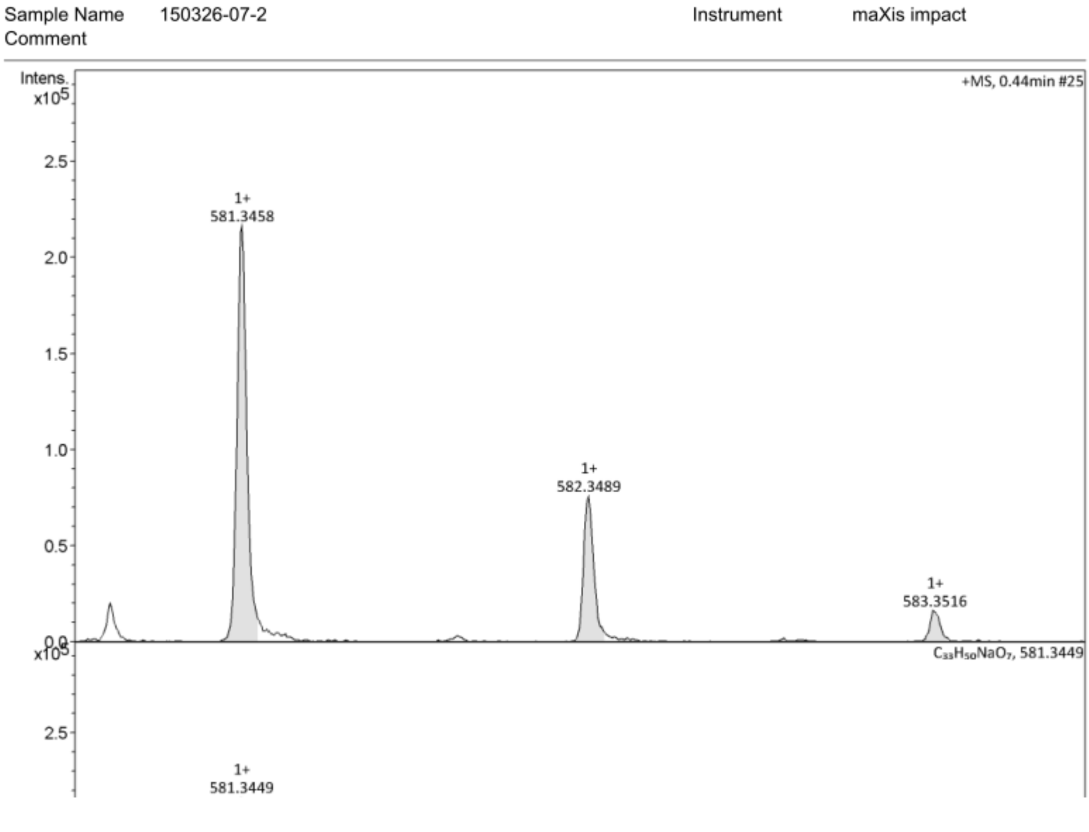

HRMS of compound FA-1

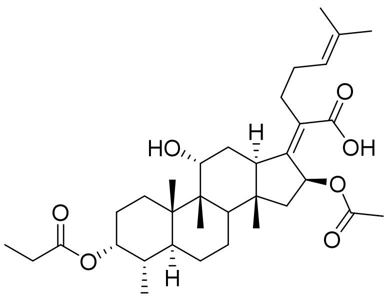

Structure of FA-2

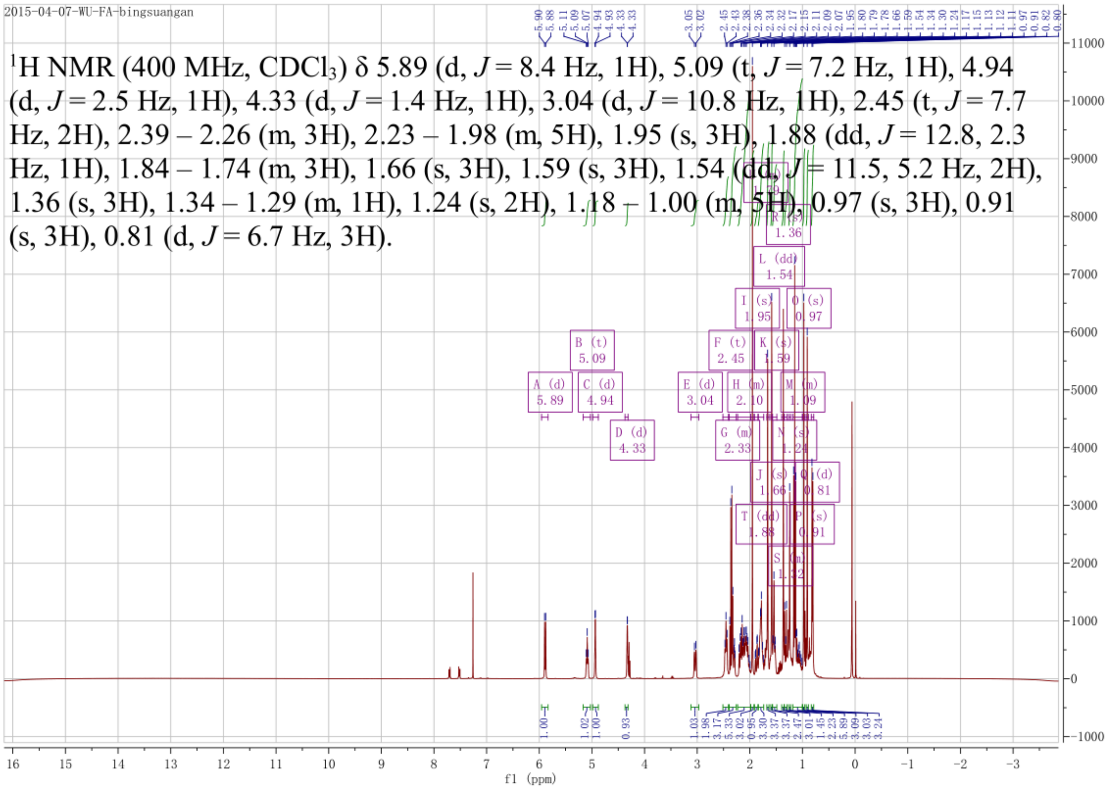

^{1}H NMR (400 MHz, $CDCl_3$) δ 5.89 (d, *J* = 8.4 Hz, 1H), 5.09 (t, *J* = 7.2 Hz, 1H), 4.94 (d, *J* = 2.5 Hz, 1H), 4.33 (d, *J* = 1.4 Hz, 1H), 3.04 (d, *J* = 10.8 Hz, 1H), 2.45 (t, *J* = 7.7 Hz, 2H), 2.39 – 2.26 (m, 3H), 2.23 – 1.98 (m, 5H), 1.95 (s, 3H), 1.88 (dd, *J* = 12.8, 2.3 Hz, 1H), 1.84 – 1.74 (m, 3H), 1.66 (s, 3H), 1.59 (s, 3H), 1.54 (dd, *J* = 11.5, 5.2 Hz, 2H), 1.36 (s, 3H), 1.34 – 1.29 (m, 1H), 1.24 (s, 2H), 1.18 – 1.00 (m, 5H), 0.97 (s, 3H), 0.91 (s, 3H), 0.81 (d, *J* = 6.7 Hz, 3H).

^{1}H NMR of compound FA-2

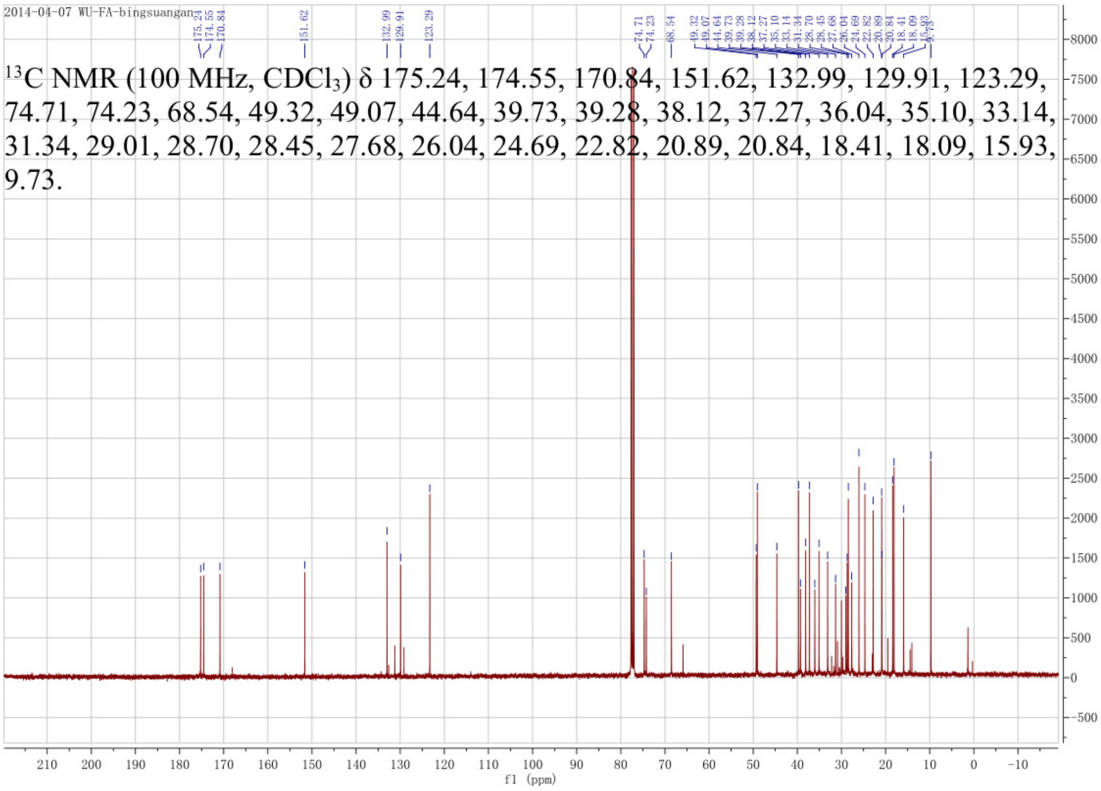

^{13}C NMR of compound FA-2

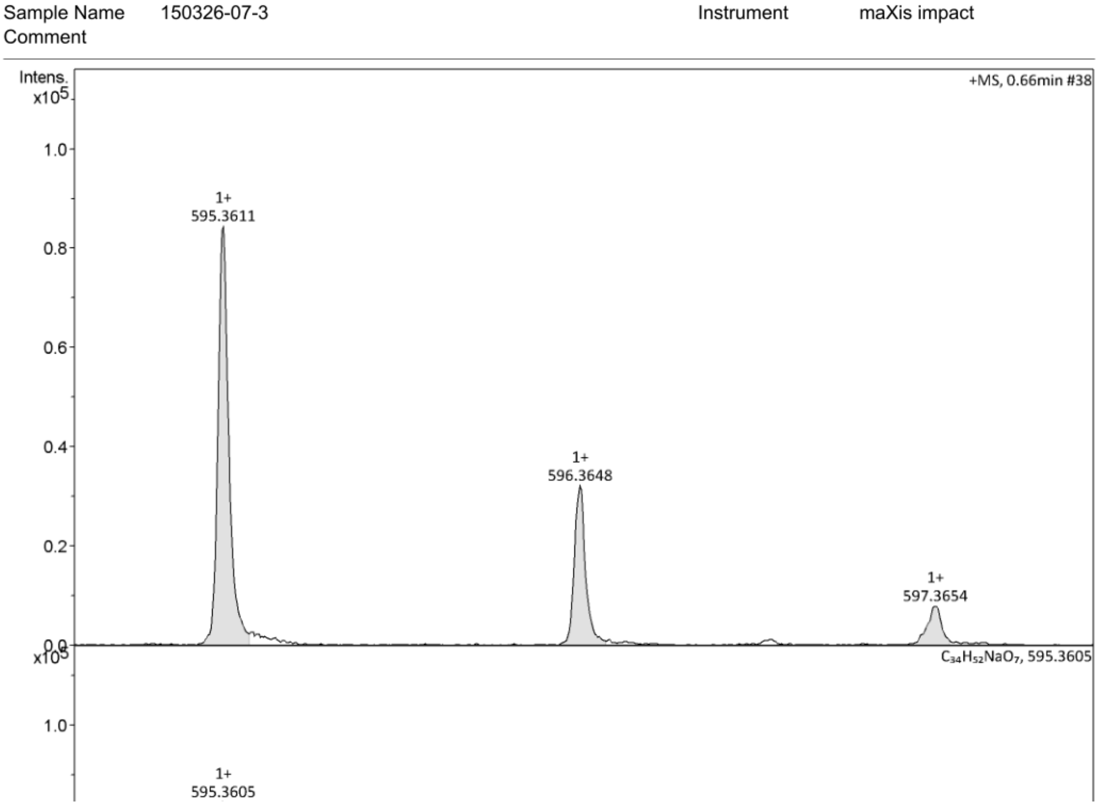

HRMS of compound FA-2

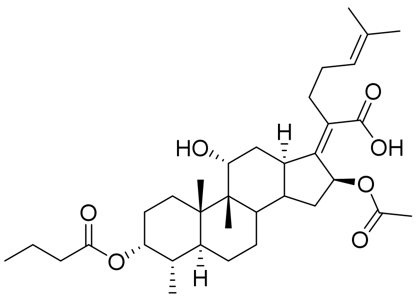

Structure of FA-3

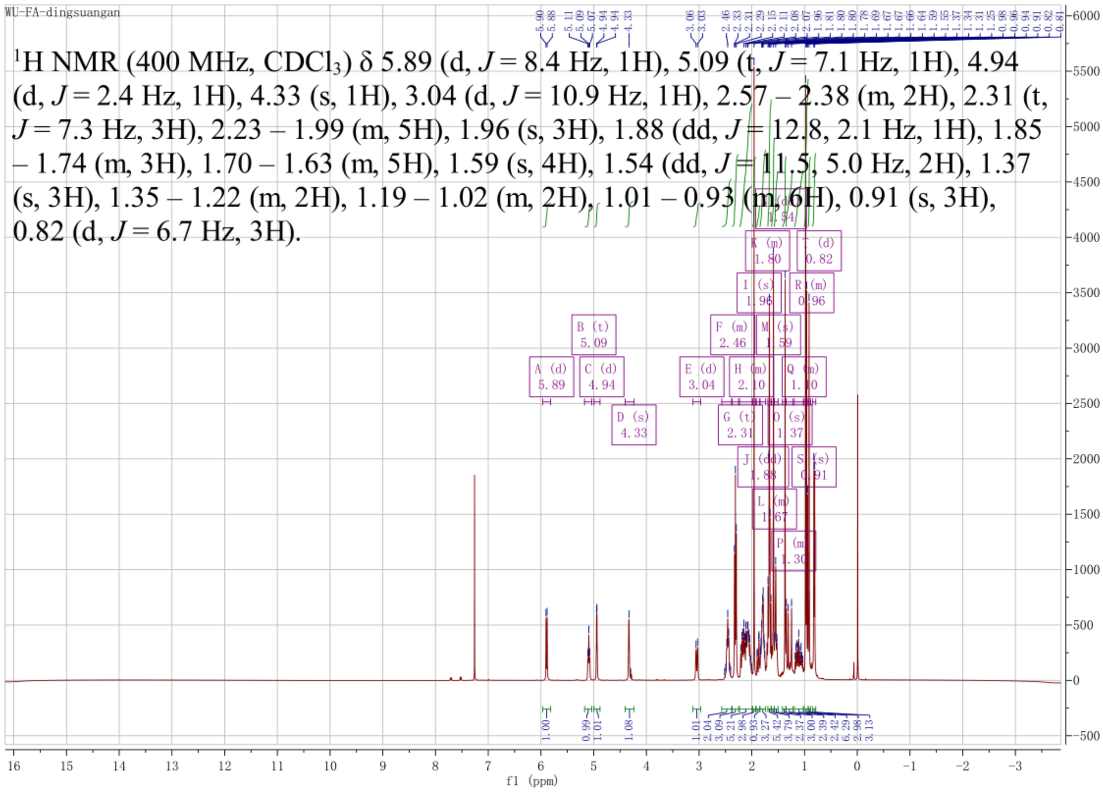

^{1}H NMR (400 MHz, $CDCl_3$) δ 5.89 (d, J = 8.4 Hz, 1H), 5.09 (t, J = 7.1 Hz, 1H), 4.94 (d, J = 2.4 Hz, 1H), 4.33 (s, 1H), 3.04 (d, J = 10.9 Hz, 1H), 2.57 – 2.38 (m, 2H), 2.31 (t, J = 7.3 Hz, 3H), 2.23 – 1.99 (m, 5H), 1.96 (s, 3H), 1.88 (dd, J = 12.8, 2.1 Hz, 1H), 1.85 – 1.74 (m, 3H), 1.70 – 1.63 (m, 5H), 1.59 (s, 4H), 1.54 (dd, J = 11.5, 5.0 Hz, 2H), 1.37 (s, 3H), 1.35 – 1.22 (m, 2H), 1.19 – 1.02 (m, 2H), 1.01 – 0.93 (m, 6H), 0.91 (s, 3H), 0.82 (d, J = 6.7 Hz, 3H).

^{1}H NMR of compound FA-3

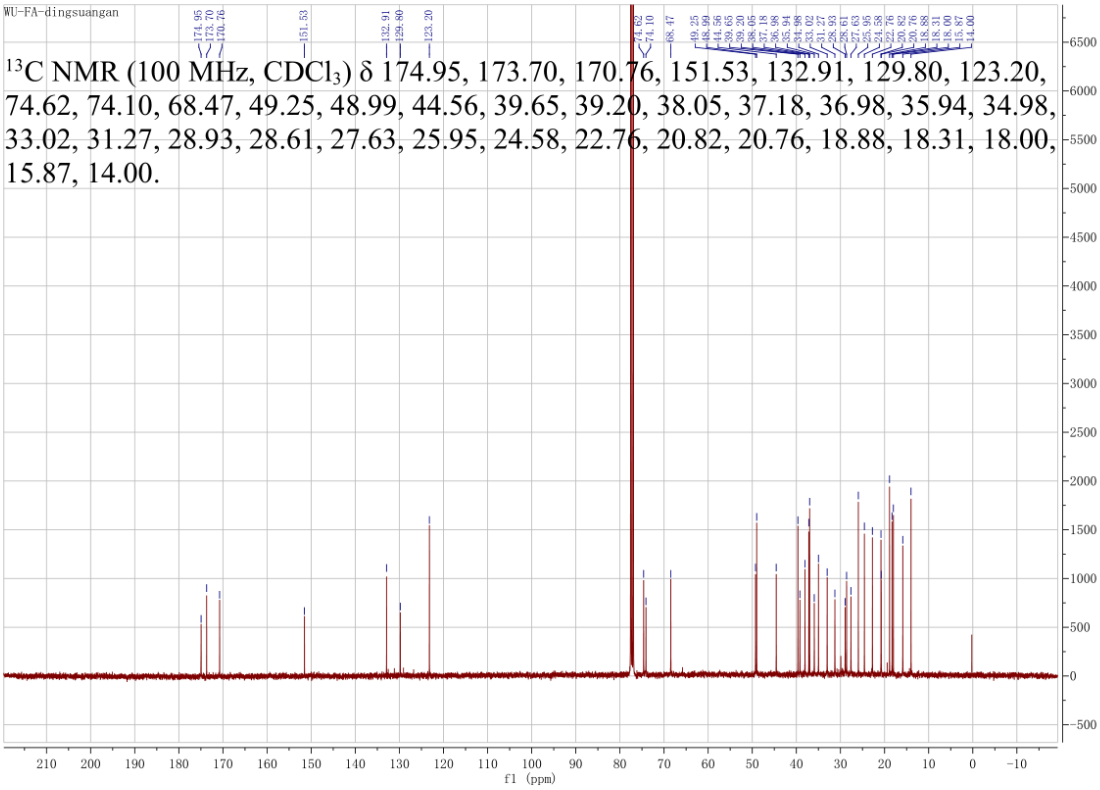

^{13}C NMR of compound FA-3

Sample Name 150326-07-4 Instrument maXis impact
Comment

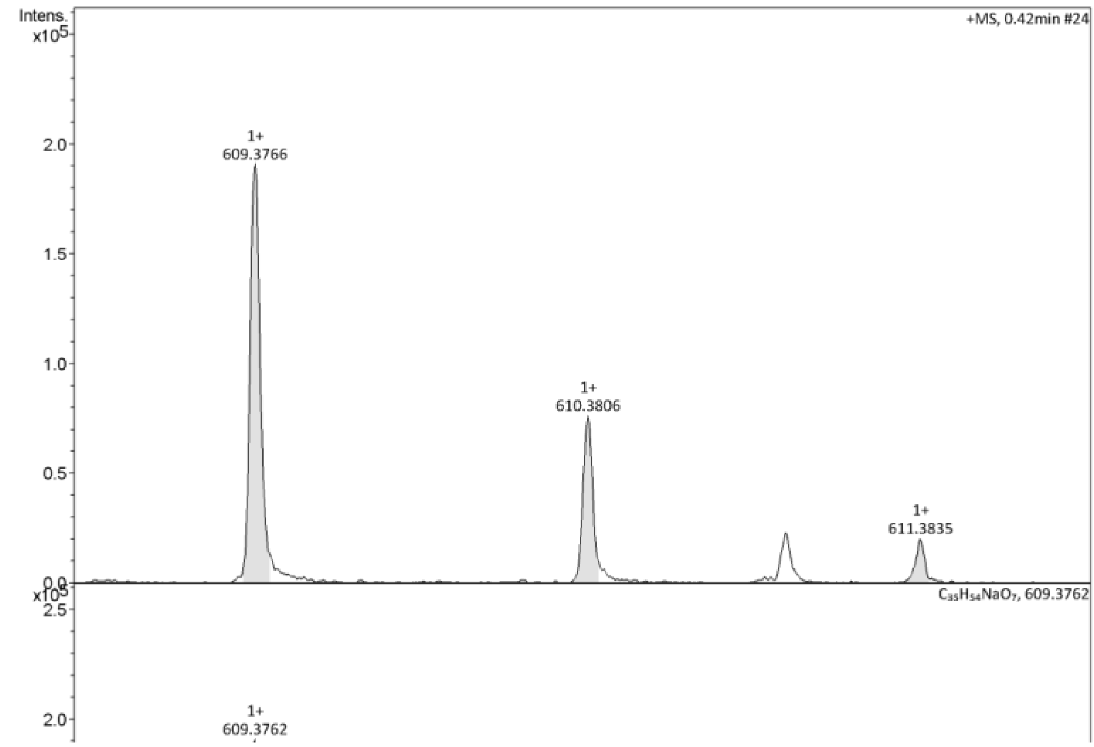

HRMS of compound FA-3

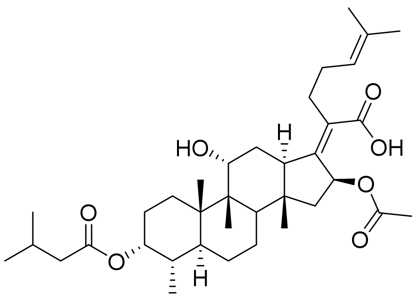

Structure of FA-4

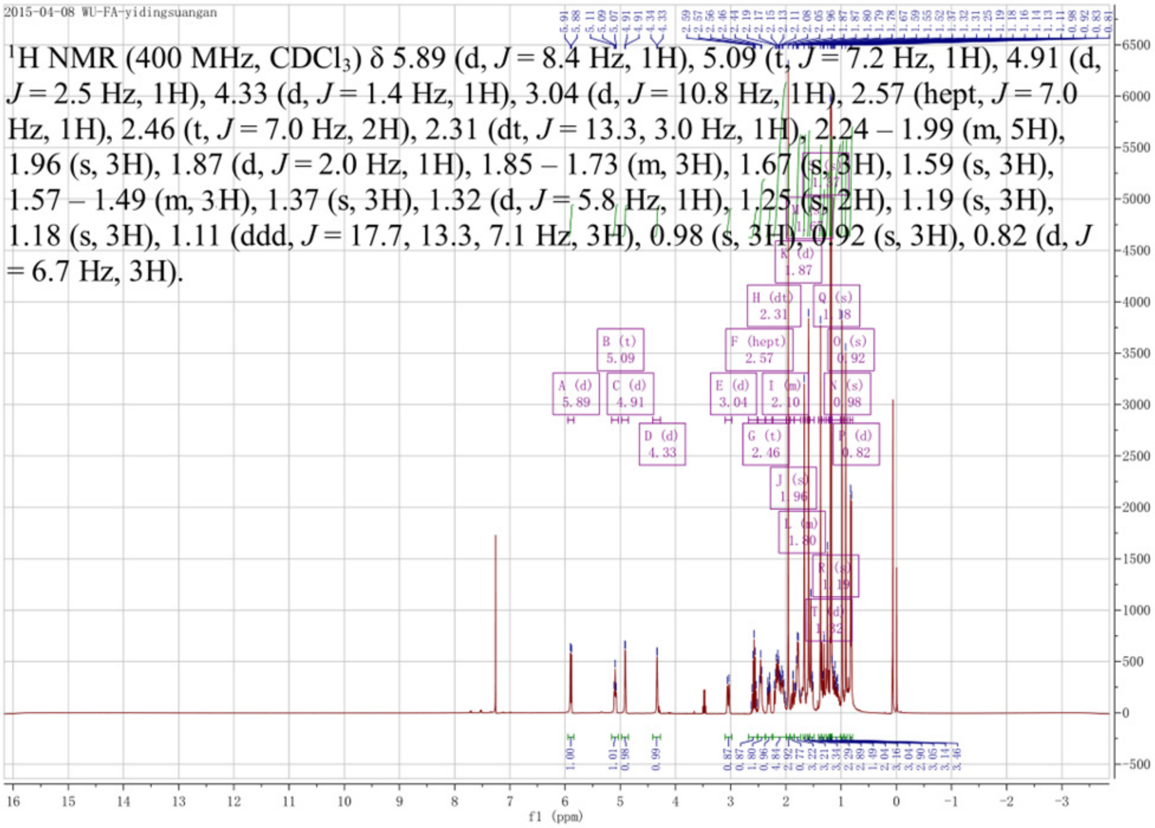

^{1}H NMR of compound FA-4

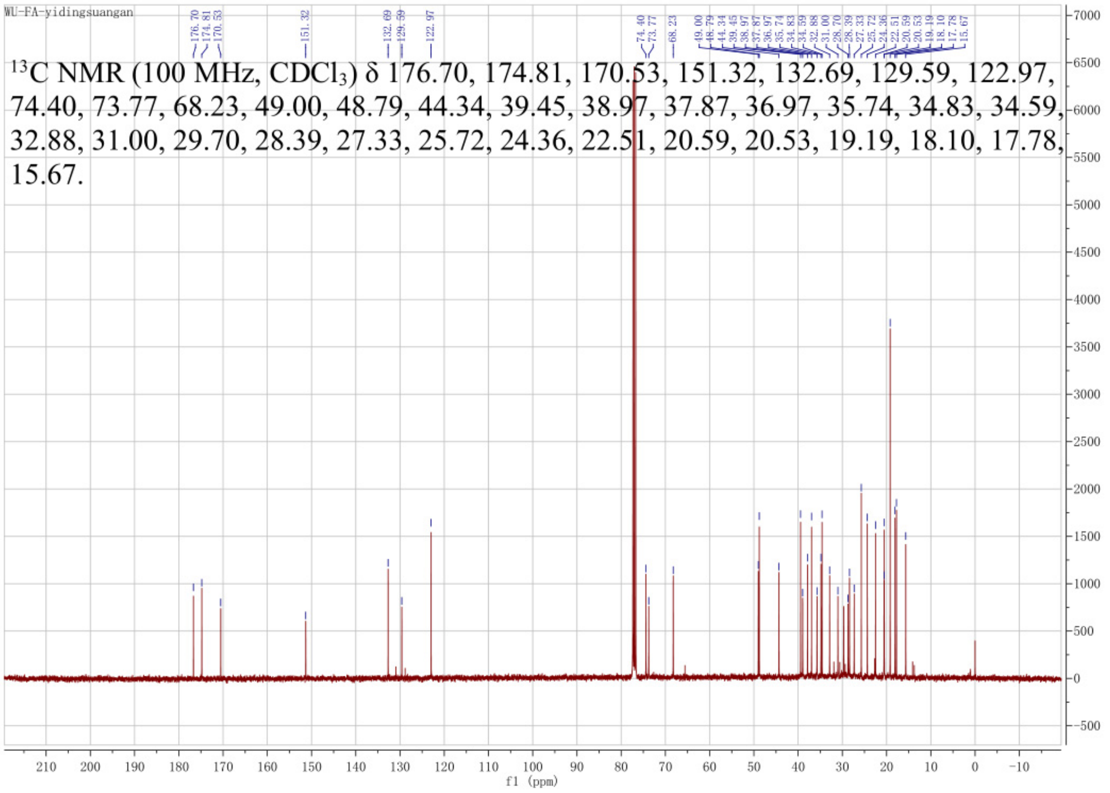

^{13}C NMR of compound FA-4

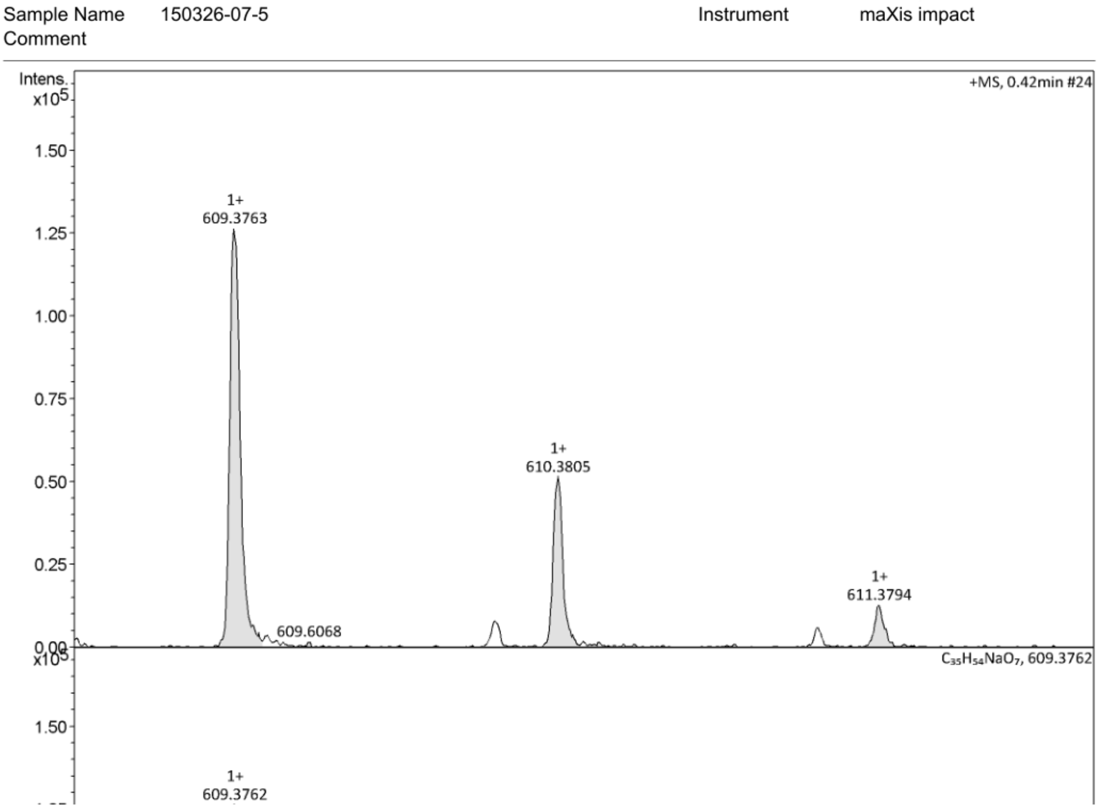

HRMS of compound FA-4

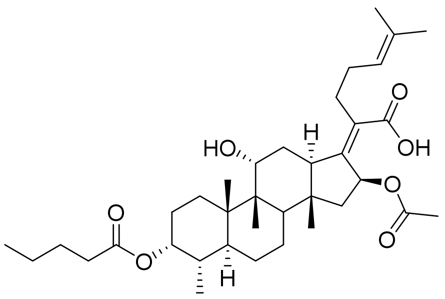

Structure of FA-5

^{1}H NMR (400 MHz, $CDCl_3$) δ 5.87 (d, *J* = 8.4 Hz, 1H), 5.07 (t, *J* = 7.2 Hz, 1H), 4.31 (s, 1H), 3.03 (d, *J* = 11.1 Hz, 1H), 2.43 (t, *J* = 7.5 Hz, 2H), 2.36 – 2.25 (m, 3H), 2.20 – 1.99 (m, *J* = 28.6, 14.6, 8.0 Hz, 5H), 1.93 (s, 3H), 1.89 – 1.72 (m, 4H), 1.64 (s, 3H), 1.62 – 1.47 (m, 8H), 1.35 (s, 2H), 1.35 – 1.26 (m, 4H), 1.26 – 1.21 (m, *J* = 11.8 Hz, 2H), 1.14 – 1.02 (m, *J* = 17.9, 13.1, 7.0 Hz, 2H), 0.96 (s, 3H), 0.89 (t, *J* = 7.3 Hz, 6H), 0.79 (d, *J* = 6.7 Hz, 3H).

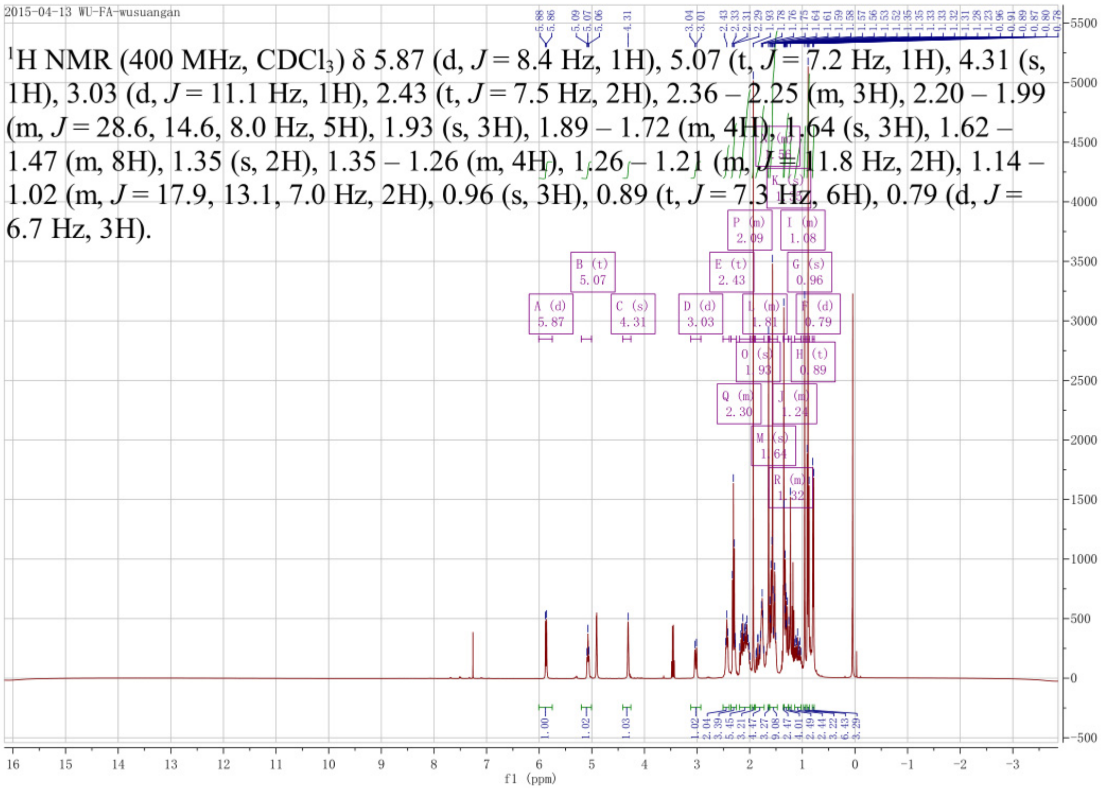

^{1}H NMR of compound FA-5

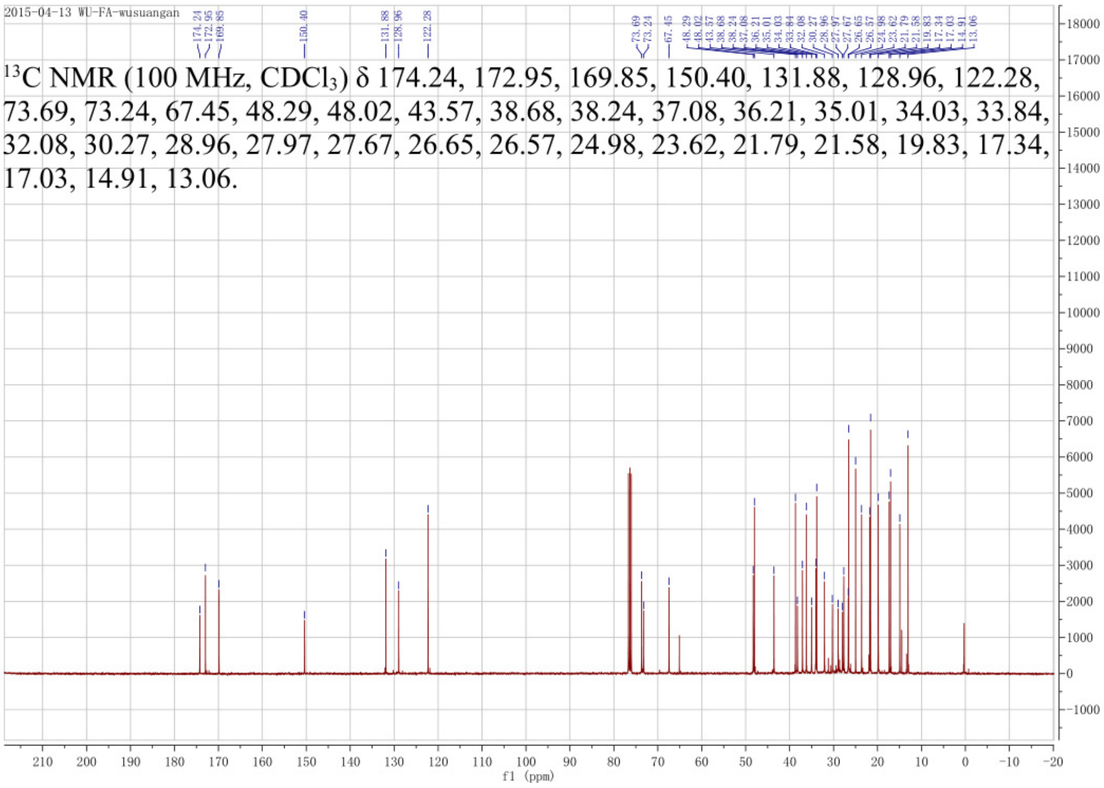

^{13}C NMR of compound FA-5

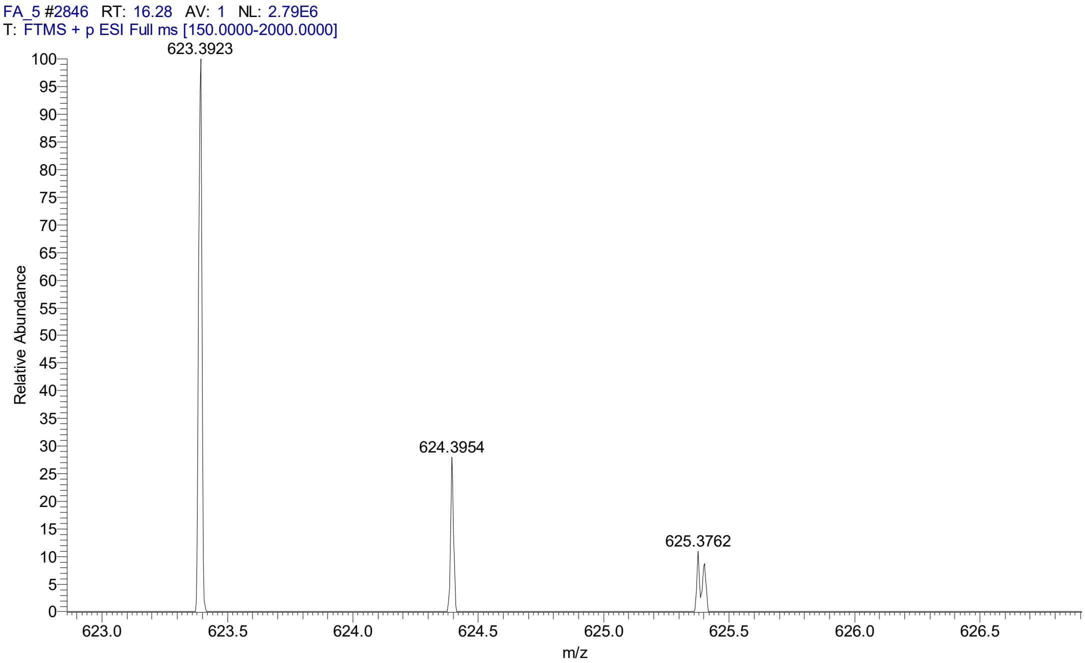

HRMS of compound FA-5

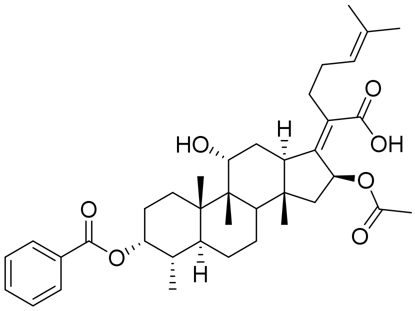

Structure of FA-6

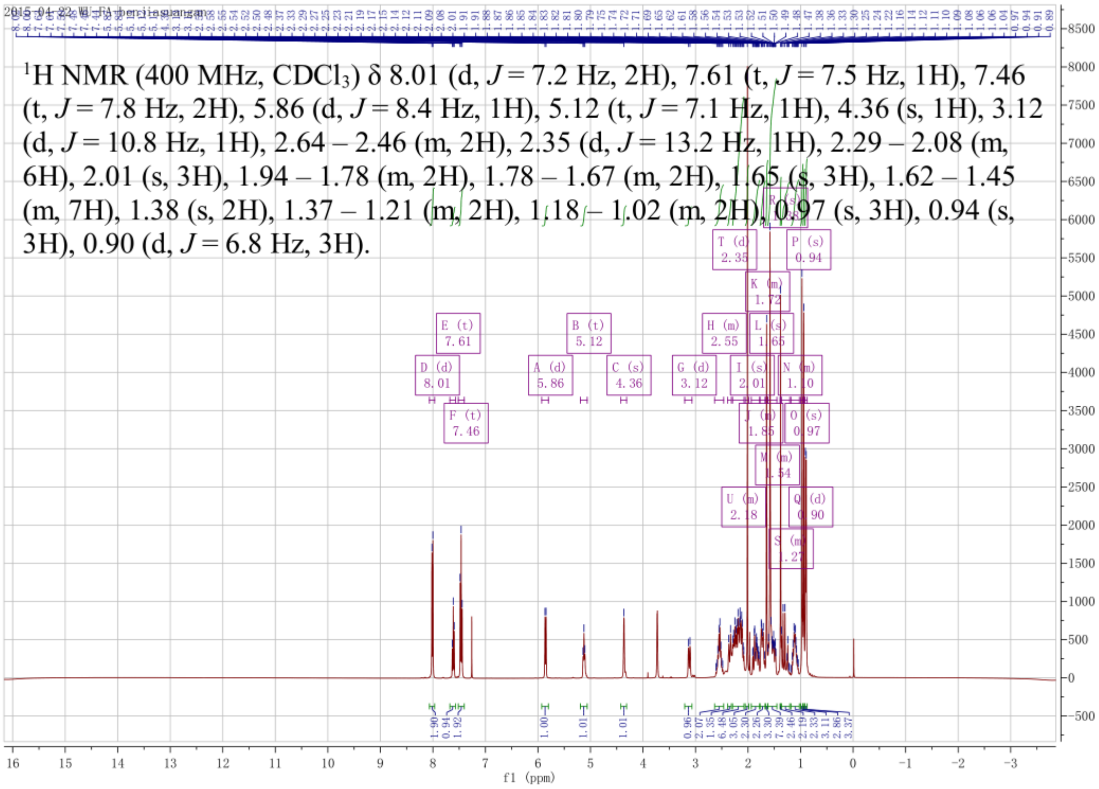

^{1}H NMR of compound FA-6

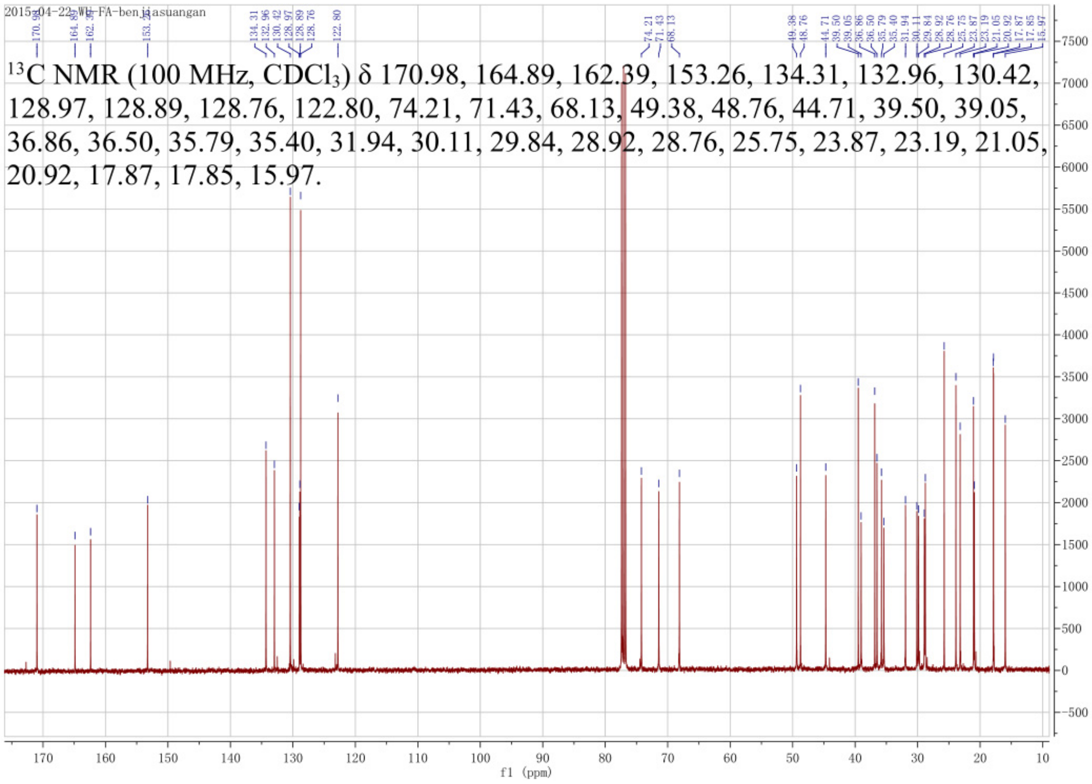

^{13}C NMR of compound FA-6

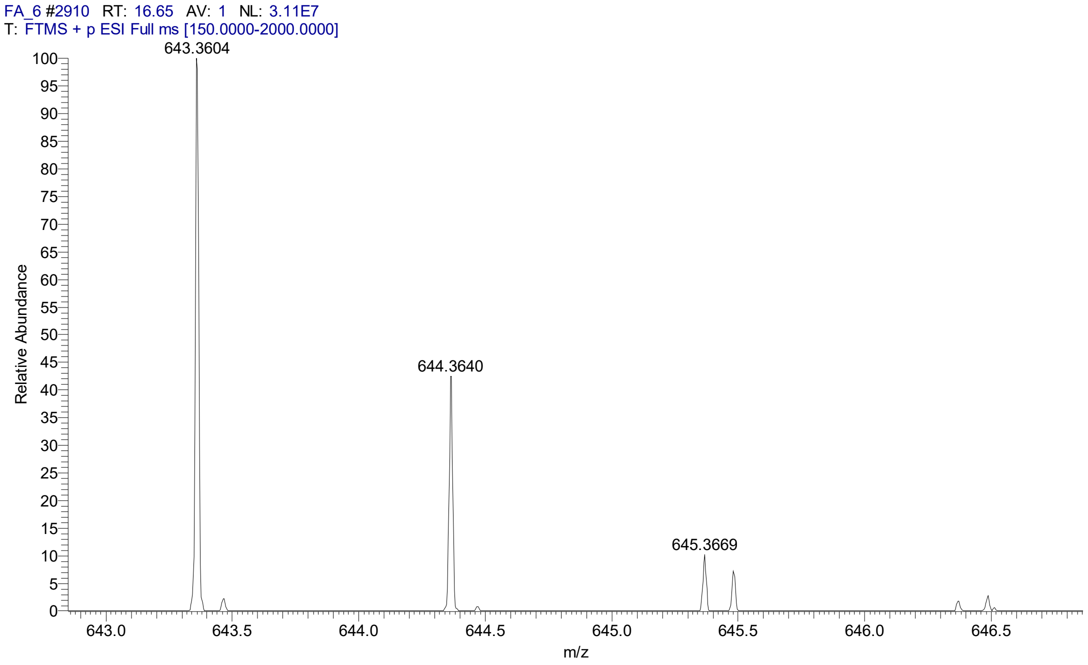

HRMS of compound FA-6

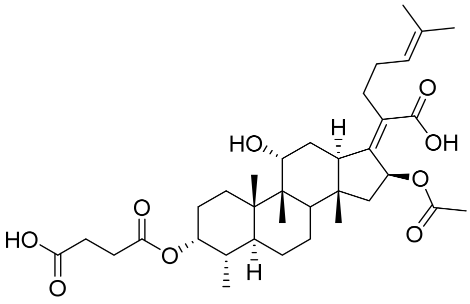

Structure of FA-7

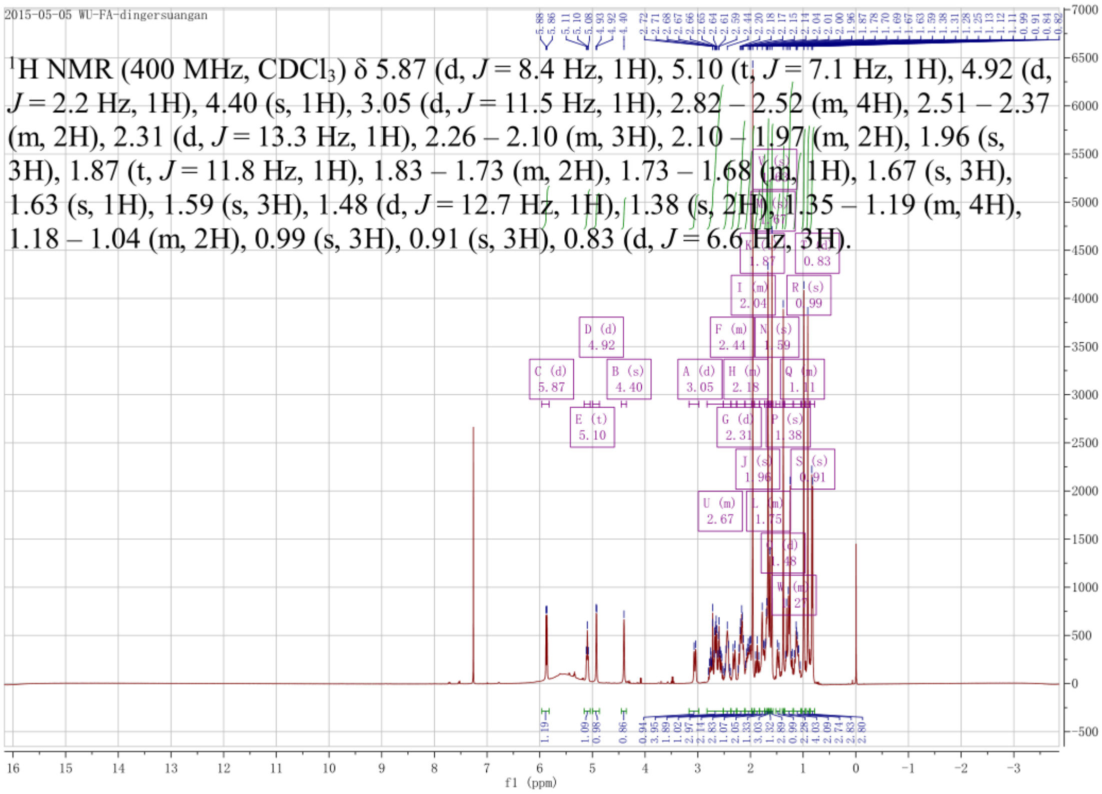

^{1}H NMR (400 MHz, $CDCl_3$) δ 5.87 (d, J = 8.4 Hz, 1H), 5.10 (t, J = 7.1 Hz, 1H), 4.92 (d, J = 2.2 Hz, 1H), 4.40 (s, 1H), 3.05 (d, J = 11.5 Hz, 1H), 2.82 – 2.52 (m, 4H), 2.51 – 2.37 (m, 2H), 2.31 (d, J = 13.3 Hz, 1H), 2.26 – 2.10 (m, 3H), 2.10 – 1.97 (m, 2H), 1.96 (s, 3H), 1.87 (t, J = 11.8 Hz, 1H), 1.83 – 1.73 (m, 2H), 1.73 – 1.68 (m, 1H), 1.67 (s, 3H), 1.63 (s, 1H), 1.59 (s, 3H), 1.48 (d, J = 12.7 Hz, 1H), 1.38 (s, 2H), 1.35 – 1.19 (m, 4H), 1.18 – 1.04 (m, 2H), 0.99 (s, 3H), 0.91 (s, 3H), 0.83 (d, J = 6.6 Hz, 3H).

^{1}H NMR of compound FA-7

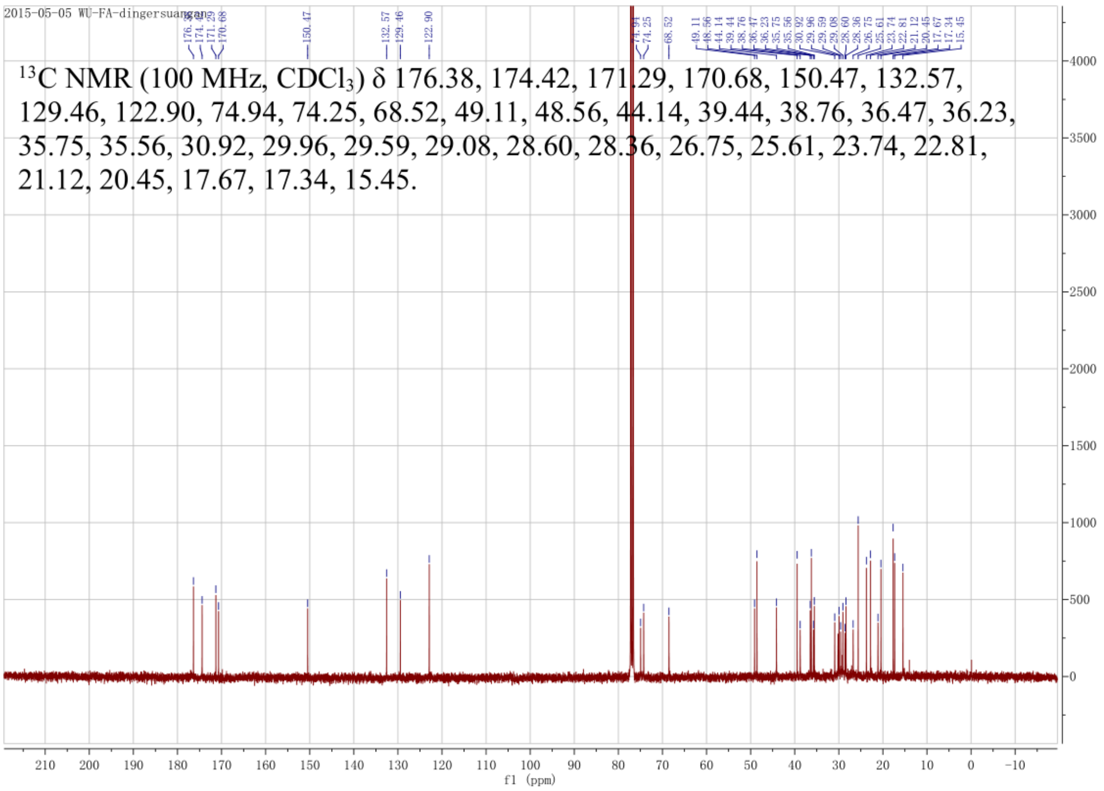

^{13}C NMR of compound FA-7

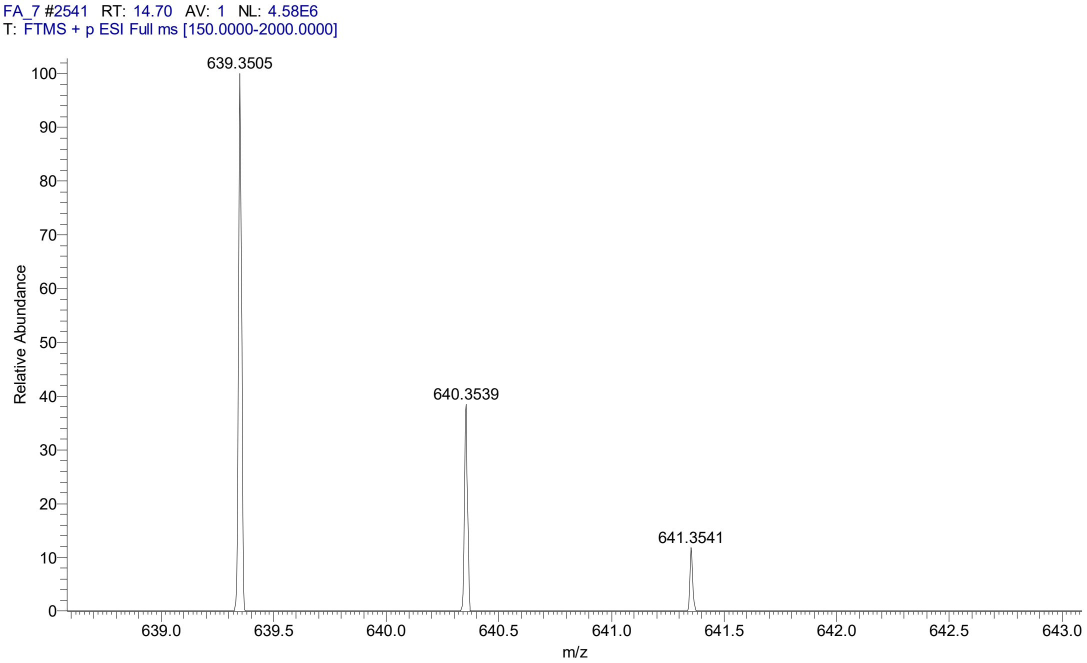

HRMS of compound FA-7

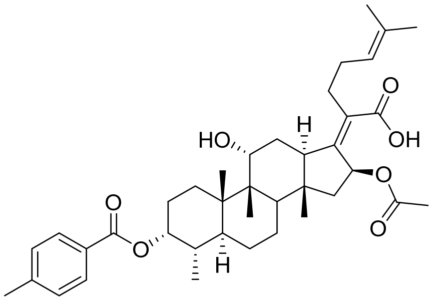

Structure of FA-8

^{1}H NMR (400 MHz, $CDCl_3$) δ 7.89 (d, *J* = 8.2 Hz, 2H), 7.25 (d, *J* = 6.6 Hz, 2H), 5.85 (d, *J* = 8.4 Hz, 1H), 5.12 (t, *J* = 7.0 Hz, 1H), 4.36 (s, 1H), 3.73 (d, *J* = 2.0 Hz, 1H), 3.11 (d, *J* = 11.0 Hz, 1H), 2.63 – 2.46 (m, 2H), 2.41 (s, 3H), 2.35 (d, *J* = 13.2 Hz, 1H), 2.31 – 2.07 (m, 5H), 2.04 – 1.97 (m, 4H), 1.92 – 1.78 (m, 2H), 1.78 – 1.68 (m, 2H), 1.65 (s, 3H), 1.60 – 1.54 (m, 5H), 1.50 (d, *J* = 12.3 Hz, 1H), 1.38 (s, 3H), 1.34 – 1.24 (m, 2H), 1.17 – 1.05 (m, 2H), 0.97 (s, 3H), 0.94 (s, 3H), 0.90 (d, *J* = 6.8 Hz, 3H).

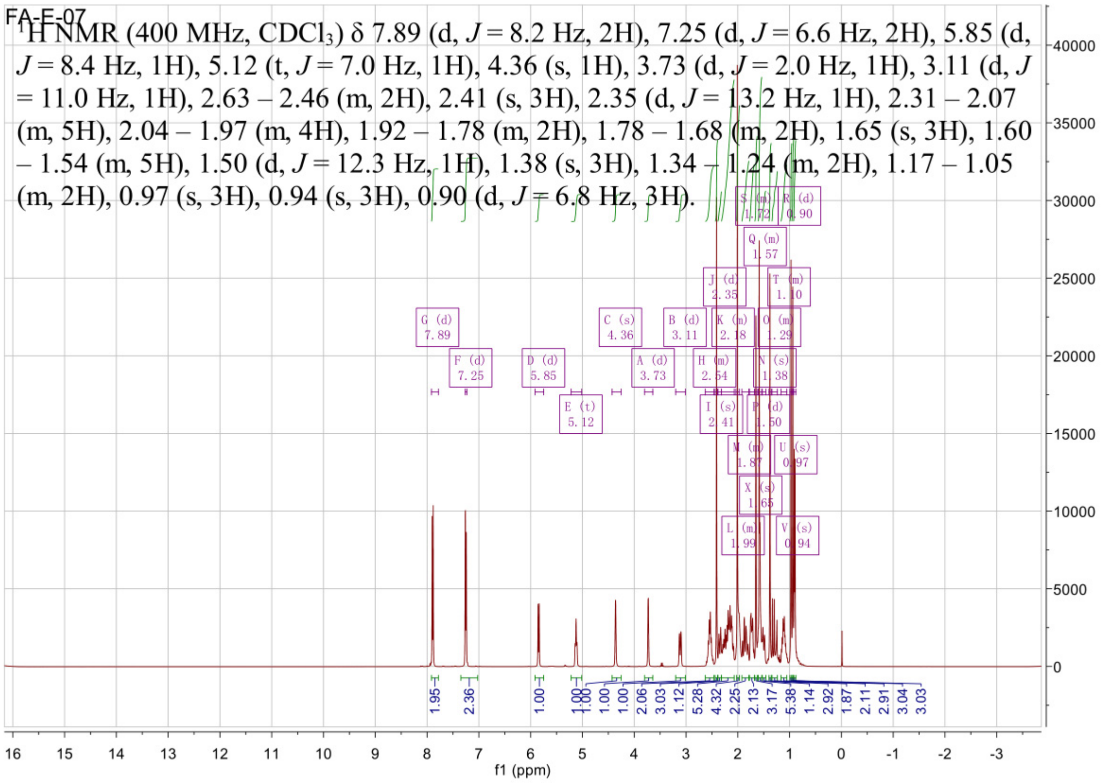

^{1}H NMR of compound FA-8

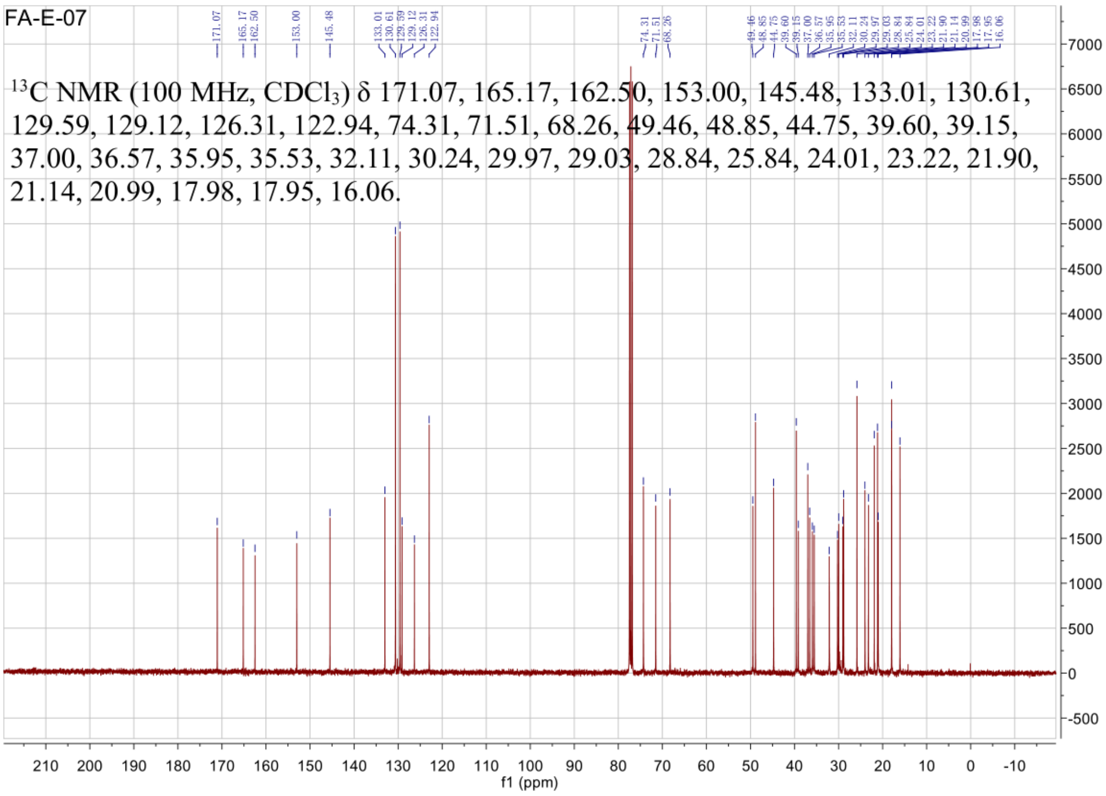

^{13}C NMR of compound FA-8

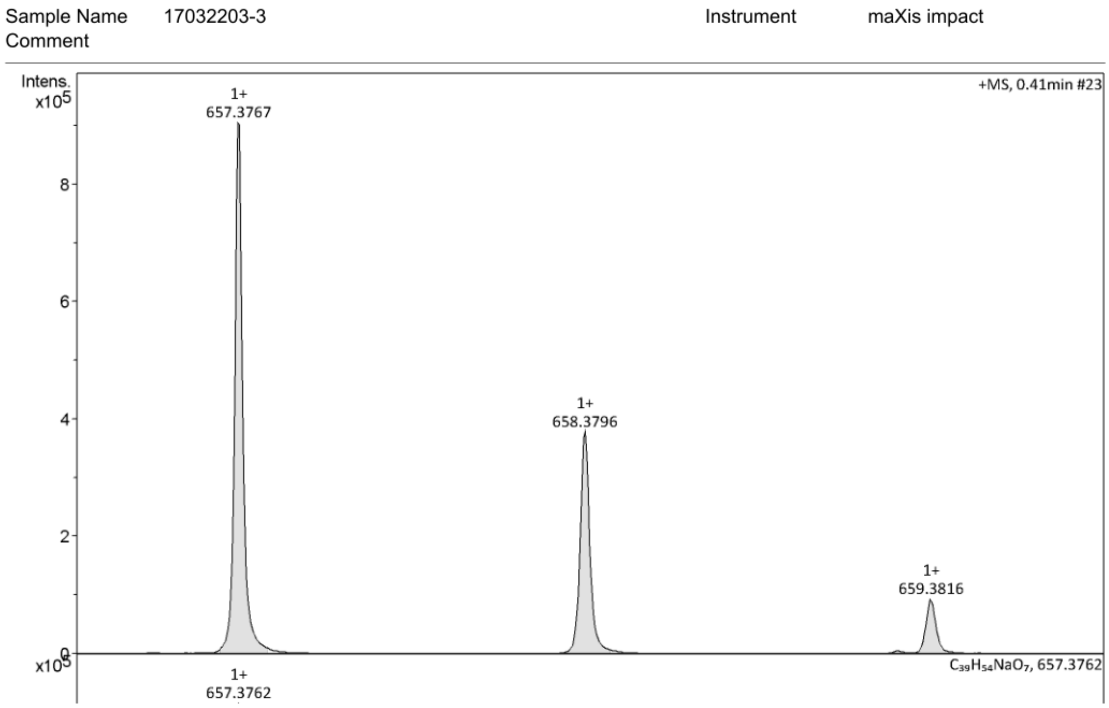

HRMS of compound FA-8

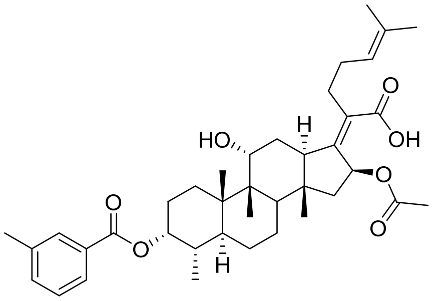

Structure of FA-9

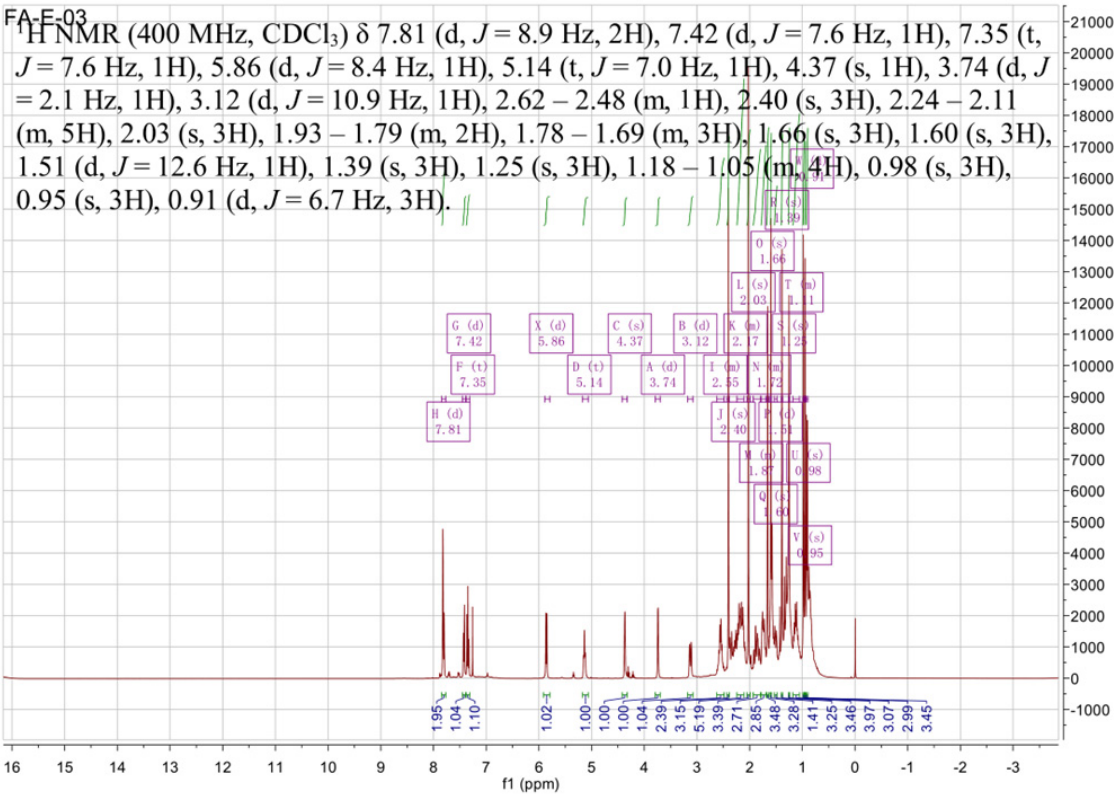

^{1}H NMR of compound FA-9

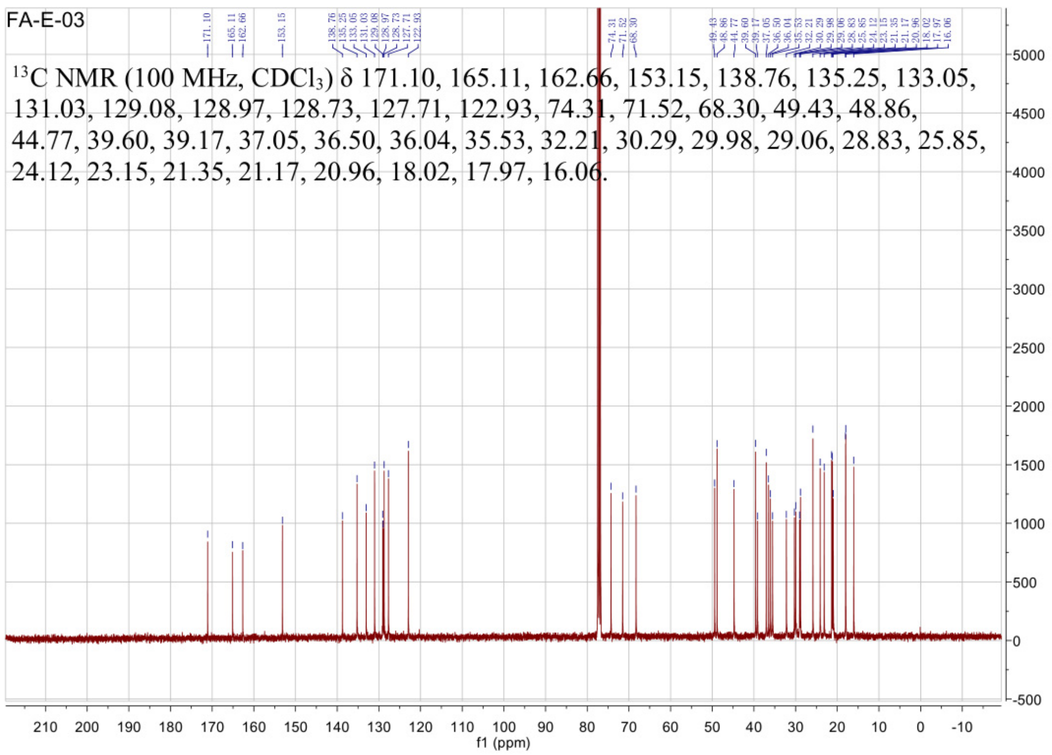

^{13}C NMR of compound FA-9

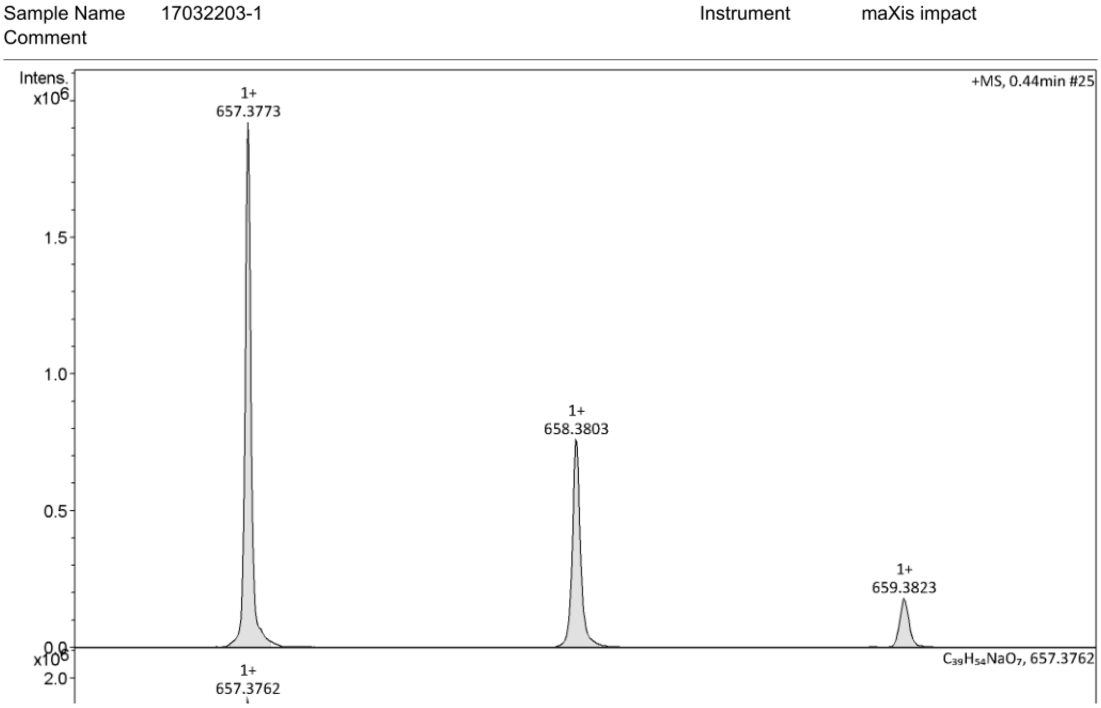

HRMS of compound FA-9

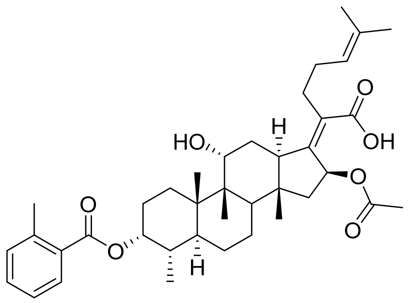

Structure of FA-10

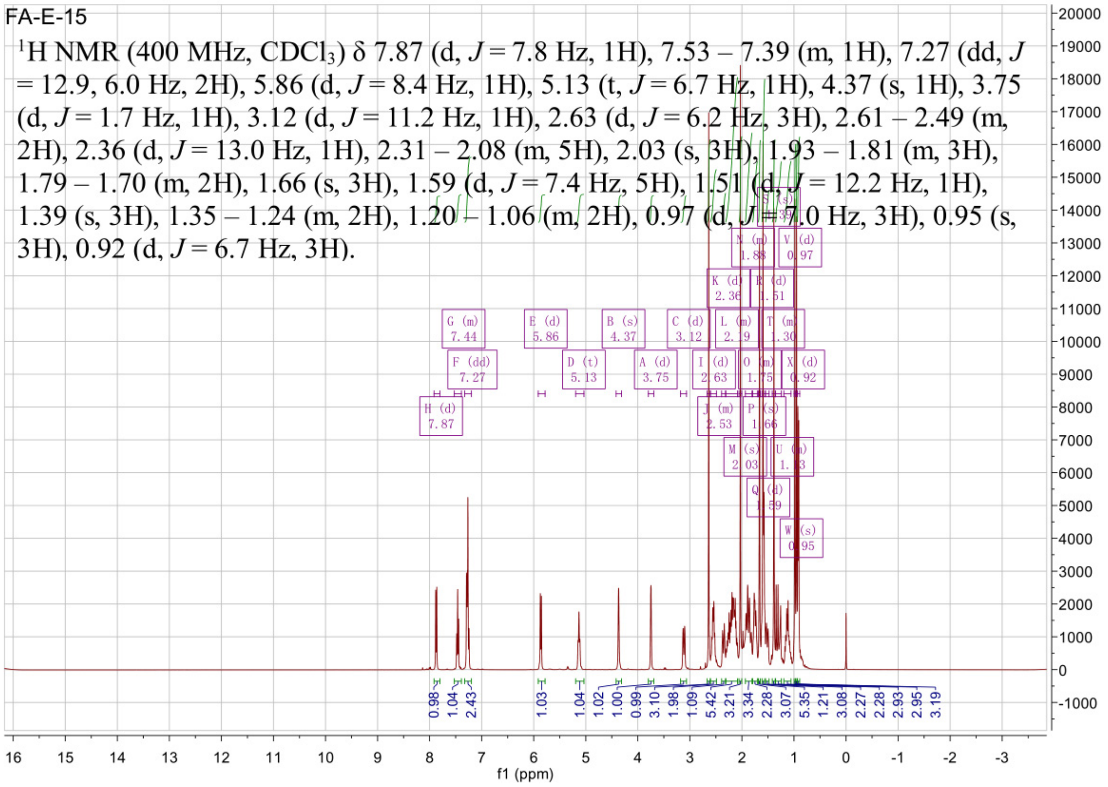

^{1}H NMR of compound FA-10

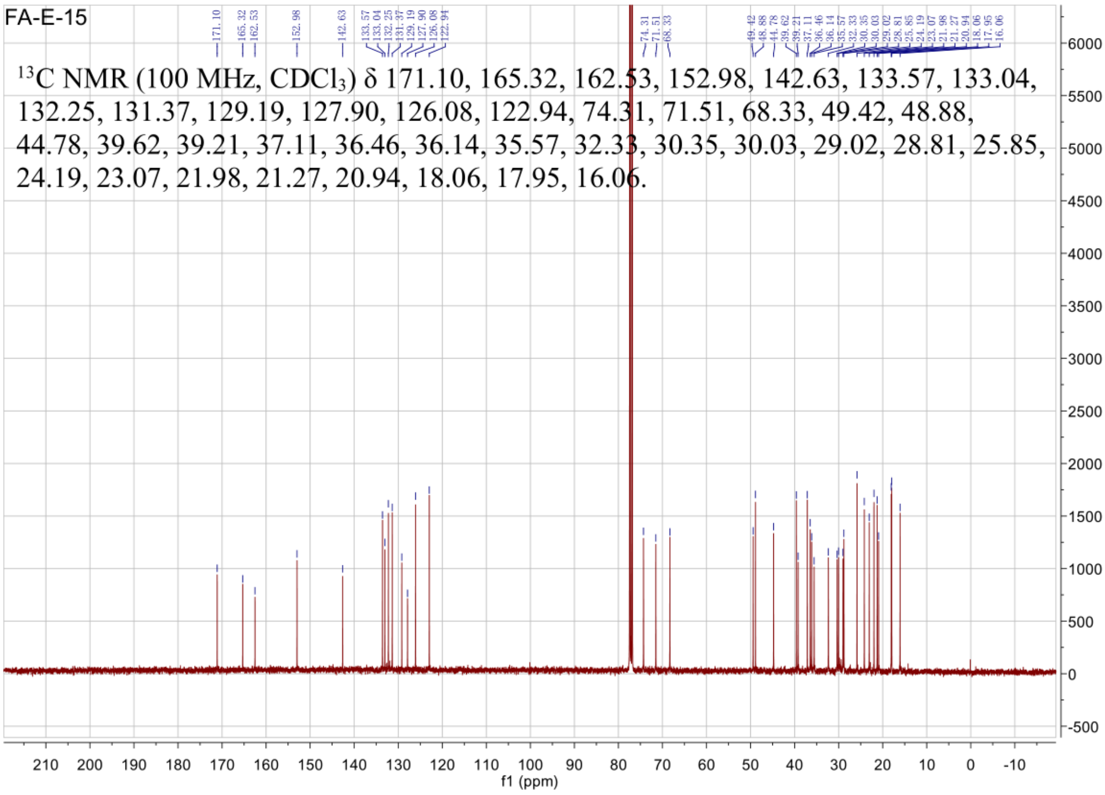

^{13}C NMR of compound FA-10

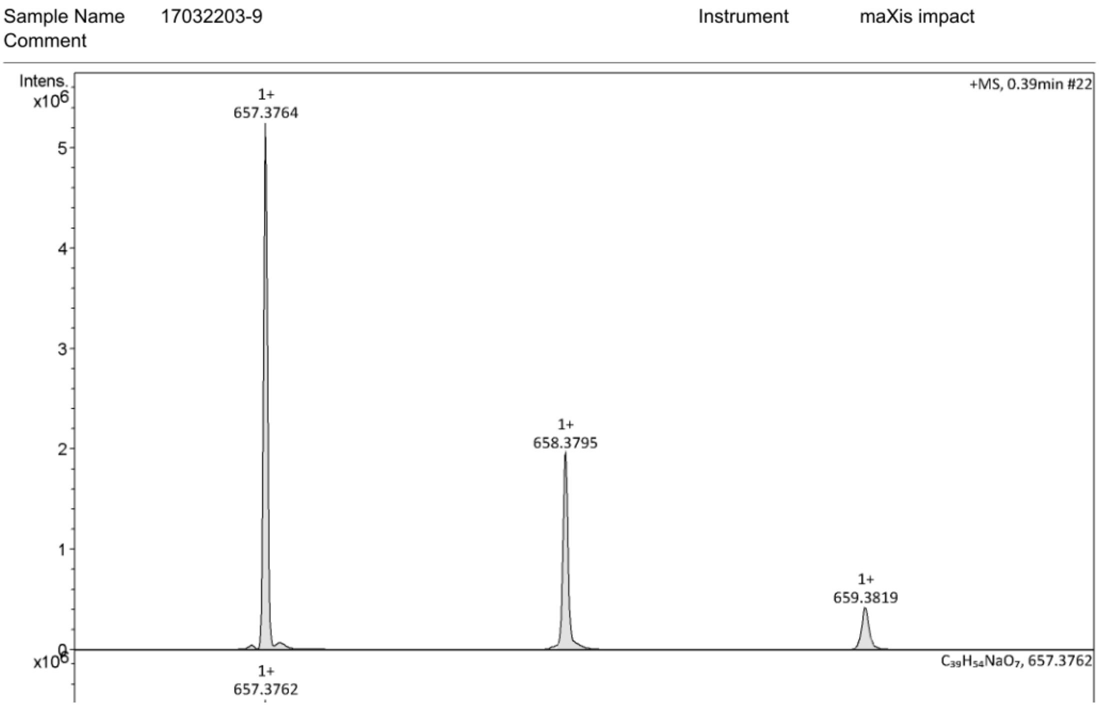

HRMS of compound FA-10

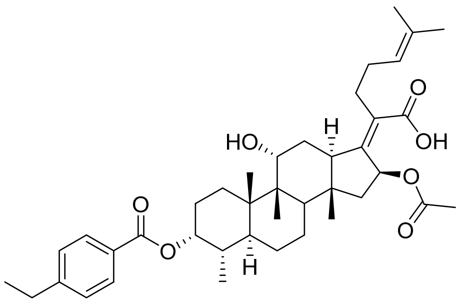

Structure of FA-11

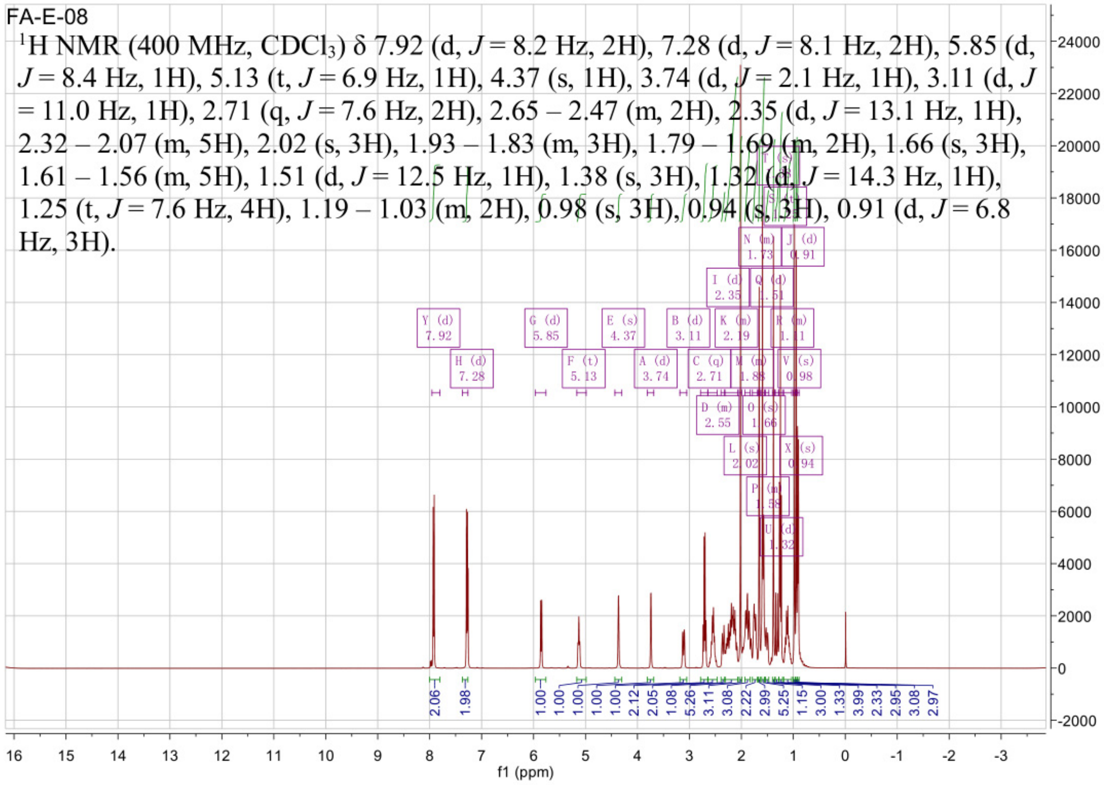

^{1}H NMR of compound FA-11

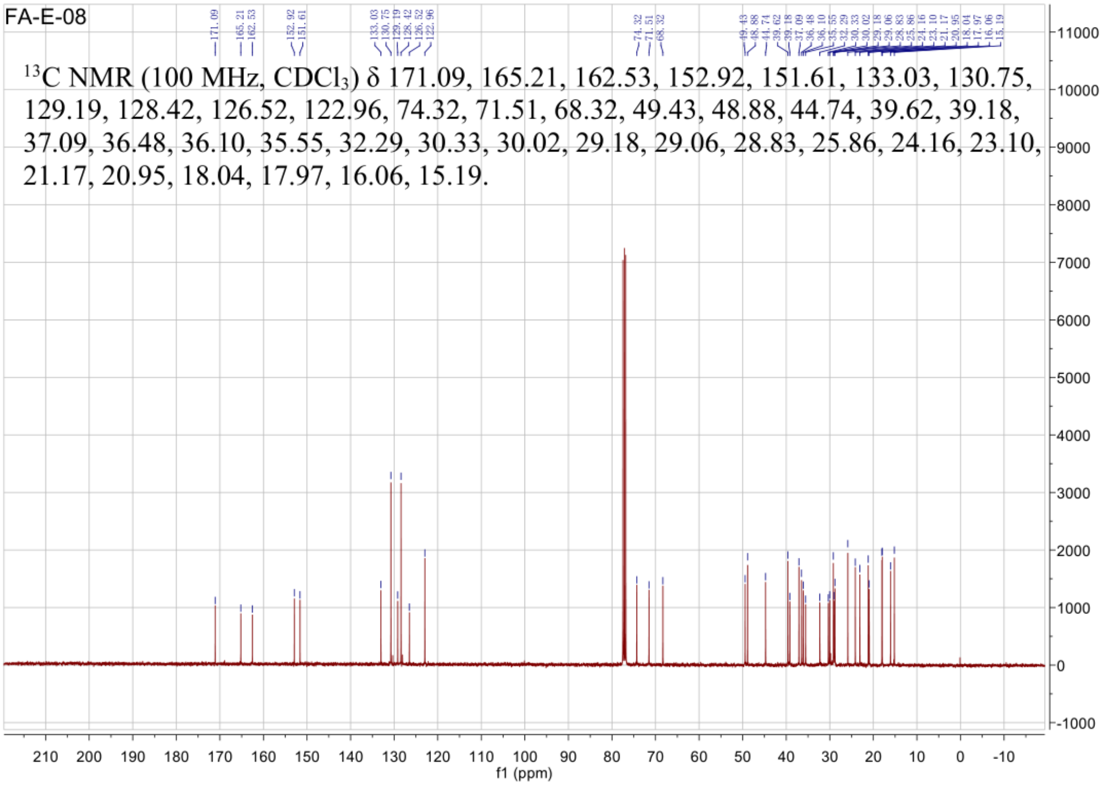

^{13}C NMR of compound FA-11

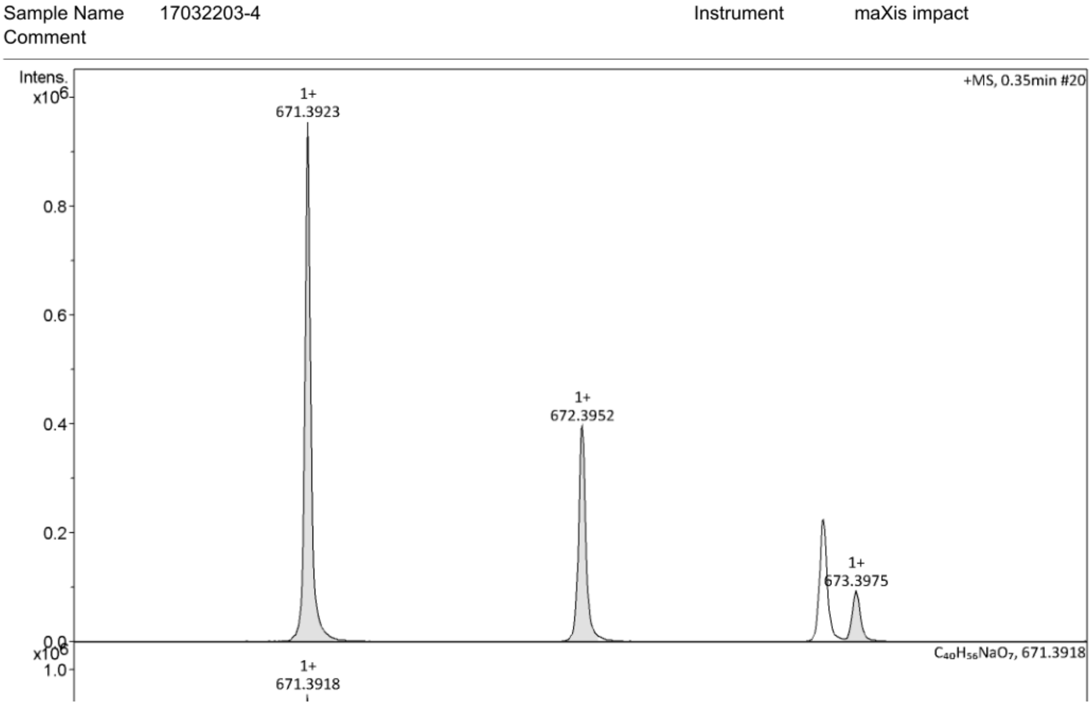

HRMS of compound FA-11

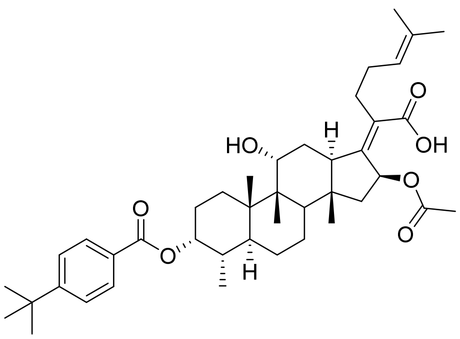

Structure of FA-12

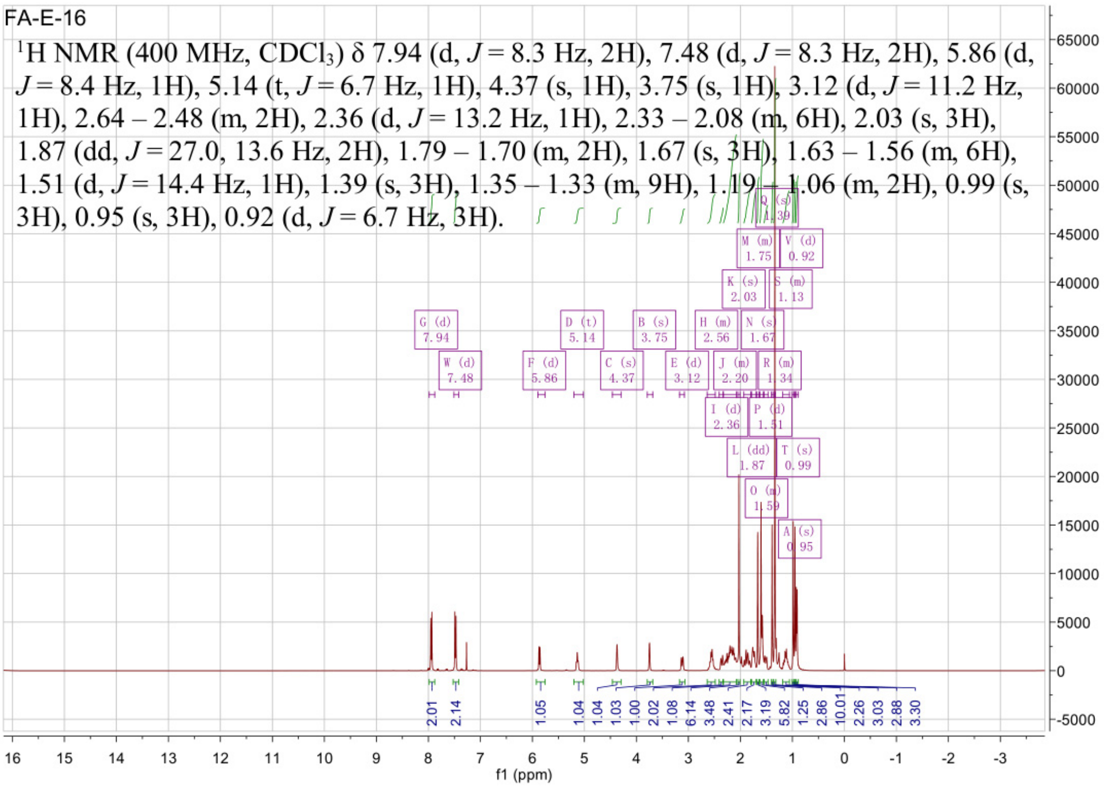

^{1}H NMR of compound FA-12

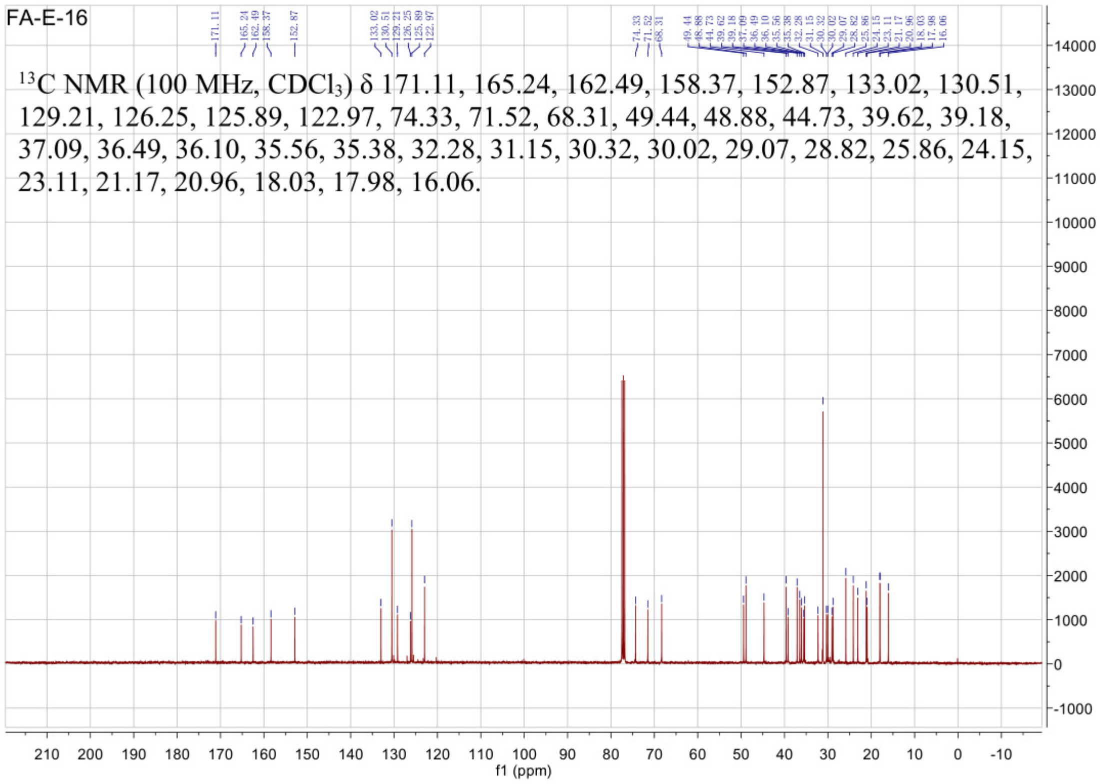

^{13}C NMR of compound FA-12

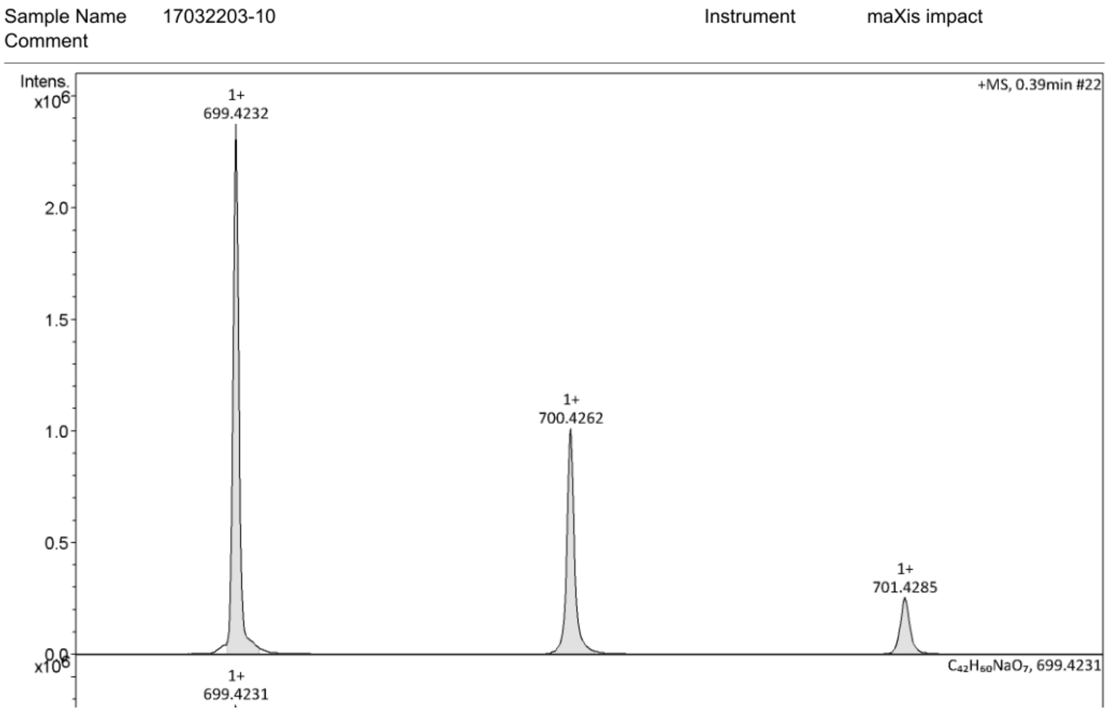

HRMS of compound FA-12

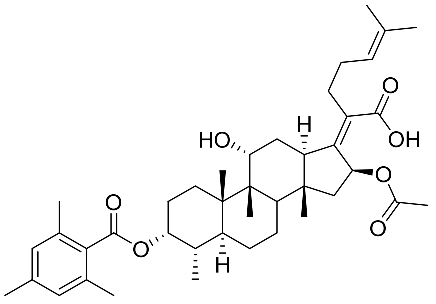

Structure of FA-13

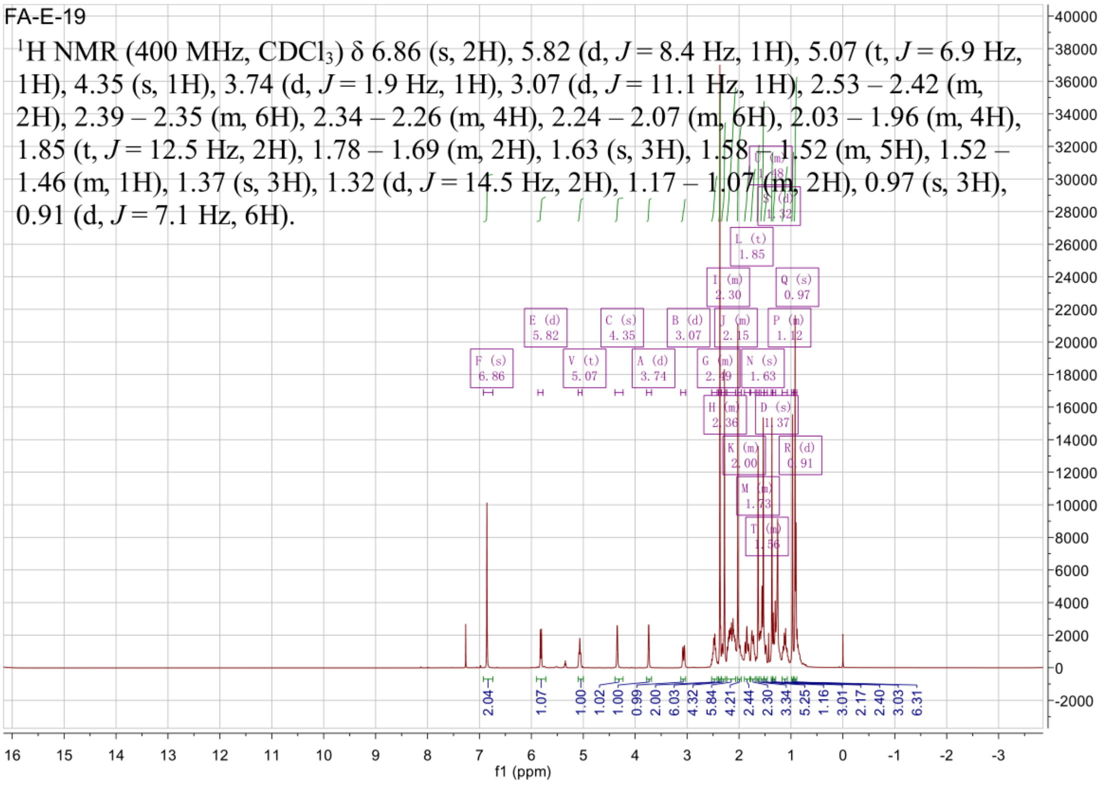

^{1}H NMR of compound FA-13

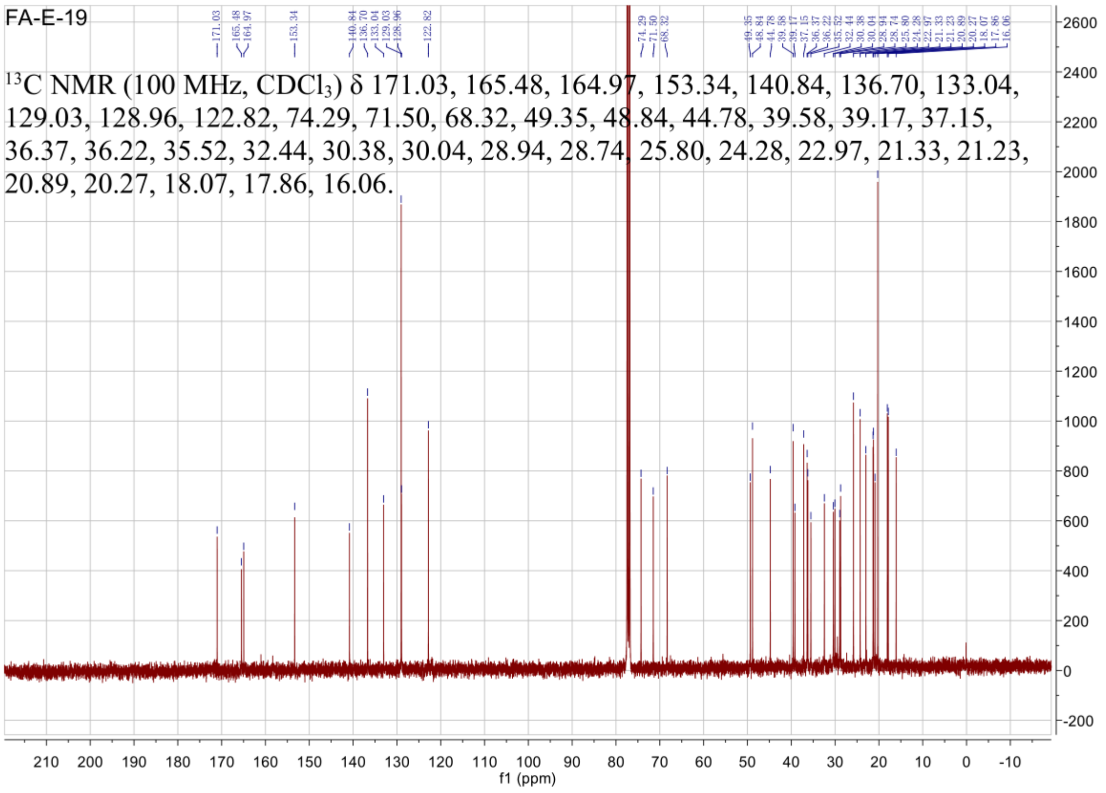

^{13}C NMR of compound FA-13

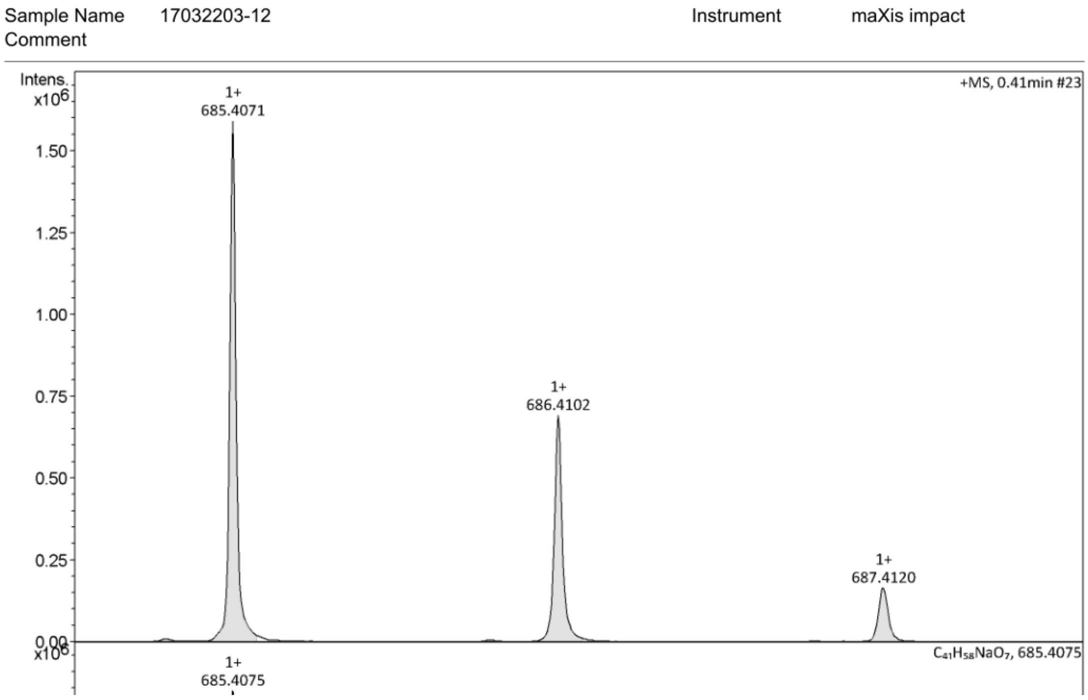

HRMS of compound FA-13

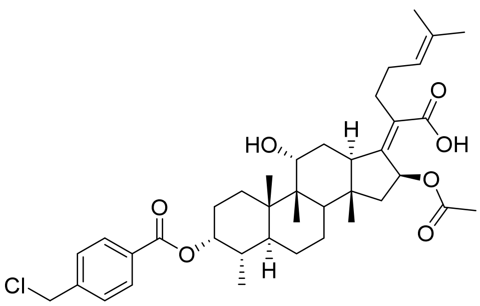

Structure of FA-14

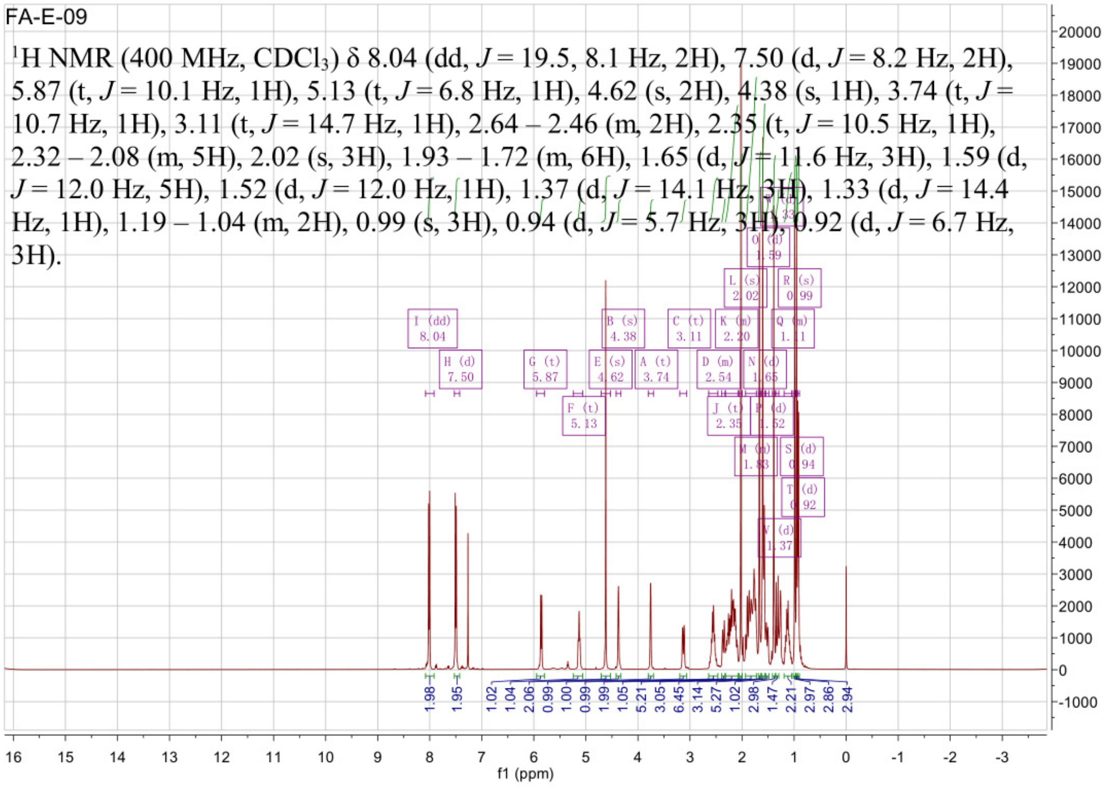

^{1}H NMR of compound FA-14

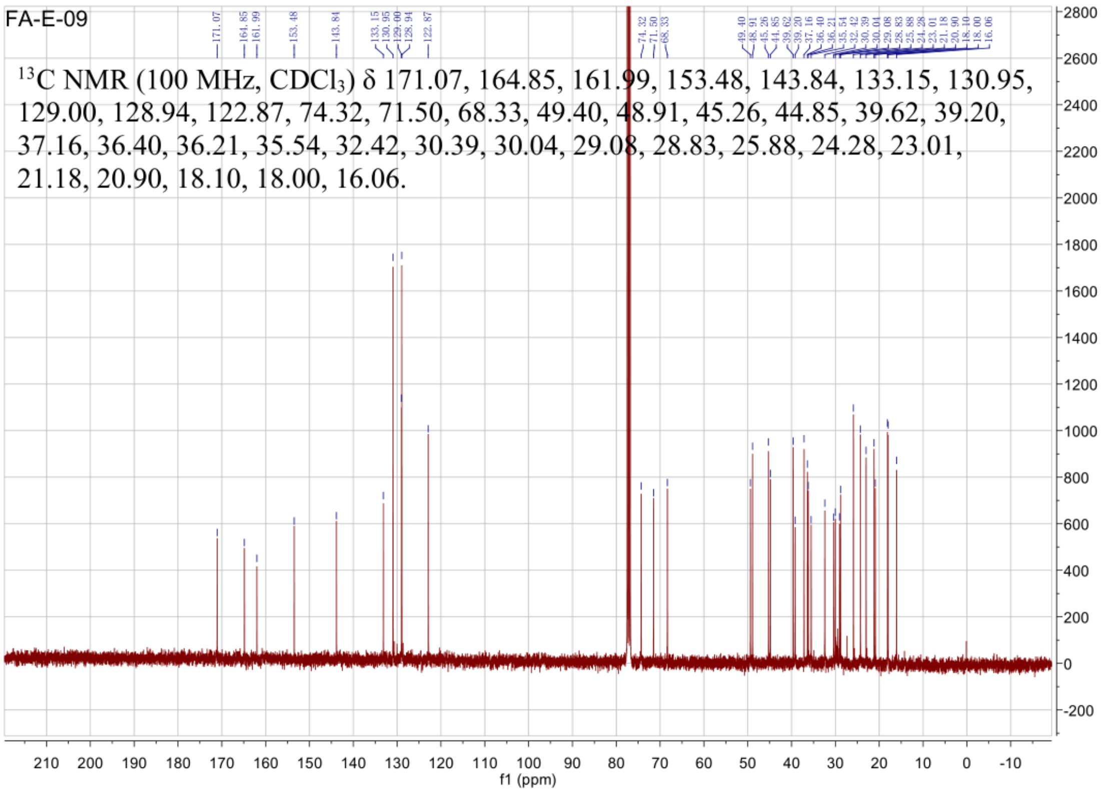

^{13}C NMR of compound FA-14

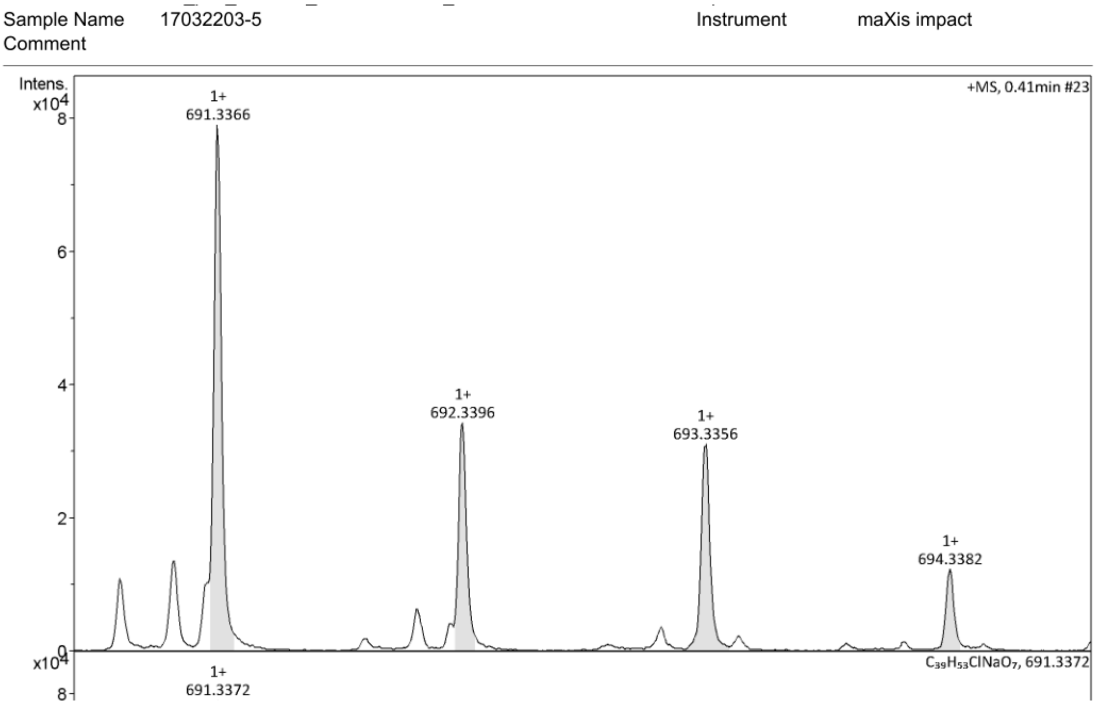

HRMS of compound FA-14

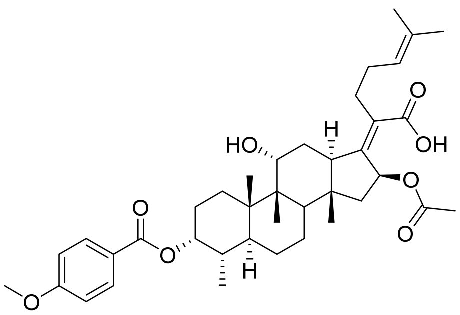

Structure of FA-15

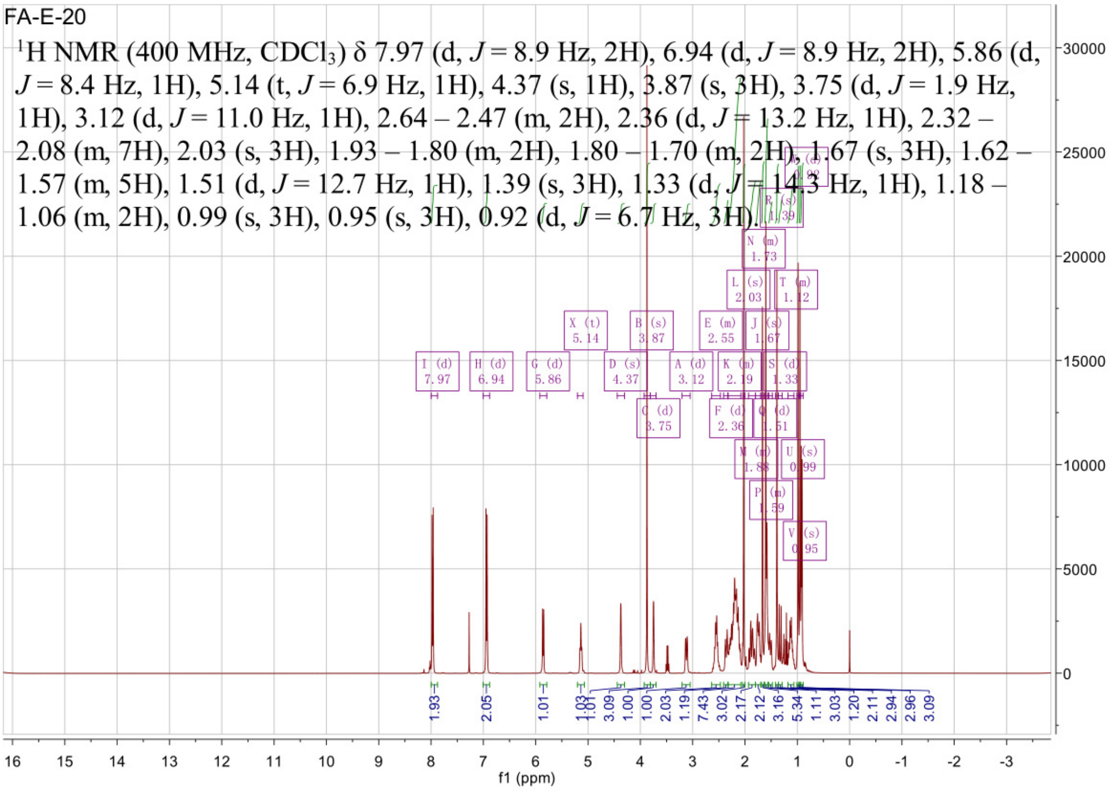

^{1}H NMR of compound FA-15

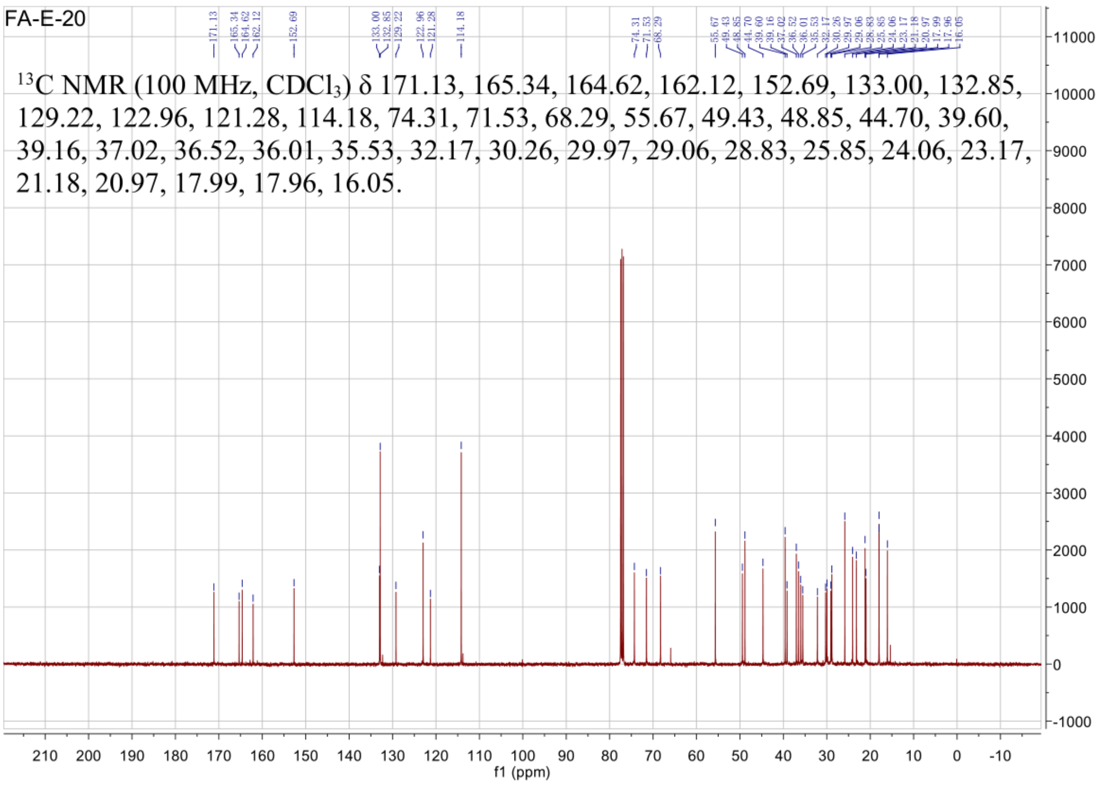

^{13}C NMR of compound FA-15

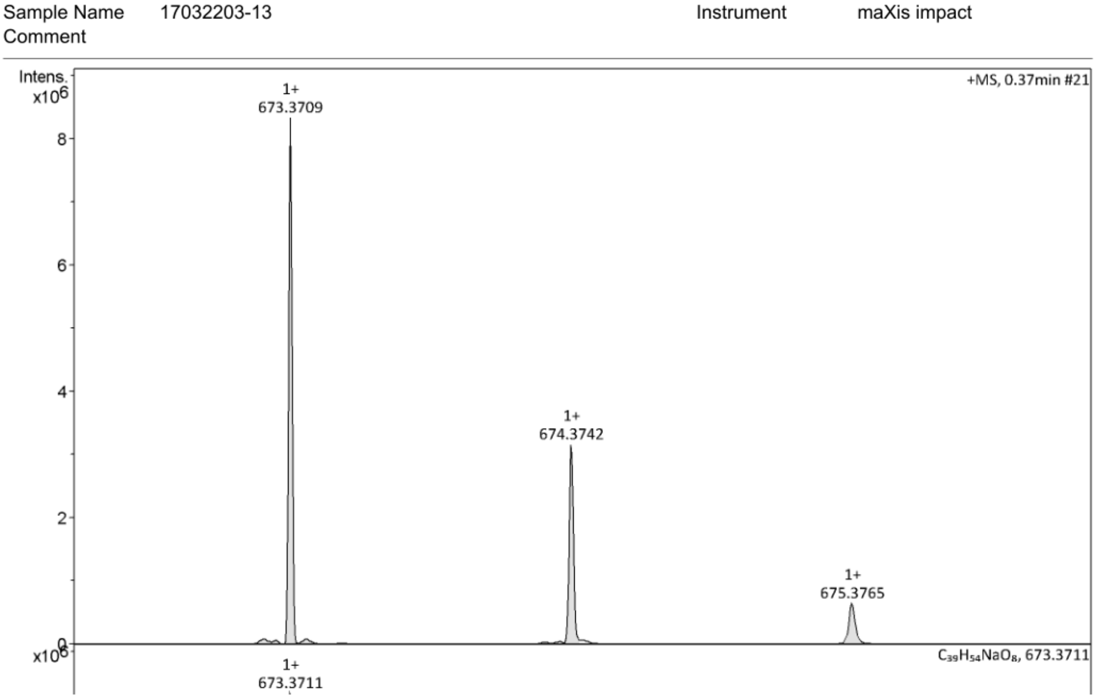

HRMS of compound FA-15

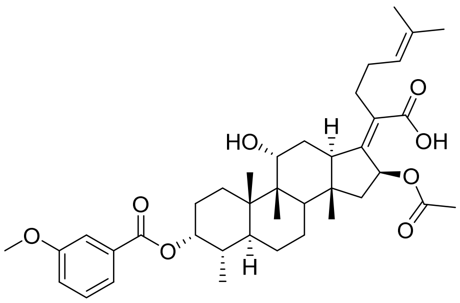

Structure of FA-16

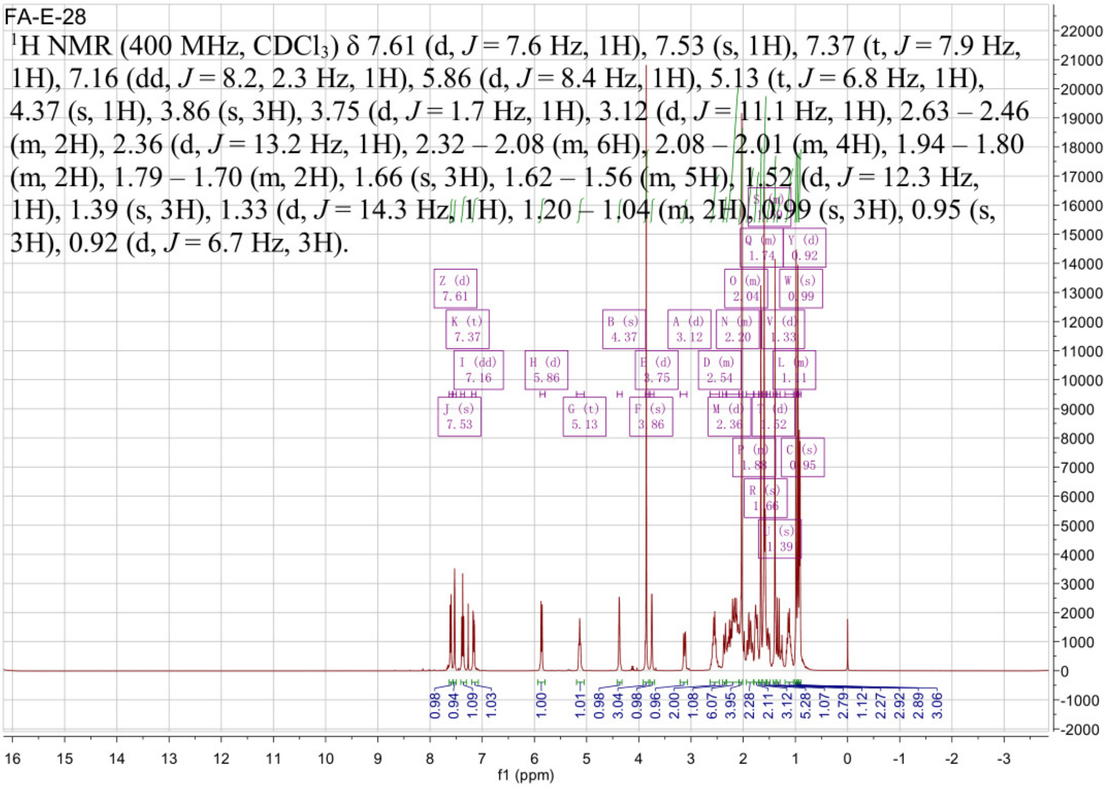

^{1}H NMR of compound FA-16

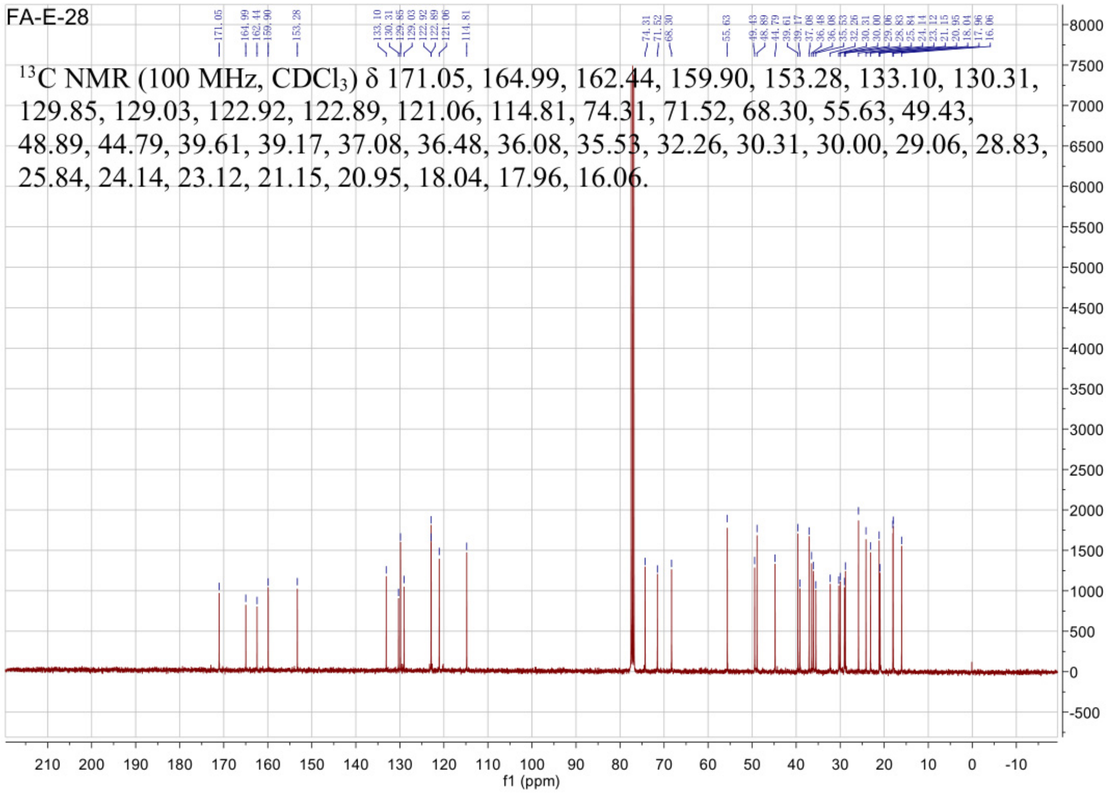

^{13}C NMR of compound FA-16

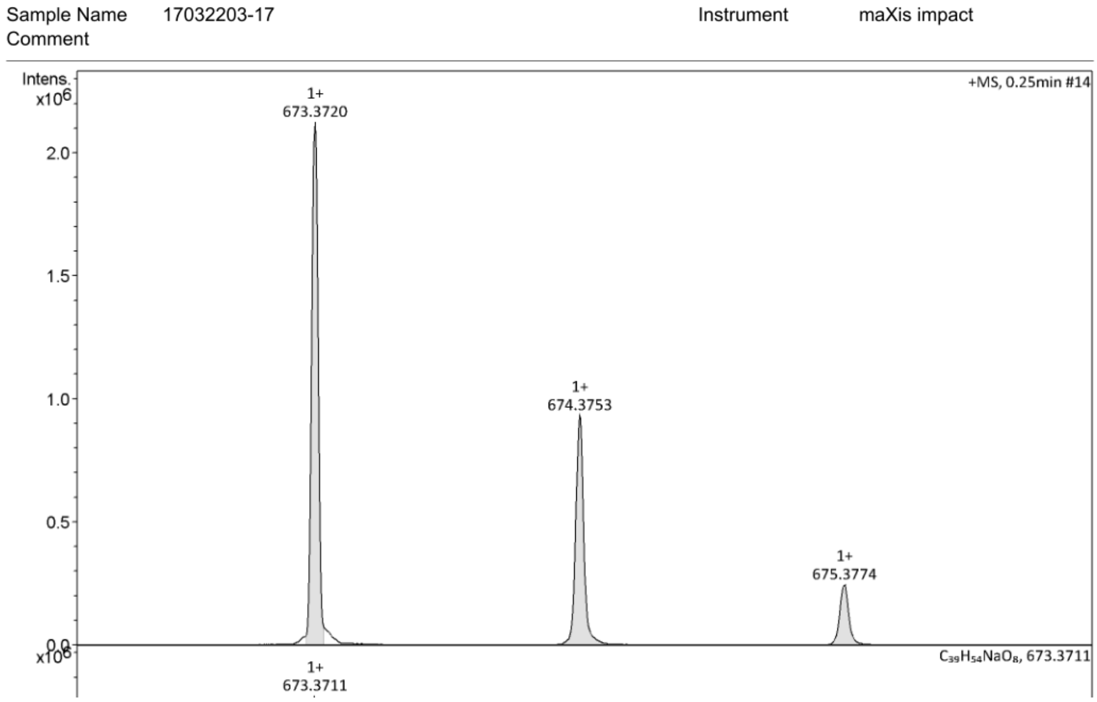

HRMS of compound FA-16

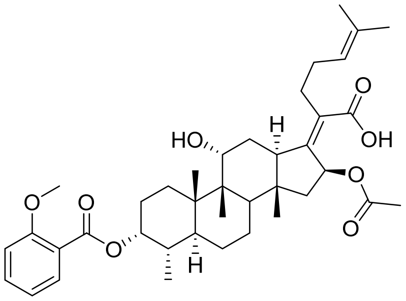

Structure of FA-17

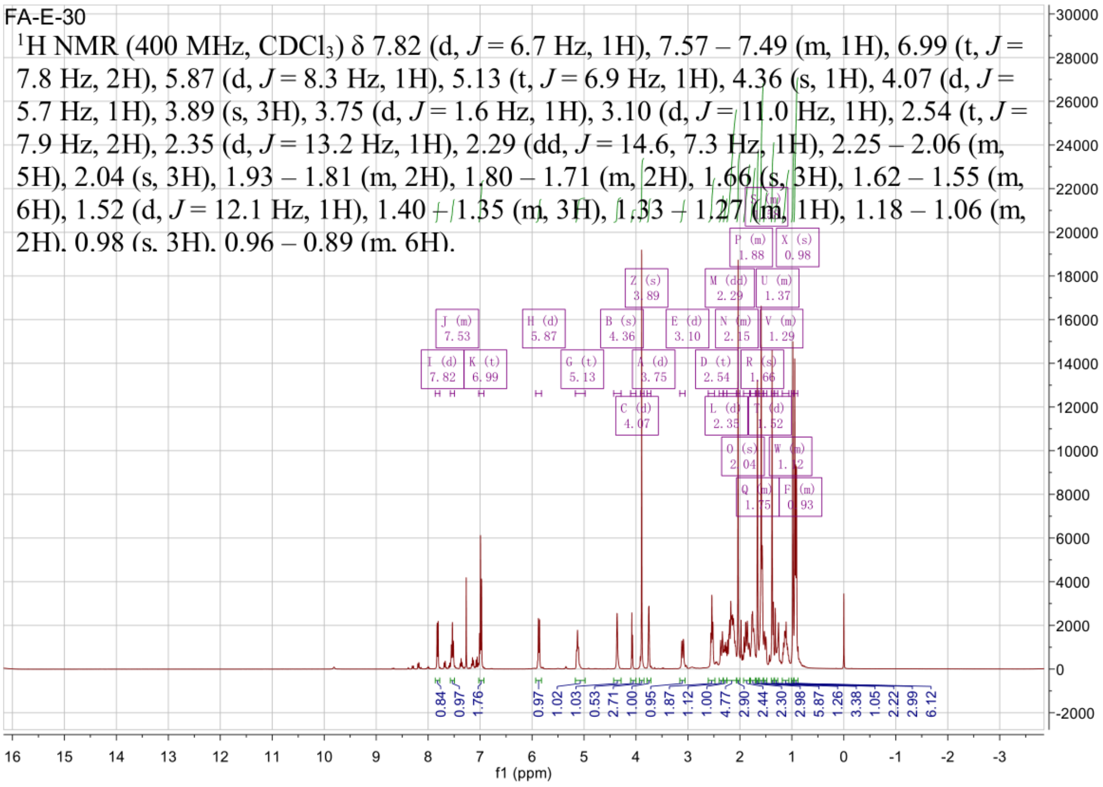

^{1}H NMR of compound FA-17

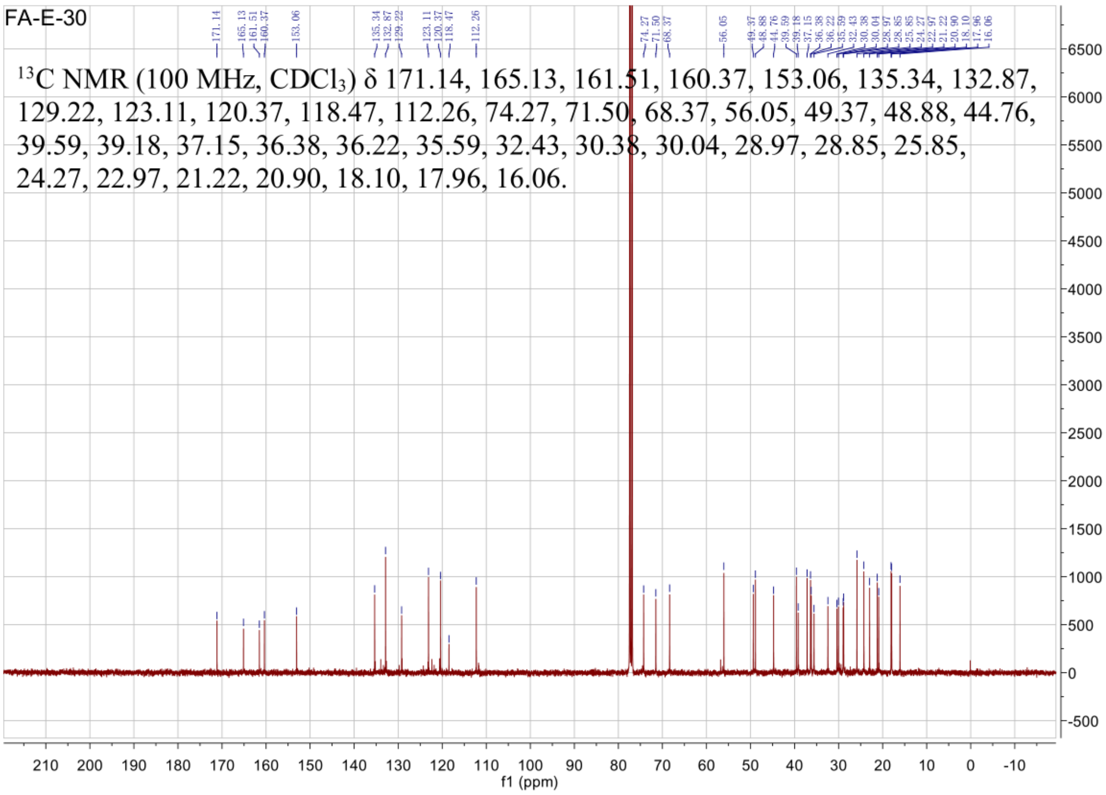

^{13}C NMR of compound FA-17

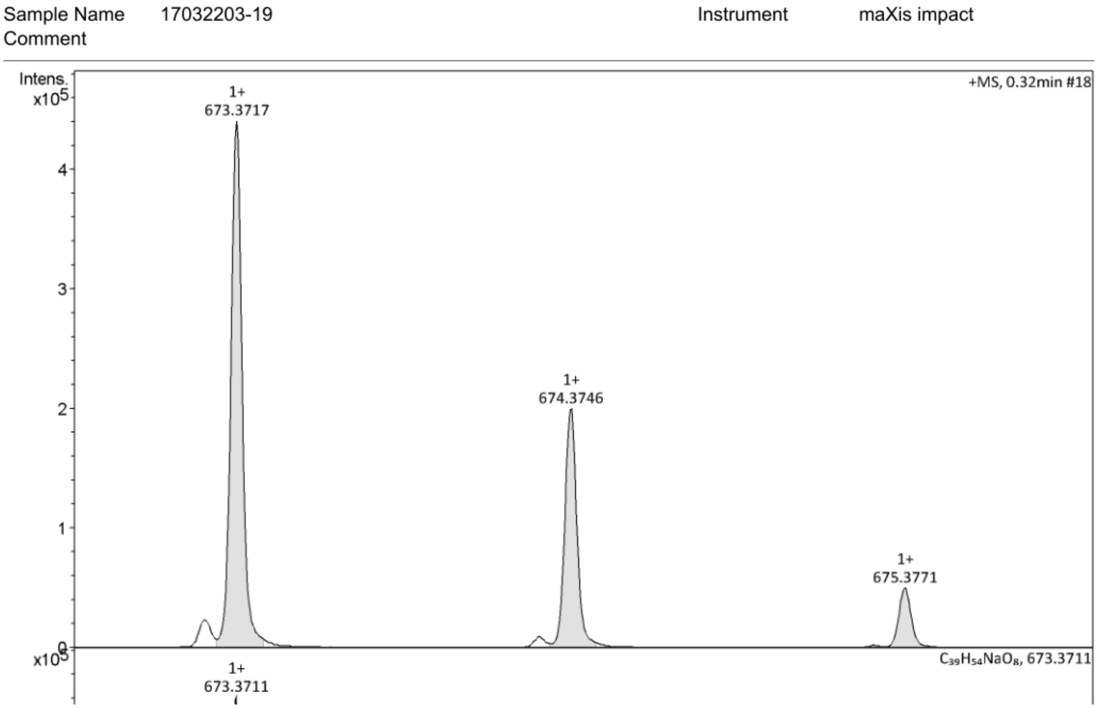

HRMS of compound FA-17

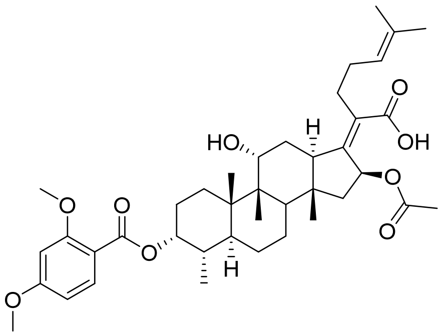

Structure of FA-18

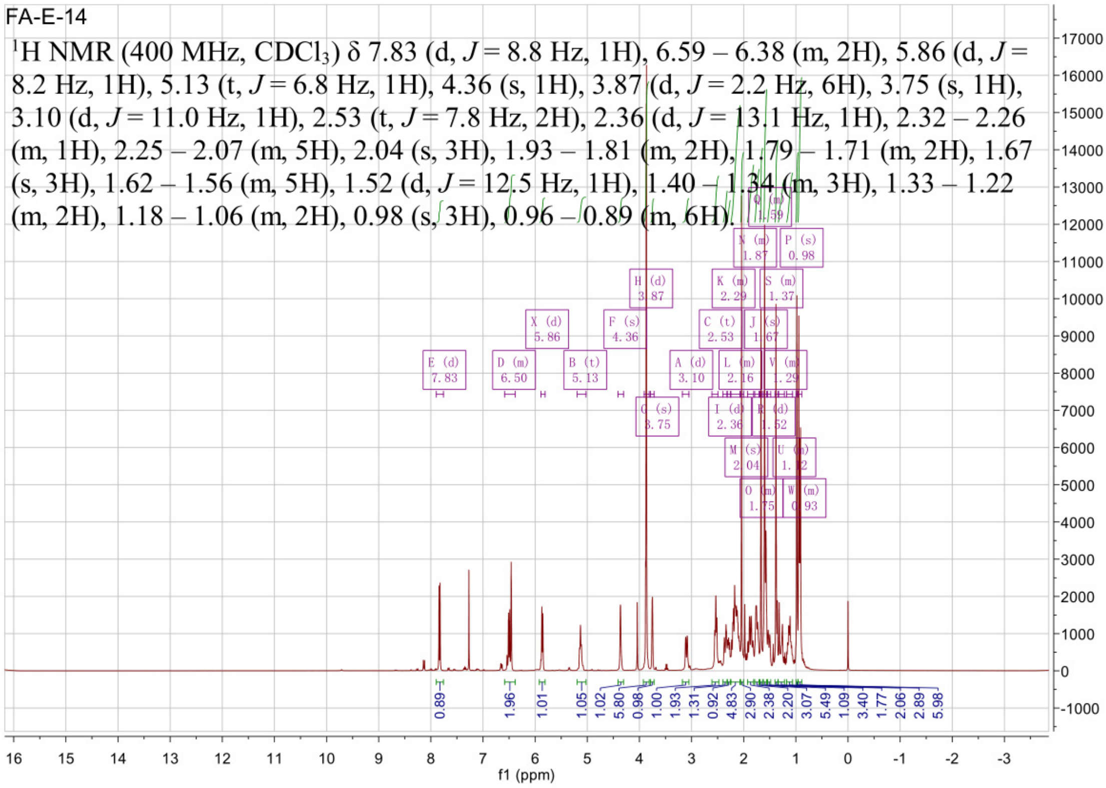

^{1}H NMR of compound FA-18

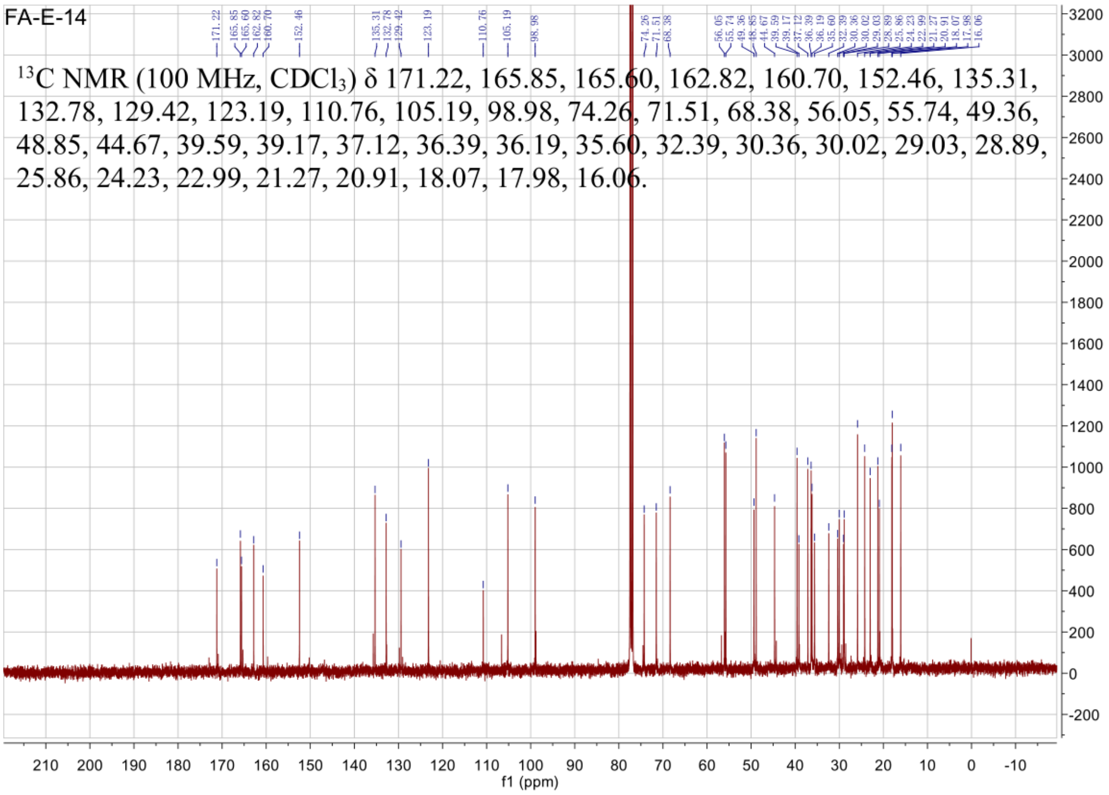

^{13}C NMR of compound FA-18

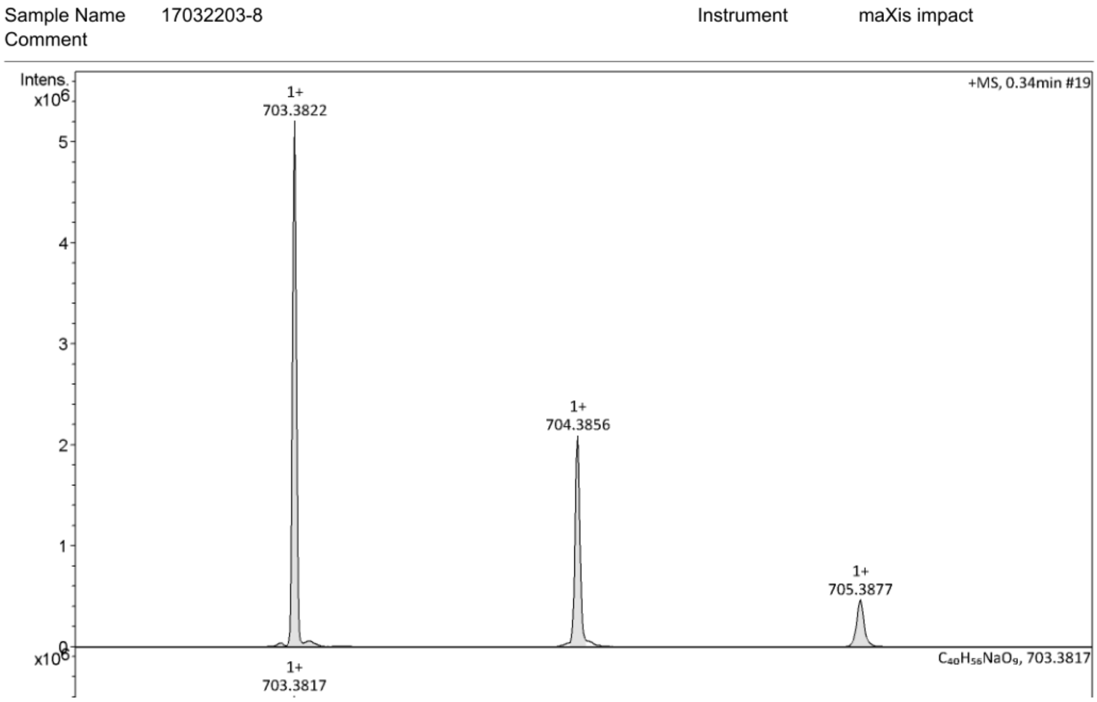

HRMS of compound FA-18

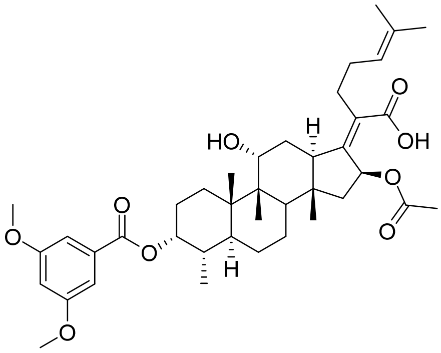

Structure of FA-19

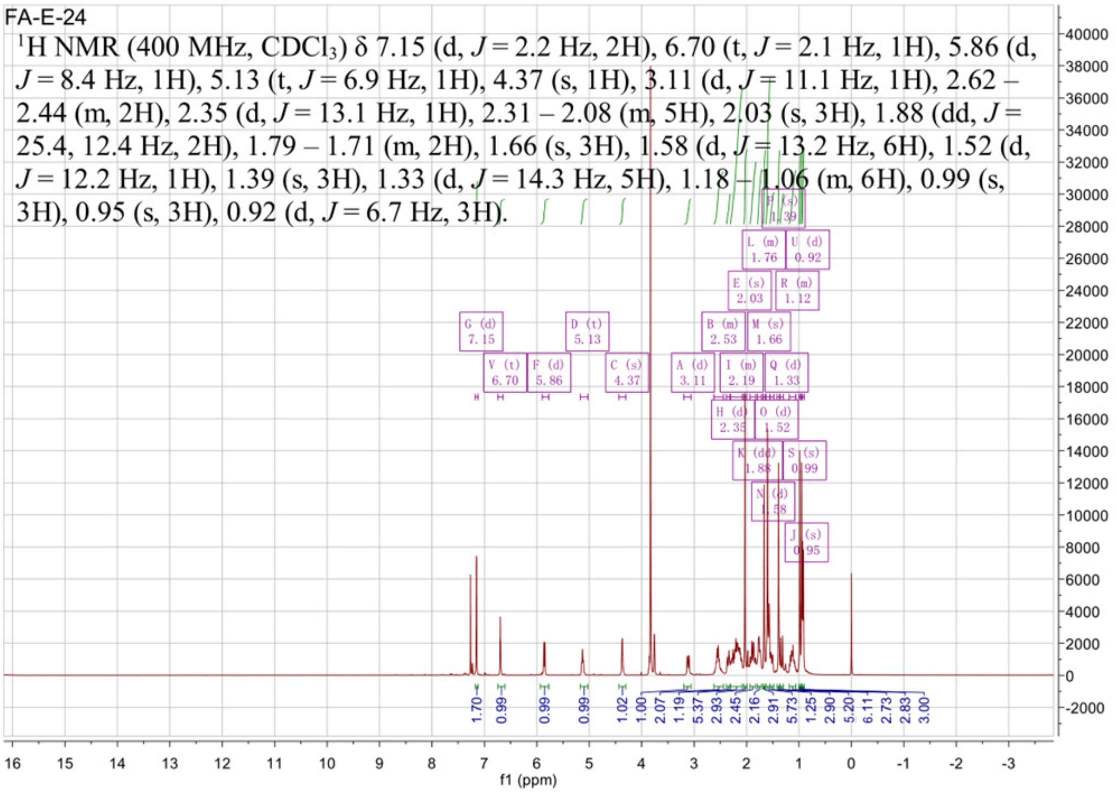

^{1}H NMR of compound FA-19

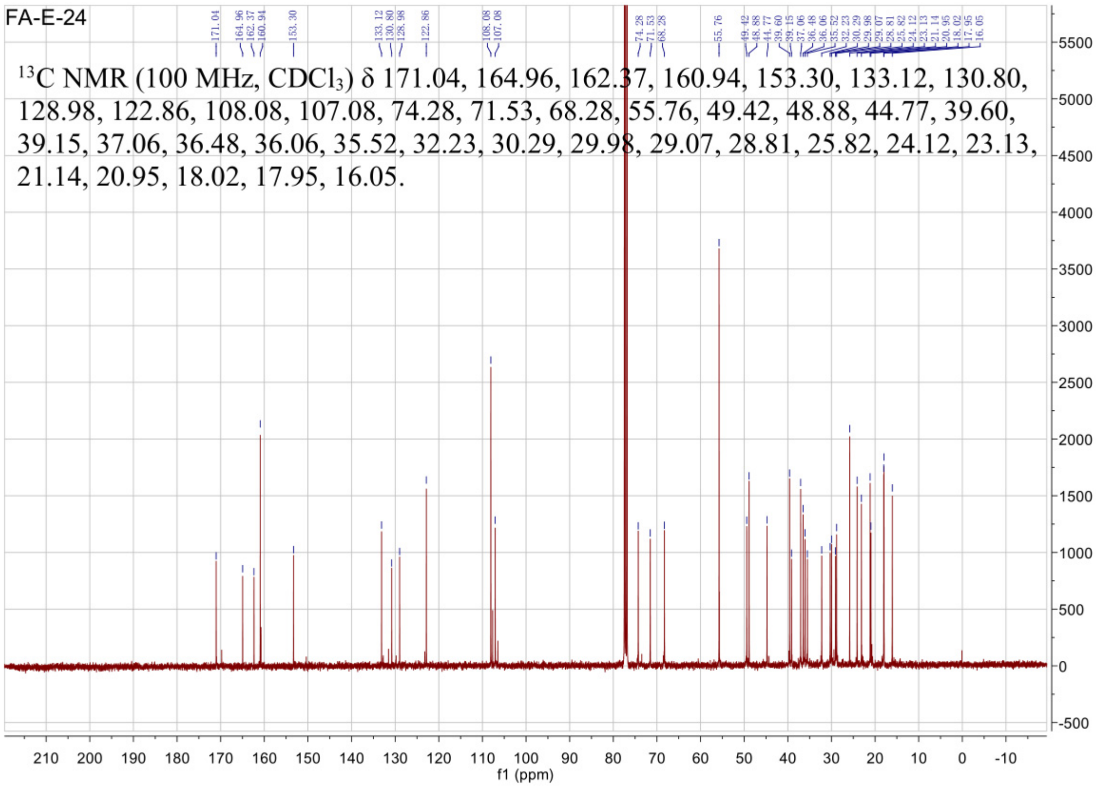

^{13}C NMR of compound FA-19

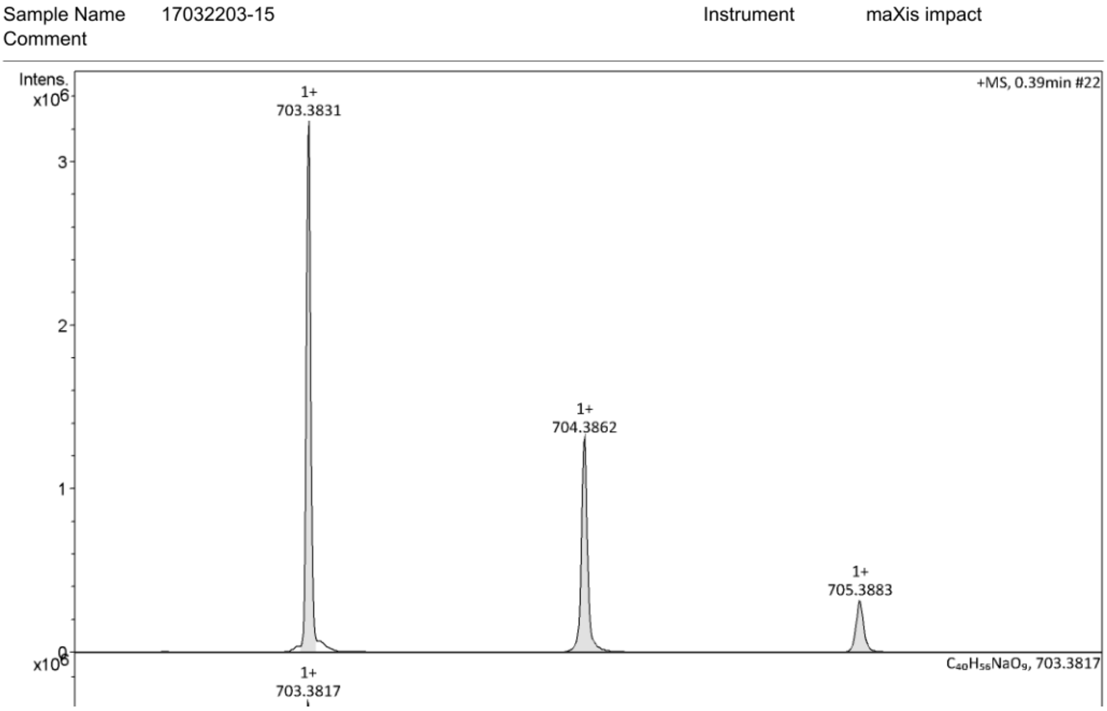

HRMS of compound FA-19

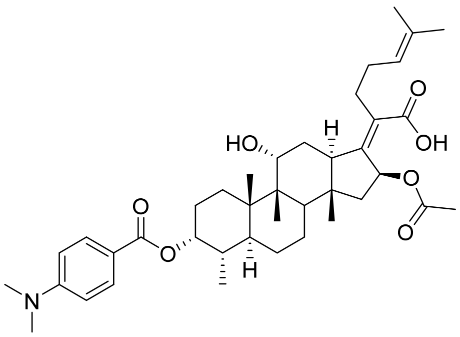

Structure of FA-20

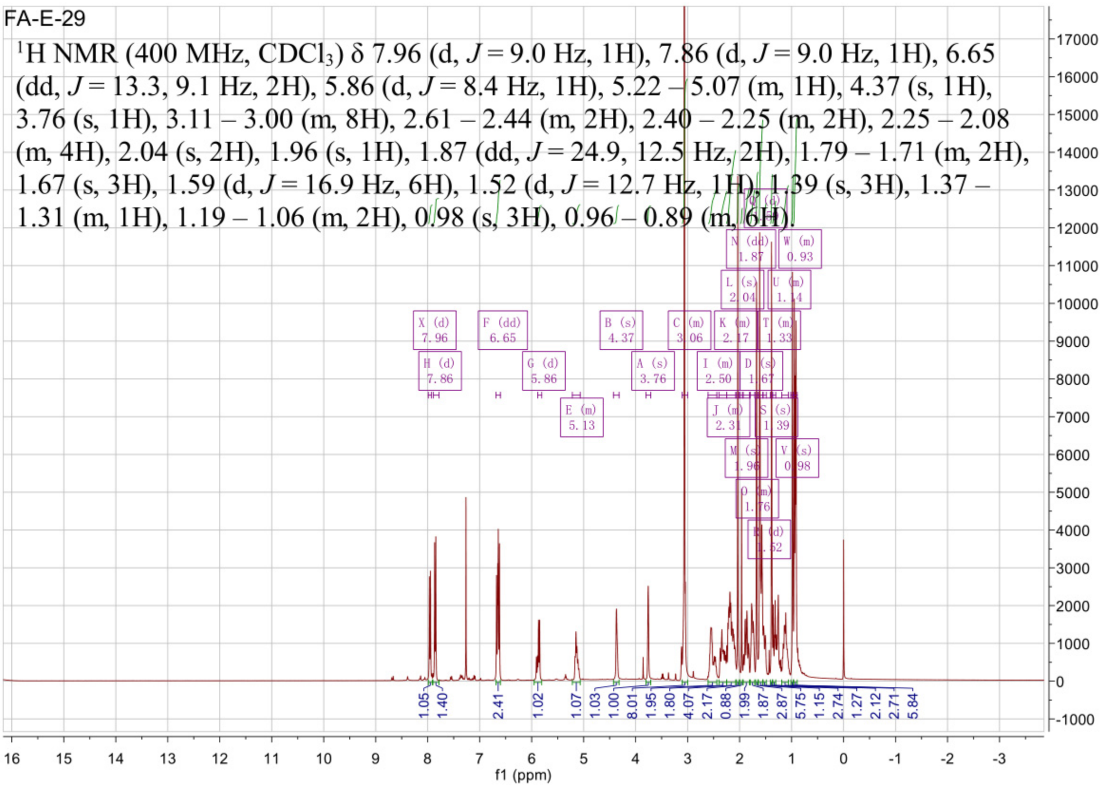

^{1}H NMR of compound FA-20

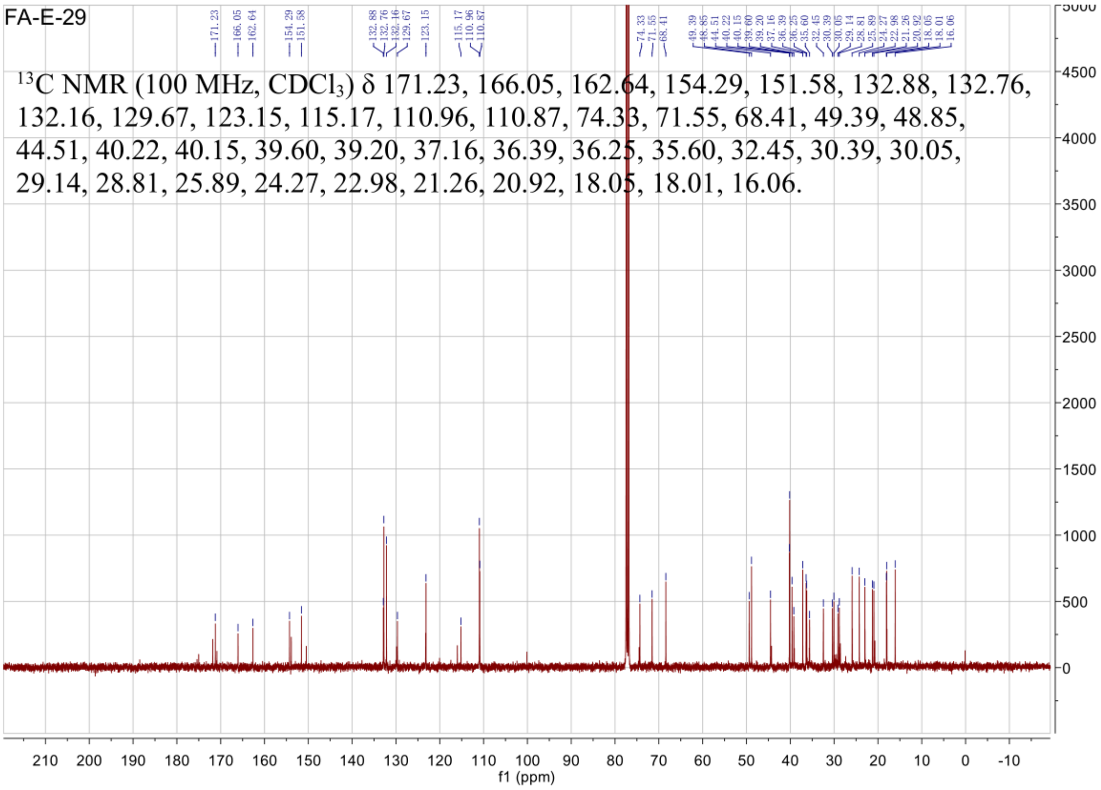

^{13}C NMR of compound FA-20

Sample Name 17032203-18 Instrument maXis impact
Comment

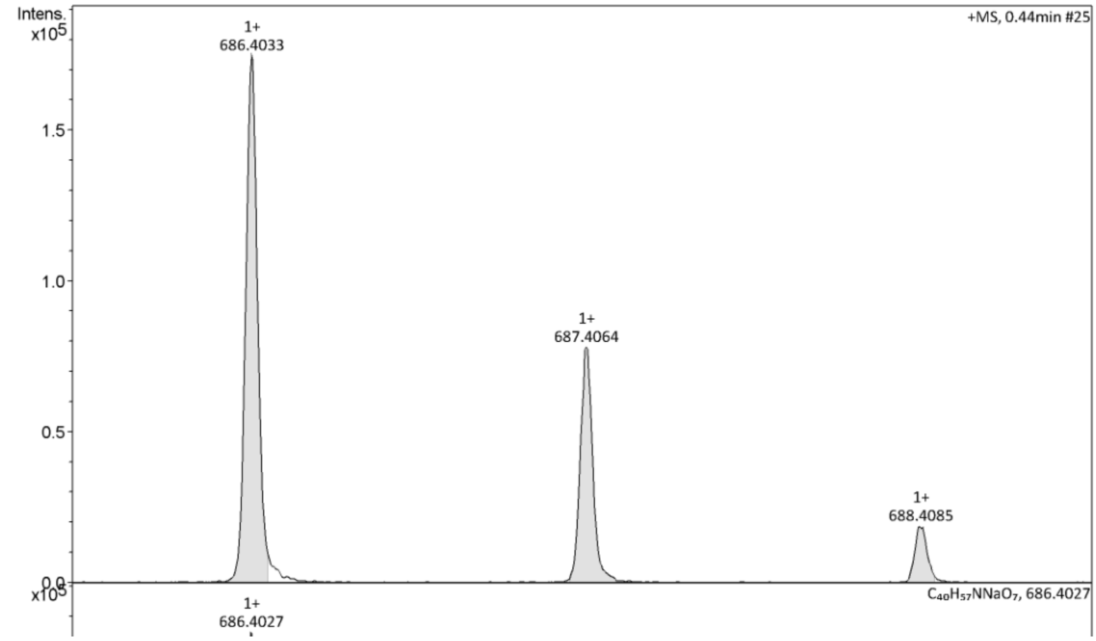

HRMS of compound FA-20

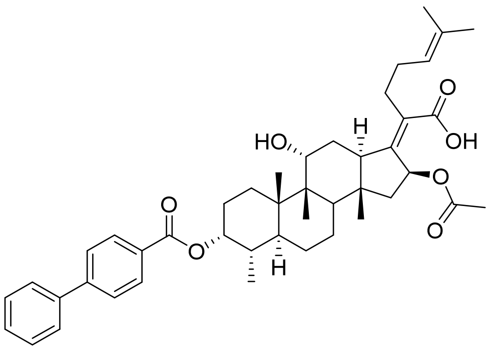

Structure of FA-21

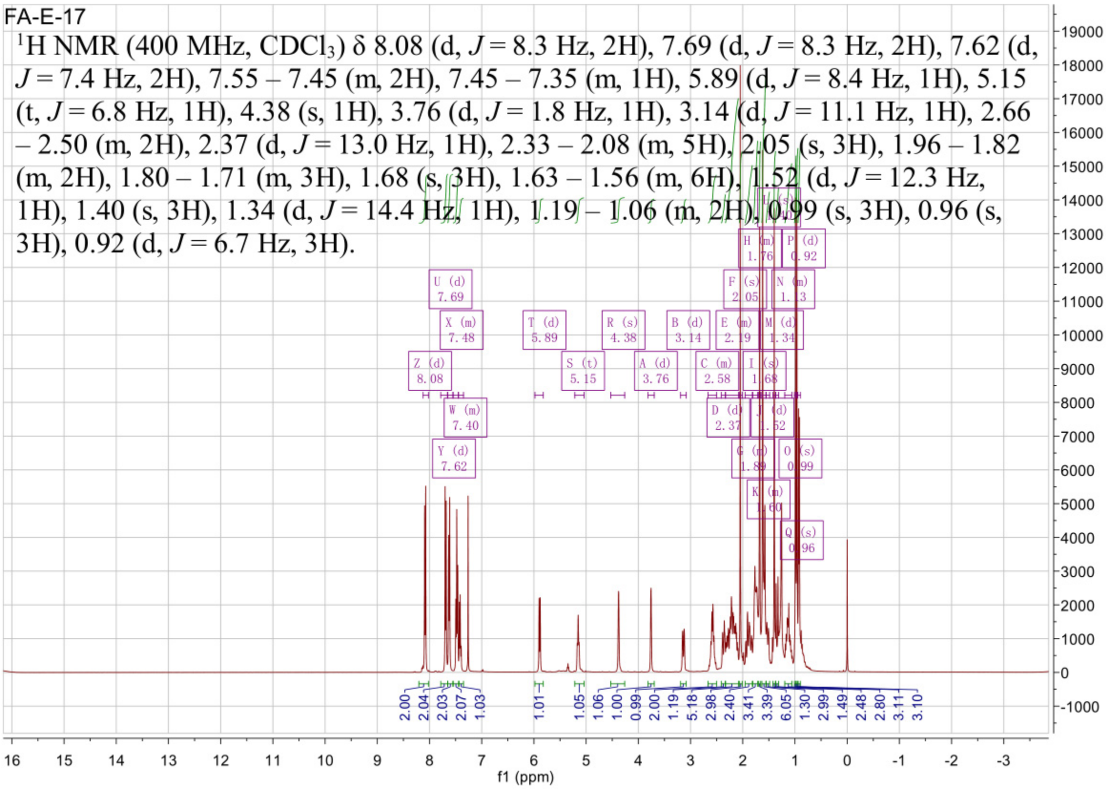

^{1}H NMR of compound FA-21

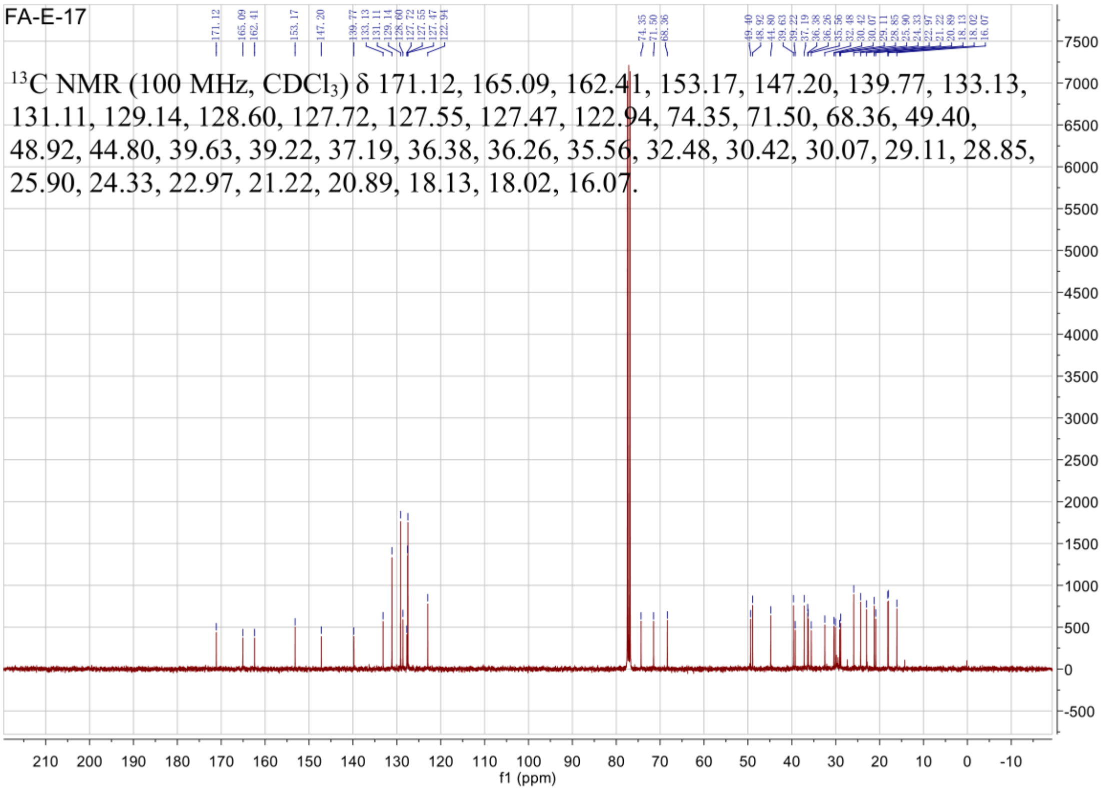

^{13}C NMR of compound FA-21

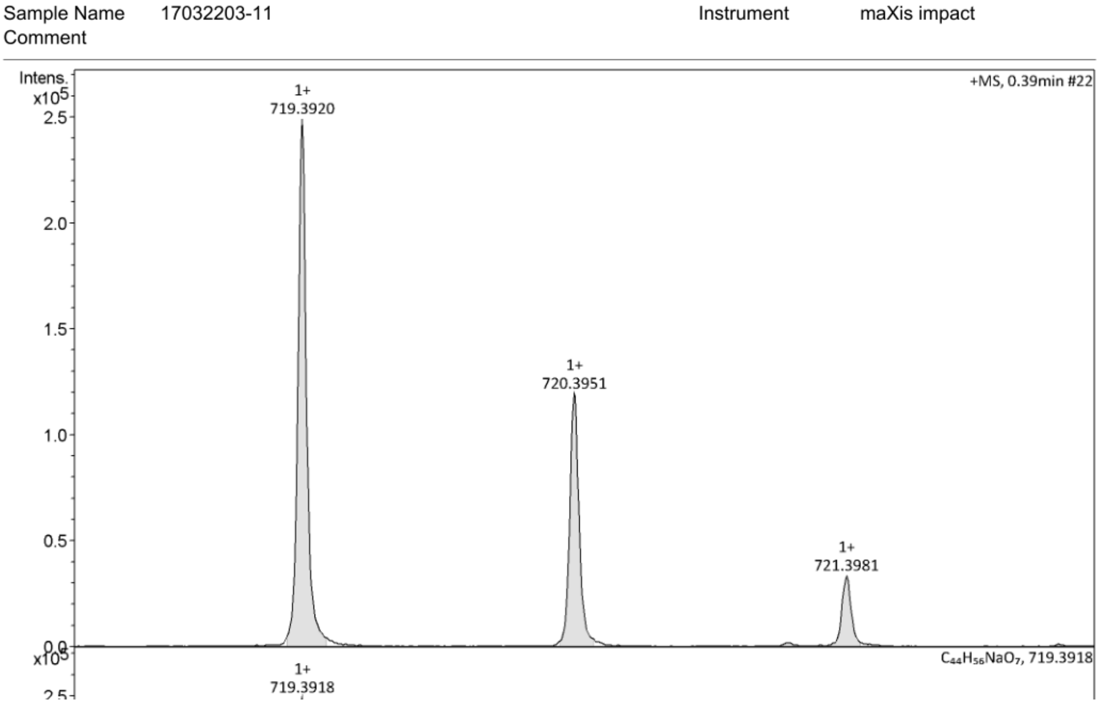

HRMS of compound FA-21

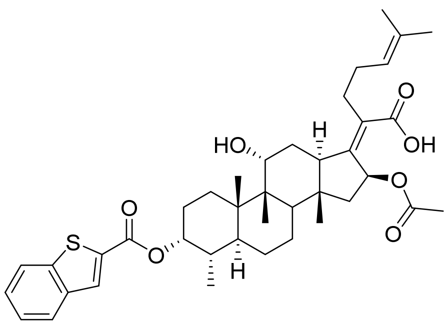

Structure of FA-22

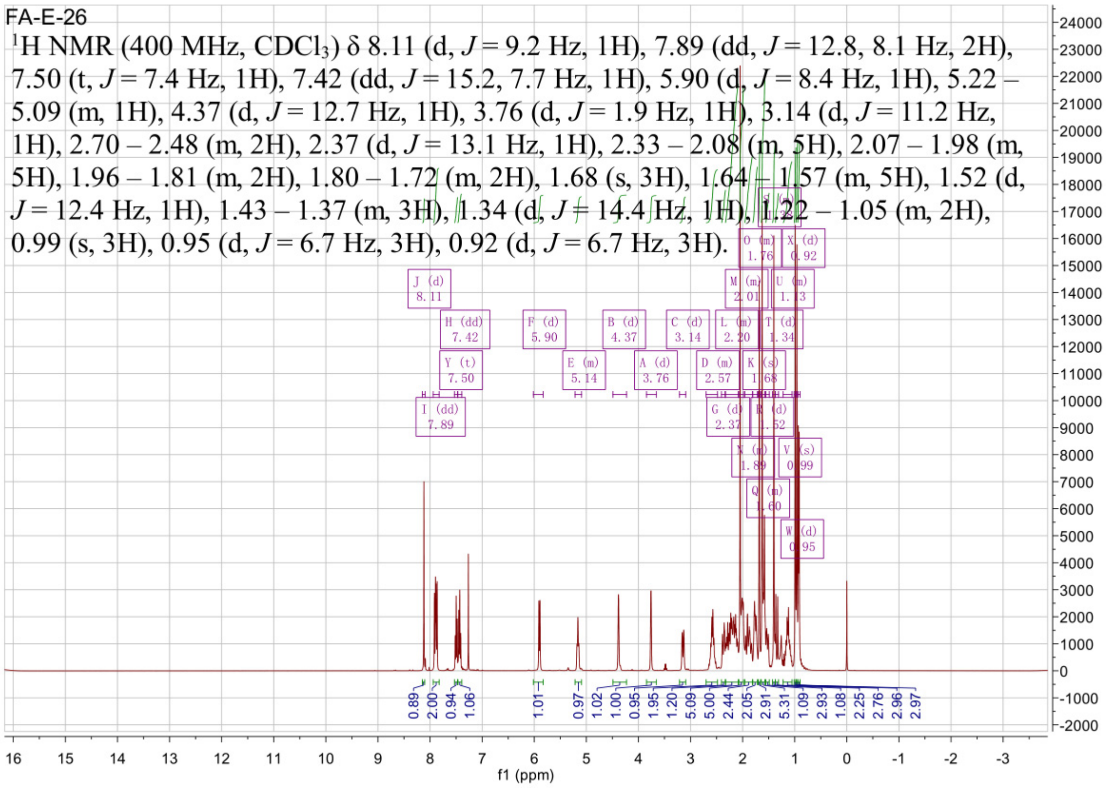

^{1}H NMR of compound FA-22

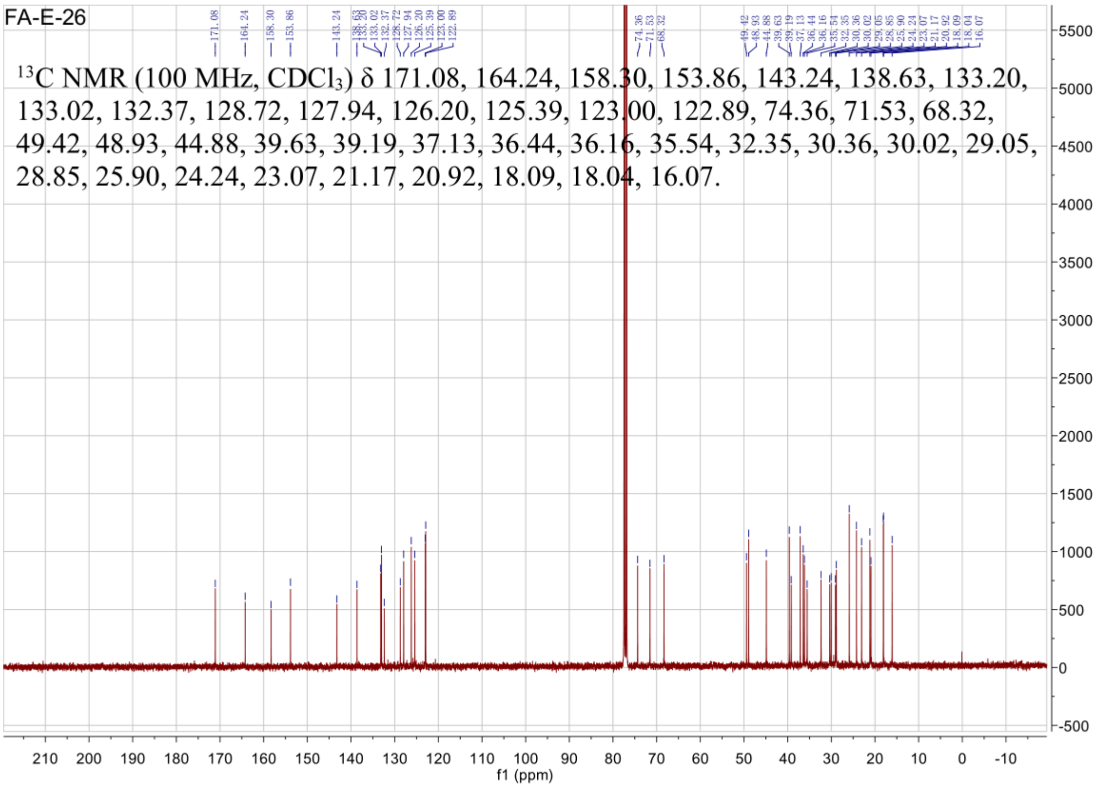

^{13}C NMR of compound FA-22

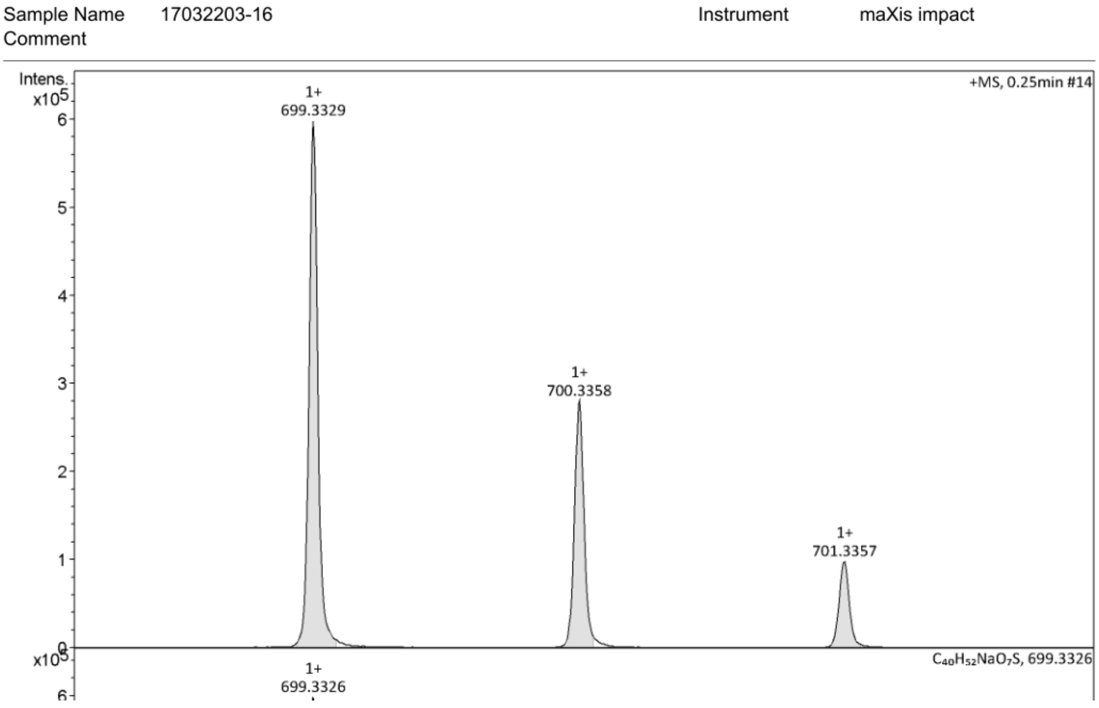

HRMS of compound FA-22

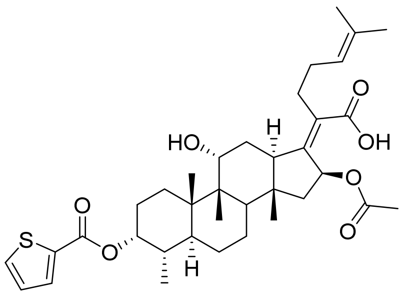

Structure of FA-23

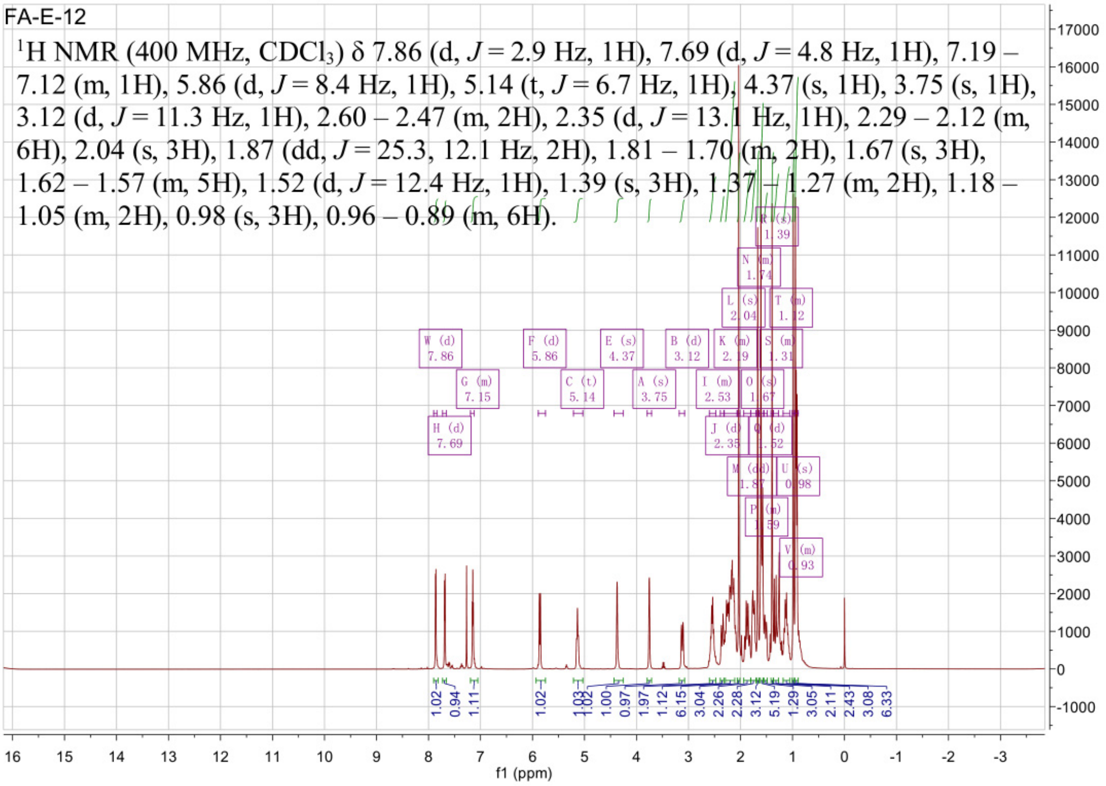

^{1}H NMR of compound FA-23

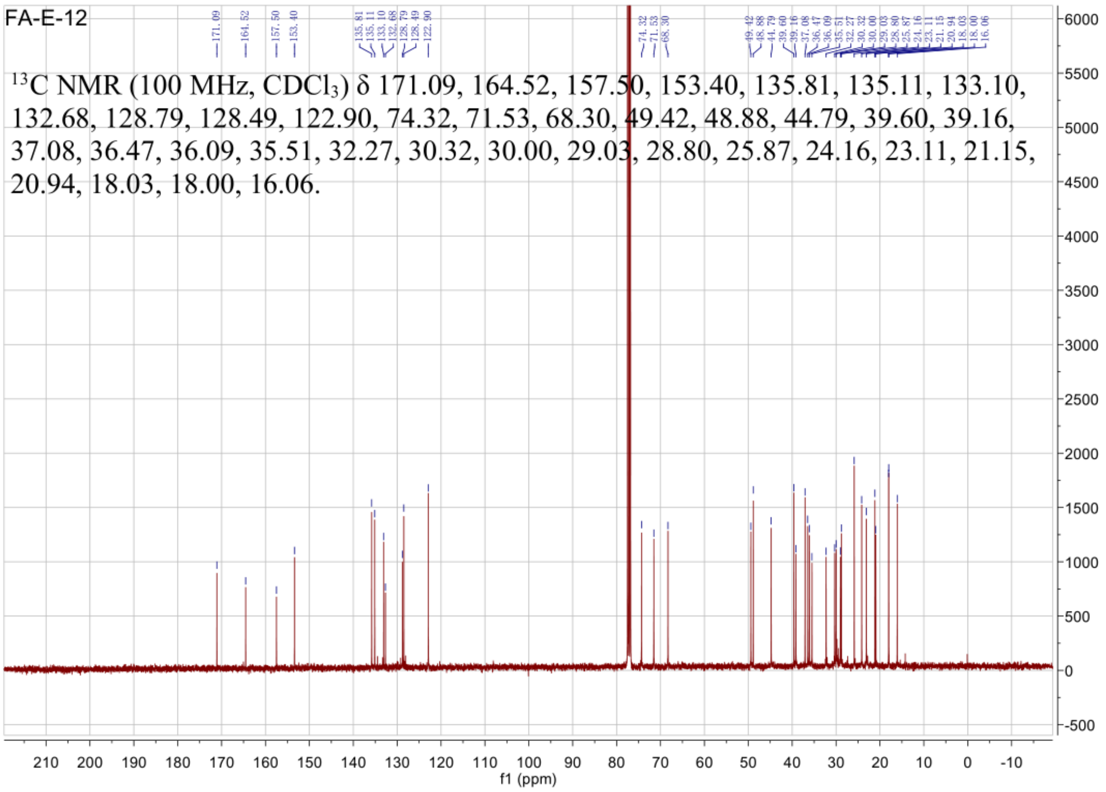

^{13}C NMR of compound FA-23

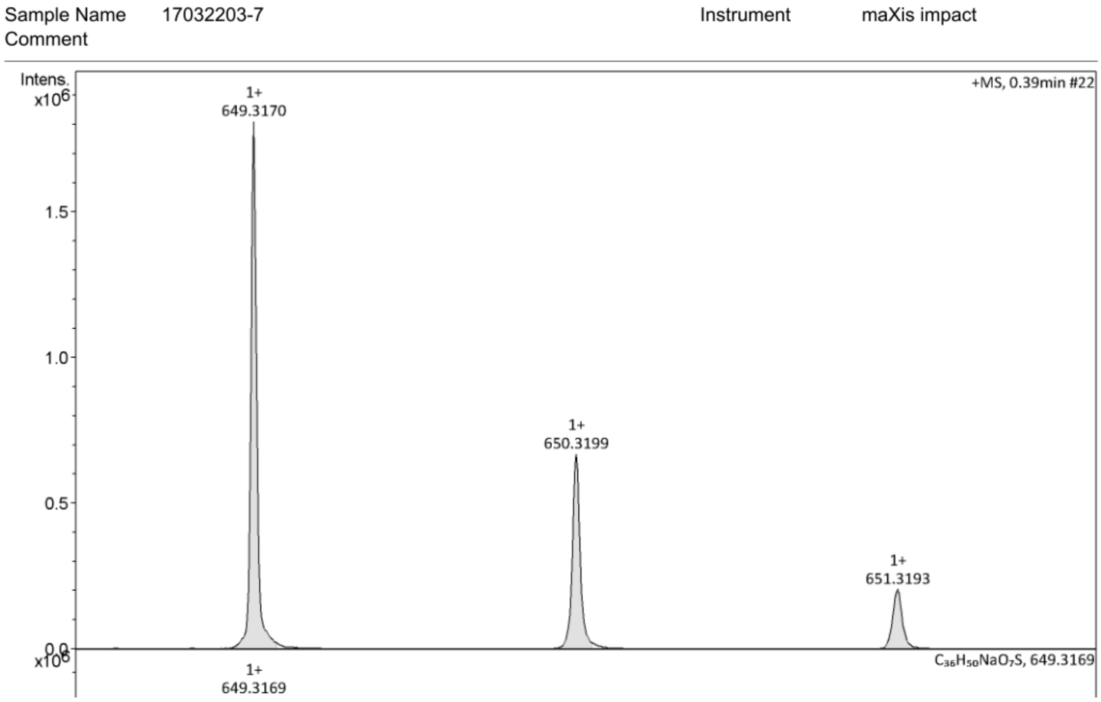

HRMS of compound FA-23

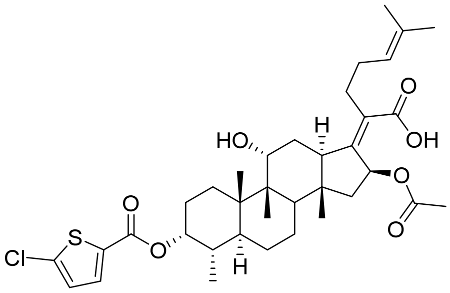

Structure of FA-24

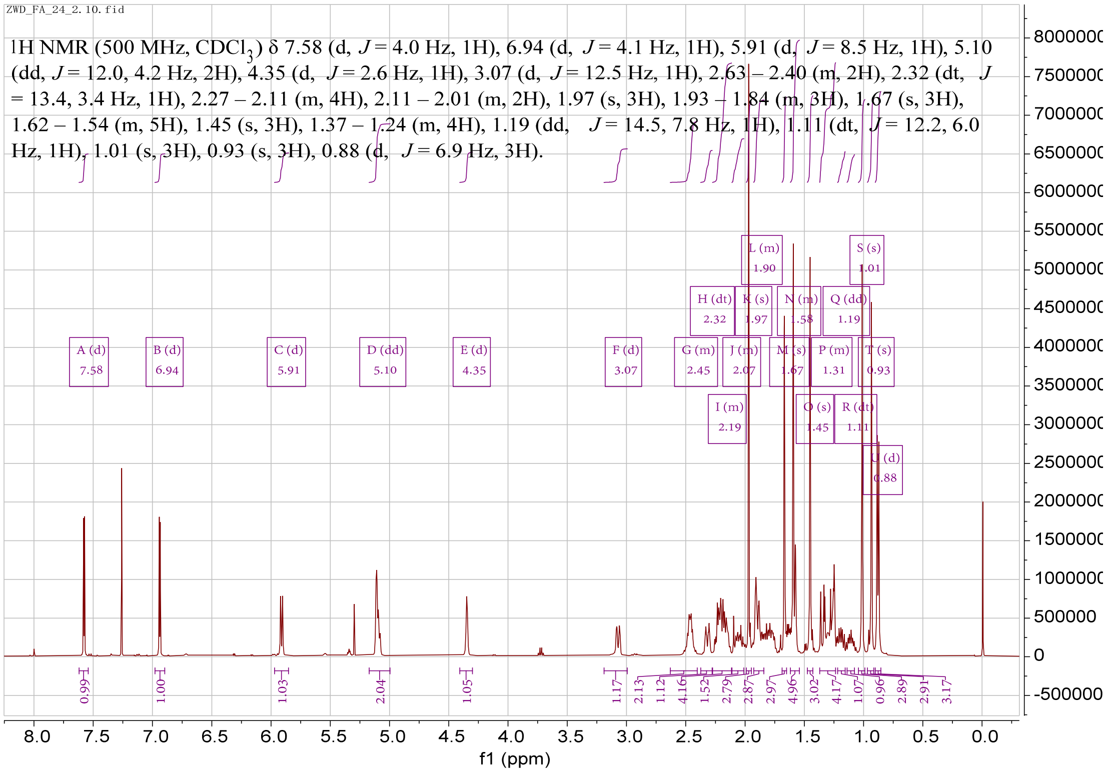

^{1}H NMR of compound FA-24

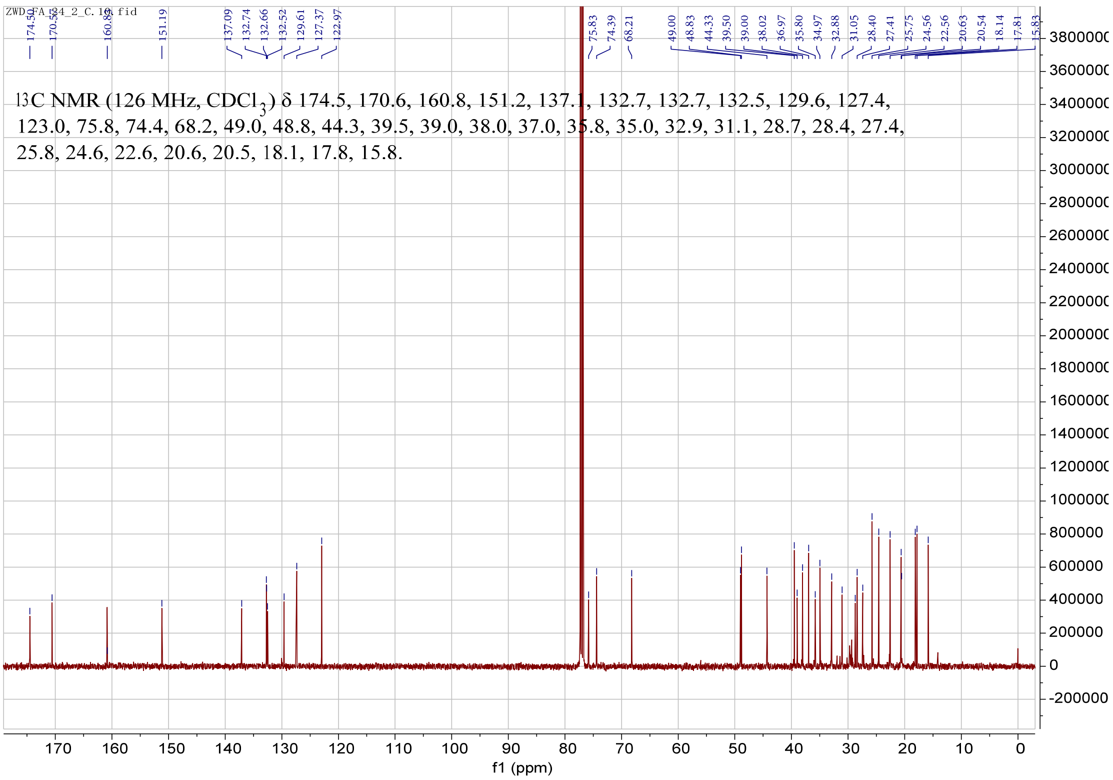

^{13}C NMR of compound FA-24

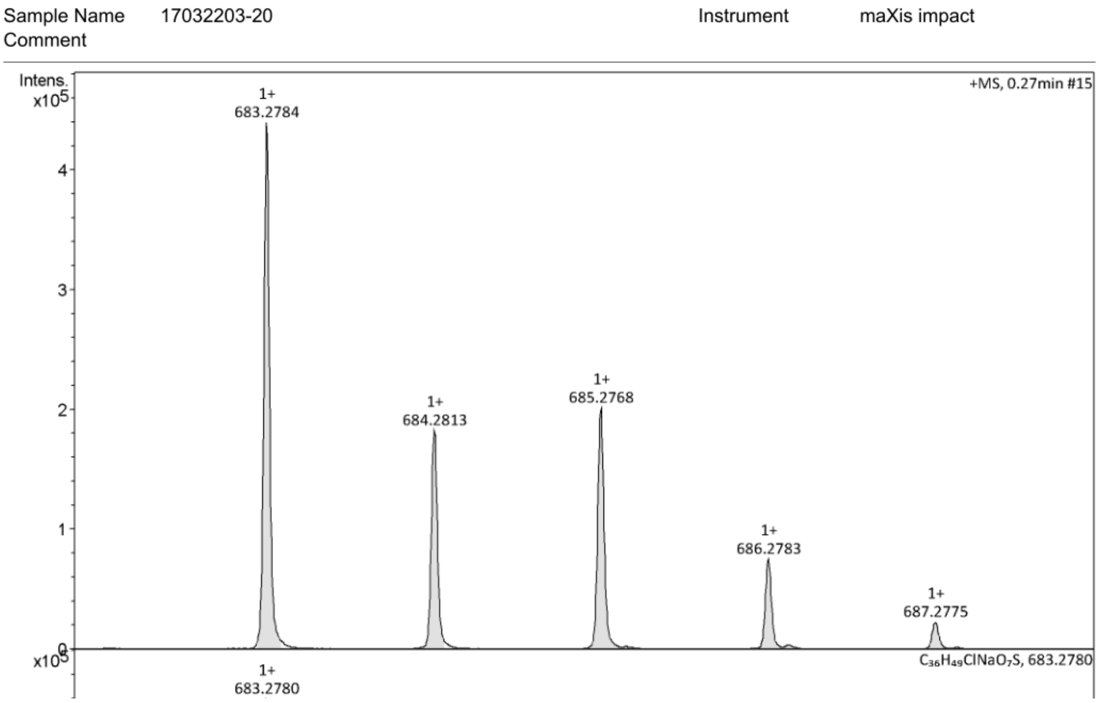

HRMS of compound FA-24

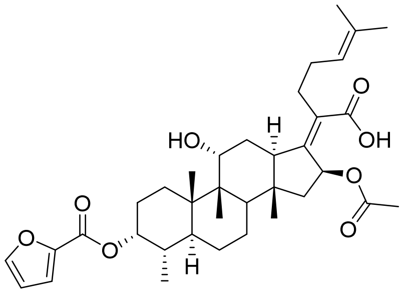

Structure of FA-25

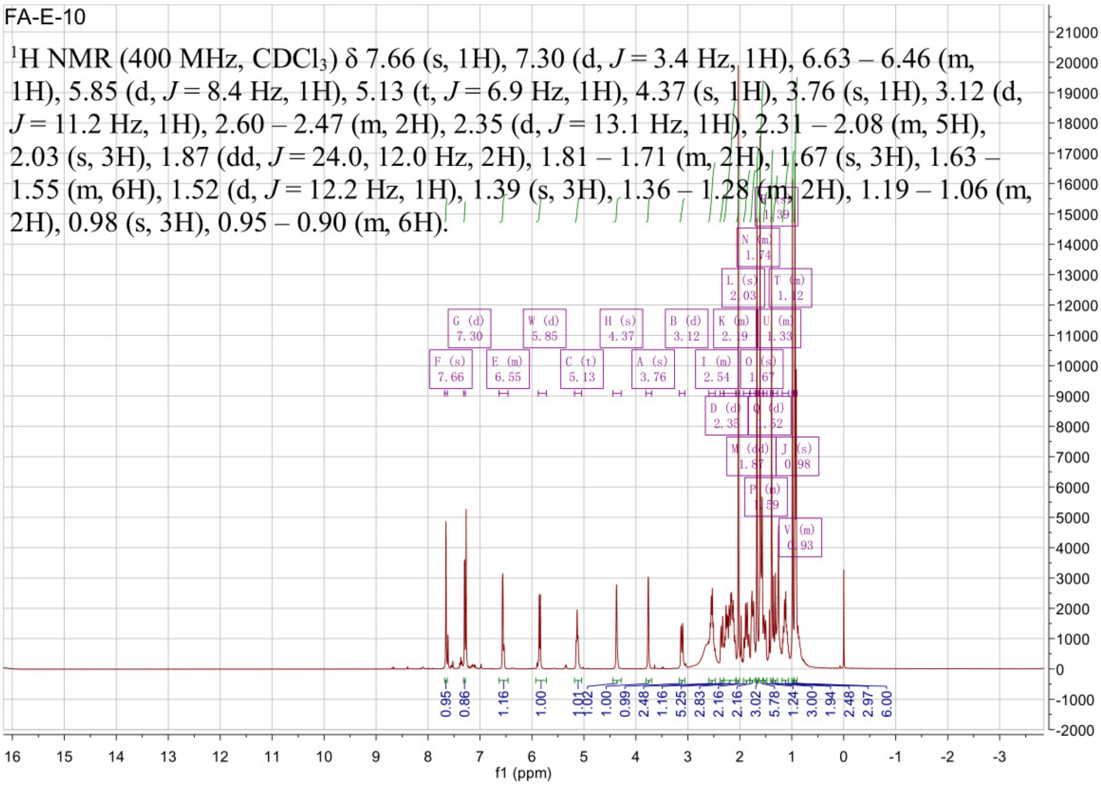

^{1}H NMR of compound FA-25

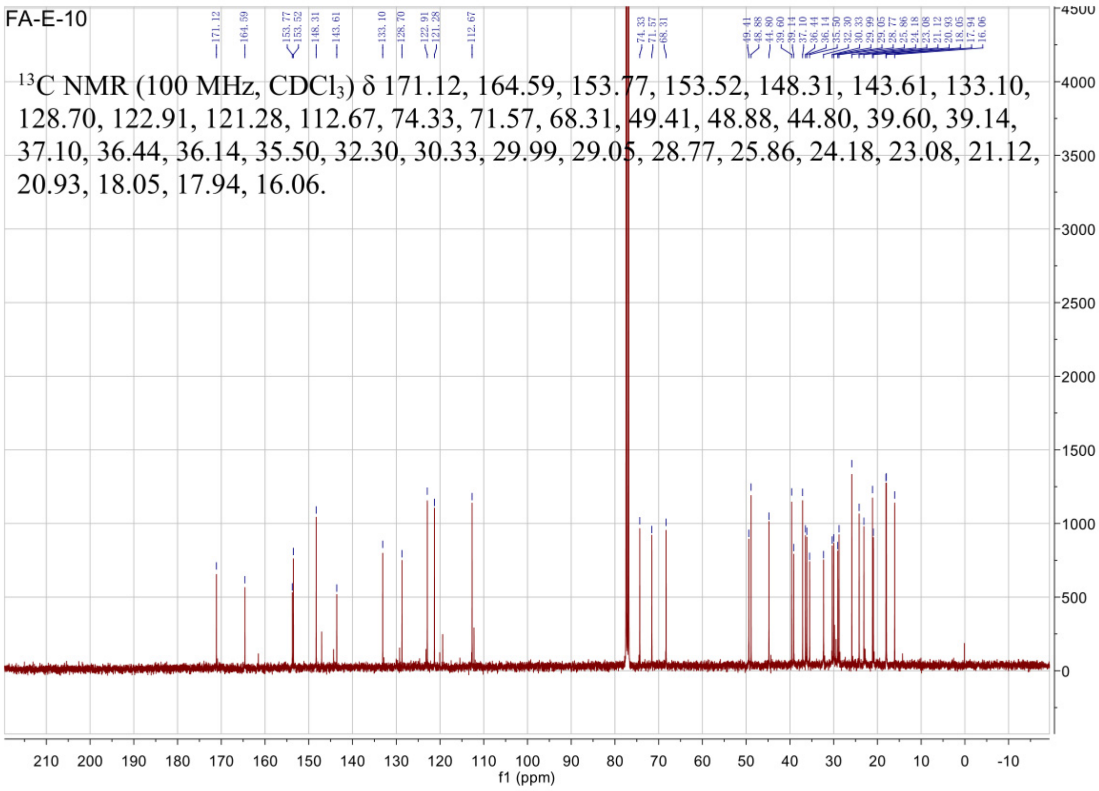

^{13}C NMR of compound FA-25

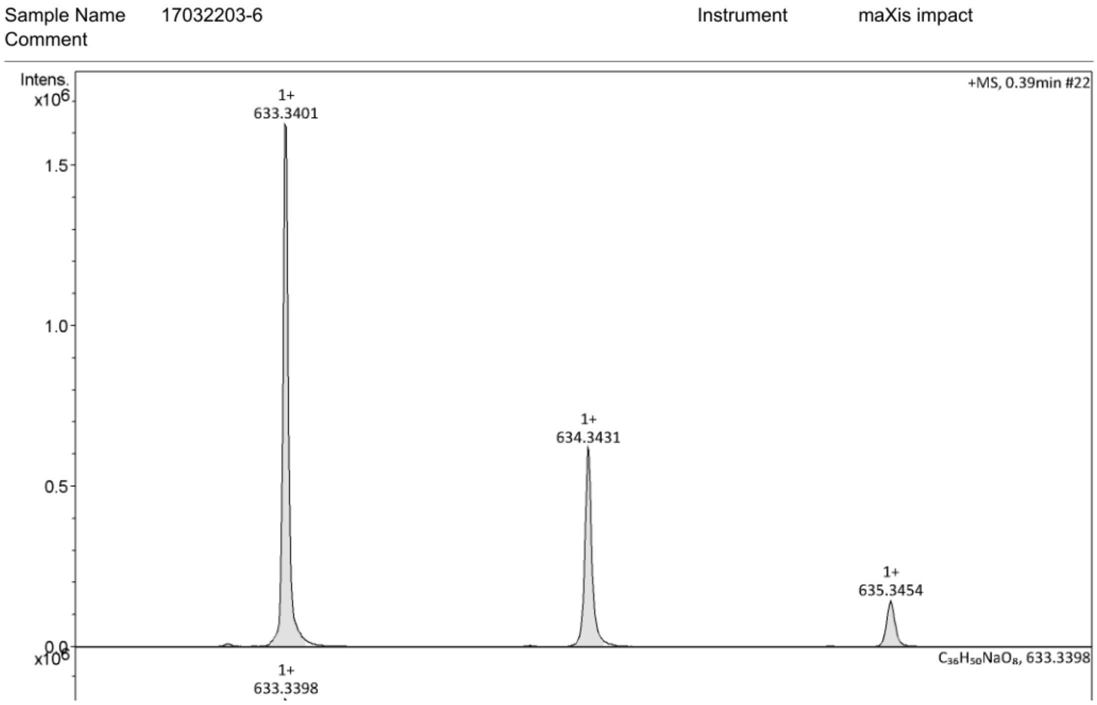

HRMS of compound FA-25

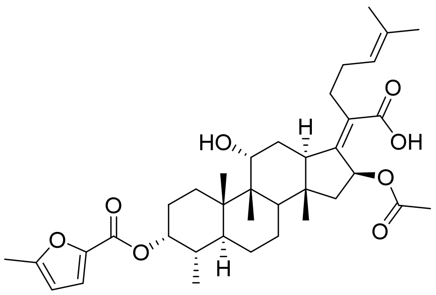

Structure of FA-26

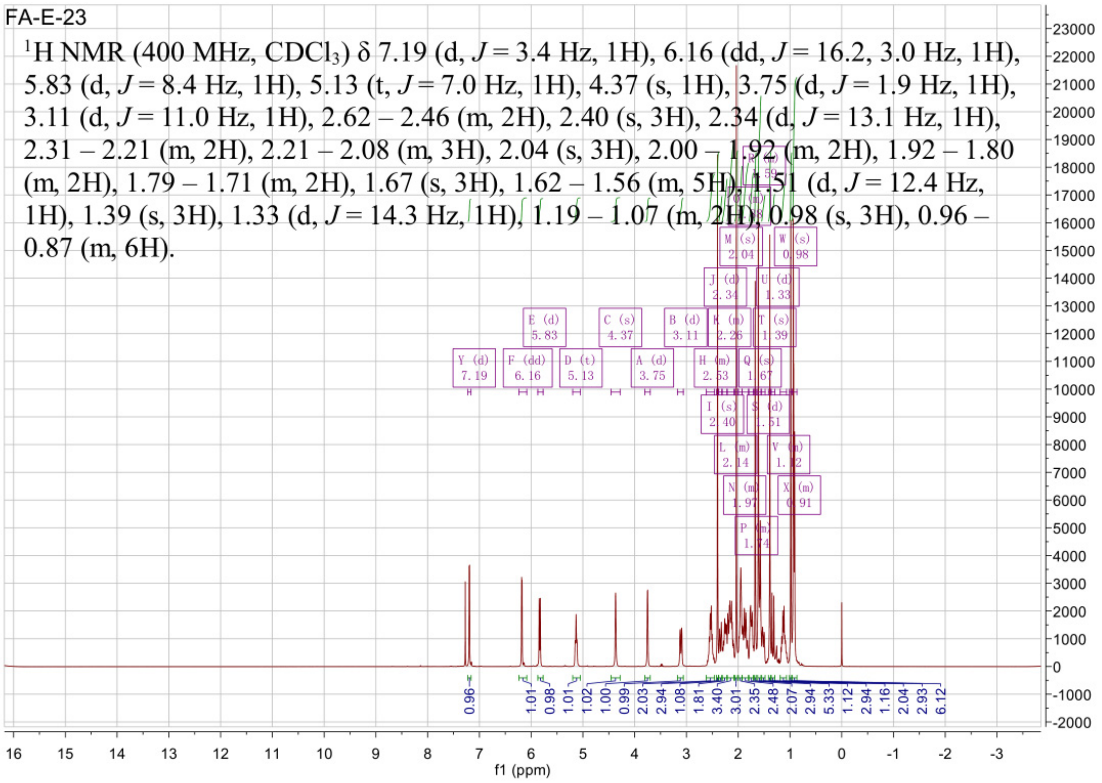

^{1}H NMR of compound FA-26

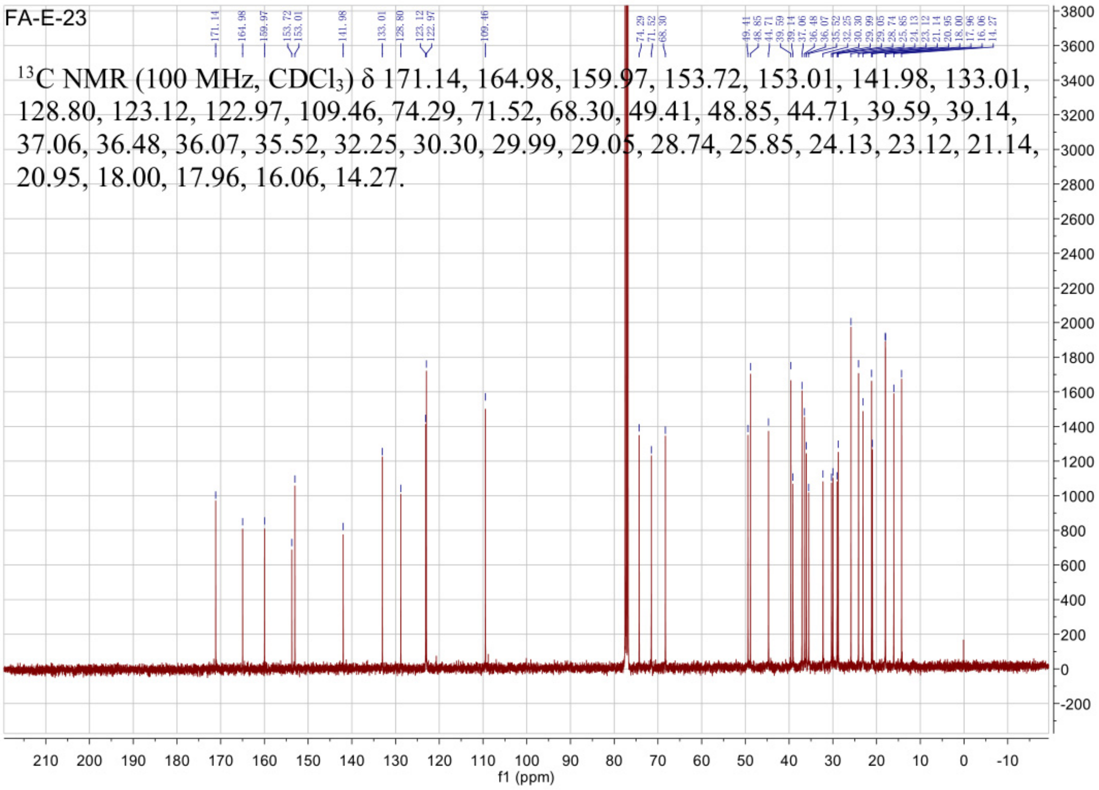

^{13}C NMR of compound FA-26

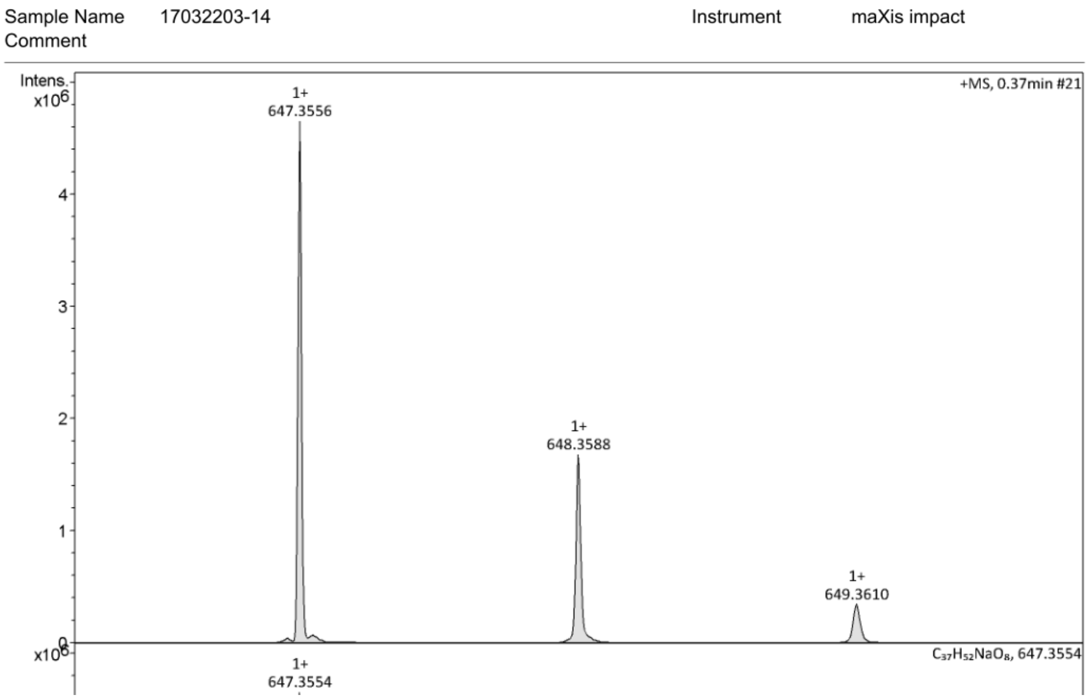

HRMS of compound FA-26
